# Global Trend in Pancreatic Cancer Prevalence Rates Through 2040: An Illness‐Death Modeling Study

**DOI:** 10.1002/cam4.70318

**Published:** 2024-10-23

**Authors:** Zeinab Hesami, Meysam Olfatifar, Amir Sadeghi, Mohammad Reza Zali, Samira Mohammadi‐Yeganeh, Mohammad Amin Habibi, Mohammad Reza Ghadir, Hamidreza Houri

**Affiliations:** ^1^ Foodborne and Waterborne Diseases Research Center Research Institute for Gastroenterology and Liver Diseases, Shahid Beheshti University of Medical Sciences Tehran Iran; ^2^ Department of Microbiology School of Medicine, Shahid Beheshti University of Medical Sciences Tehran Iran; ^3^ Gastroenterology and Hepatology Diseases Research Center Qom University of Medical Sciences Qom Iran; ^4^ Gastroenterology and Liver Diseases Research Center Research Institute for Gastroenterology and Liver Diseases, Shahid Beheshti University of Medical Sciences Tehran Iran; ^5^ Cellular and Molecular Biology Research Center Shahid Beheshti University of Medical Sciences Tehran Iran; ^6^ Department of Medical Biotechnology School of Advanced Technologies in Medicine, Shahid Beheshti University of Medical Sciences Tehran Iran; ^7^ Clinical Research Development Center Qom University of Medical Sciences Qom Iran

**Keywords:** global burden of disease, illness‐death multi‐state model, pancreatic cancer, percentage changes, prevalence

## Abstract

**Background:**

Despite remarkable progress in contemporary medical technology and enhanced survival outcomes for various cancer types, pancreatic cancer (PC) continues to stand out as a particularly deadly gastrointestinal malignancy. Given a persistent rise in both incidence and the corresponding mortality rates of PC globally, evaluations of PC burden by sex are of great importance. Here, we used the illness‐death multi‐state model (IDM) to forecast the prevalence of PC through the year 2040.

**Methods:**

IDM was established based on obtainable data to predict the future prevalence of PC on global, regional, and national scales from 2019 to 2040. Analyses were also performed regarding sex and 95% confidence intervals (CIs) are presented for all estimates.

**Results:**

The projected prevalence rate for 2040 is anticipated to be 6.093 ([95% CI 5.47–6.786] per 100,000) worldwide, indicating a significant increase of 31.45% since 1990, and a 12.29% increase since 2019. The estimated average annual increase since 2020 was 0.5%. Considering sex differences, females are expected to have a steeper slope in prevalence rate than males. Intriguingly, when considering the percentage changes between the periods of 2019–2040 and 1990–2019 for both sexes, females exhibited 29% and 11% increase relative to males (2.6‐fold greater increase).

**Conclusions:**

By 2040, it is predicted that the prevalence of PC will increase globally, with women being at higher risk of developing the disease. Considering the percentage changes, regions with lower socioeconomic status are anticipated to face a greater risk of experiencing PC compared to other geographical areas.

**Abbreviations**
AAPC: average annual percentage change
AIR: age‐adjusted incidence rate
ASIR: age‐standardized incidence rate
ASMR: specific mortality rate
ASPR: age‐standardized prevalence rate
CI: confidence interval
GBD: global burden of disease
GHDx: global health data exchange
GI: Gastrointestinal
HDI: human development index
IDM: illness‐death multi‐state model
IHME: institute for health metrics and evaluation
LAC: Latin America and the Caribbean
ODE: ordinary differential equation
PC: pancreatic cancer
PDAC: pancreatic ductal adenocarcinoma
SEER: surveillance, epidemiology, and end results
WHO: world health organization

## 1 Introduction

Although pancreatic cancer (PC) is the 12th most common type of cancer, it is the 7th leading cause of cancer‐related deaths worldwide particularly in developed nations [1]. Remarkably, it is projected to become the second leading cause of cancer deaths in the United States by 2030, surpassing breast cancer [2]. More importantly, pancreatic ductal adenocarcinoma (PDAC), which accounts for almost 90% of all diagnosed pancreatic tumors [3], ranks in fourth place in mortality among malignancies worldwide [4]. The dismal prognosis with a 5‐year relative overall survival of only 13% among patients suffering from PC, makes this disease the most lethal form of tumor in the gastrointestinal (GI) tract [5]. The manifestation of nonspecific clinical symptoms only at the advanced stages along with the lack of sensitive biomarkers lead to poor outcomes [6]. As a consequence of delayed diagnosis and/or misdiagnosis, the tumor cells often spread to distant organs leading to multi‐organ metastasis that makes the surgical resection almost impossible [7]. Furthermore, the intrinsic aggressive features and high resistance in response to conventional chemotherapy as well as the high recurrence rate make PC particularly hard to treat [8, 9].

According to the GLOBOCAN estimates, it is expected that the worldwide incidence and mortality rate of PC rise by more than 75% and 80% in both low‐human development index (HDI) and high‐HDI regions up until 2040, respectively [10]. This anticipated pattern of increase in the upcoming years could be partly justified as a result of demographic aging, which is one of the major risk factors [11]. Another non‐modifiable risk factor for developing PC is sex, with males being slightly more susceptible than females. The disparity between the two sexes may be partly due to the lifestyle differences between them (e.g., men tend to smoke cigarettes and drink alcohol at higher rates than women) [12]. Noteworthy, while the incidence and characteristics of PC can vary between different geographical locations, the high mortality rate of PC remains consistent regardless of the region. This is an important factor to consider when studying and addressing this disease [1].

Regarding the addressed health issues in the management of PC and the elevated rate of new cases reported by the Surveillance, Epidemiology, and End Results (SEER) Program, this is an urgent need for more up‐to‐date population‐based international studies [13]. Moreover, the geographical variation observed in PC highlights the importance of the global burden of disease (GBD) investigation [1, 14]. Altogether, this could be a big step forward to improve the quality of healthcare services along with informing practitioners and health policy‐makers to take appropriate measures for controlling the disease in the future. Hence, in this study, we aimed to project the future trends in PC prevalence considering sex by 2040 using the IDM [15].

## 2 Methods

### 2.1 Data Source

A dynamic simulation model was employed to forecast the future prevalence of PC. To populate our models, we required age‐standardized prevalence rates (ASPR), age‐standardized incidence rates (ASIR), age‐standardized all‐cause and specific mortality rates (ASMR), along with epidemiological data pertaining to the population size of each geographical unit. Thus, the sex‐specific data on aforementioned rates from 1990 to 2019 at global, regional, and national levels for males, females, and both sexes, were obtained from the Global Health Data Exchange (GHDx) query tool at the Institute for Health Metrics and Evaluation (IHME), available at https://vizhub.healthdata.org/gbd‐results/. Subsequently, the population data were acquired from additional freely accessible GHDx query tools, available at https://vizhub.healthdata.org/population‐forecast/.

### 2.2 Statistical Analysis

#### 2.2.1 Modeling

To estimate the future trajectory of PC prevalence at each geographical level, we employed the IDM, a robust multistate model enabling the modeling of population disease burden by leveraging the interplay between morbidity and mortality [15]. Constructed upon differential equations, this model facilitates the estimation of the future status of the disease. This approach offers a more accurate estimation of the future prevalence of PC compared to previous studies by accounting for fluctuations in incidence and mortality rates [16]. Moreover, our model provides more dynamic and realistic estimate of PC prevalence by considering the interplay between prevalence, incidence, and mortality rates [17]. We utilized existing data to fit models and formulate predictions, customizing distinct IDMs for each geographic and sex‐specific subset within the GBD dataset. Finally, we presented models on a global scale, and for 21 regions, covering 195 countries, since population and fertility data were available for only 195 countries [18]. All calculations were performed with coverage probability of 95% confidence interval (CI).

#### 2.2.2 Projection

In this study, we aimed to forecast the future prevalence of PC up to 2040. Thereby, two ordinary differential equations (ODEs) were employed as follows to establish a relationship among epidemiological indices, subsequently facilitating the estimation of disease prevalence between the predicted period during 2019–2040 [19, 20]:

$$S_{t+1}=S_{t}-i_{t}S_{t}-b_{t}S_{t}$$

$$I_{t+1}=I_{t}+i_{t}S_{t}-b1_{t}I_{t}$$

In the formula, “*S*” represents the number of susceptible individuals, “*I*” denotes the number of people suffering from PC, and the absorbing state corresponds to death, where individuals in other states can transition. Additionally, the $i_{t}$, $b_{t}$ and $b1_{t}$ signify transition probabilities from the susceptible state to the illness state, from the illness state to the death state, and from the susceptible state to the death state at a specific time, respectively. All statistical analysis was performed in the R software package.

## 3 Results

### 3.1 Overall Global Forecasted PC Prevalence

**FIGURE 1:**
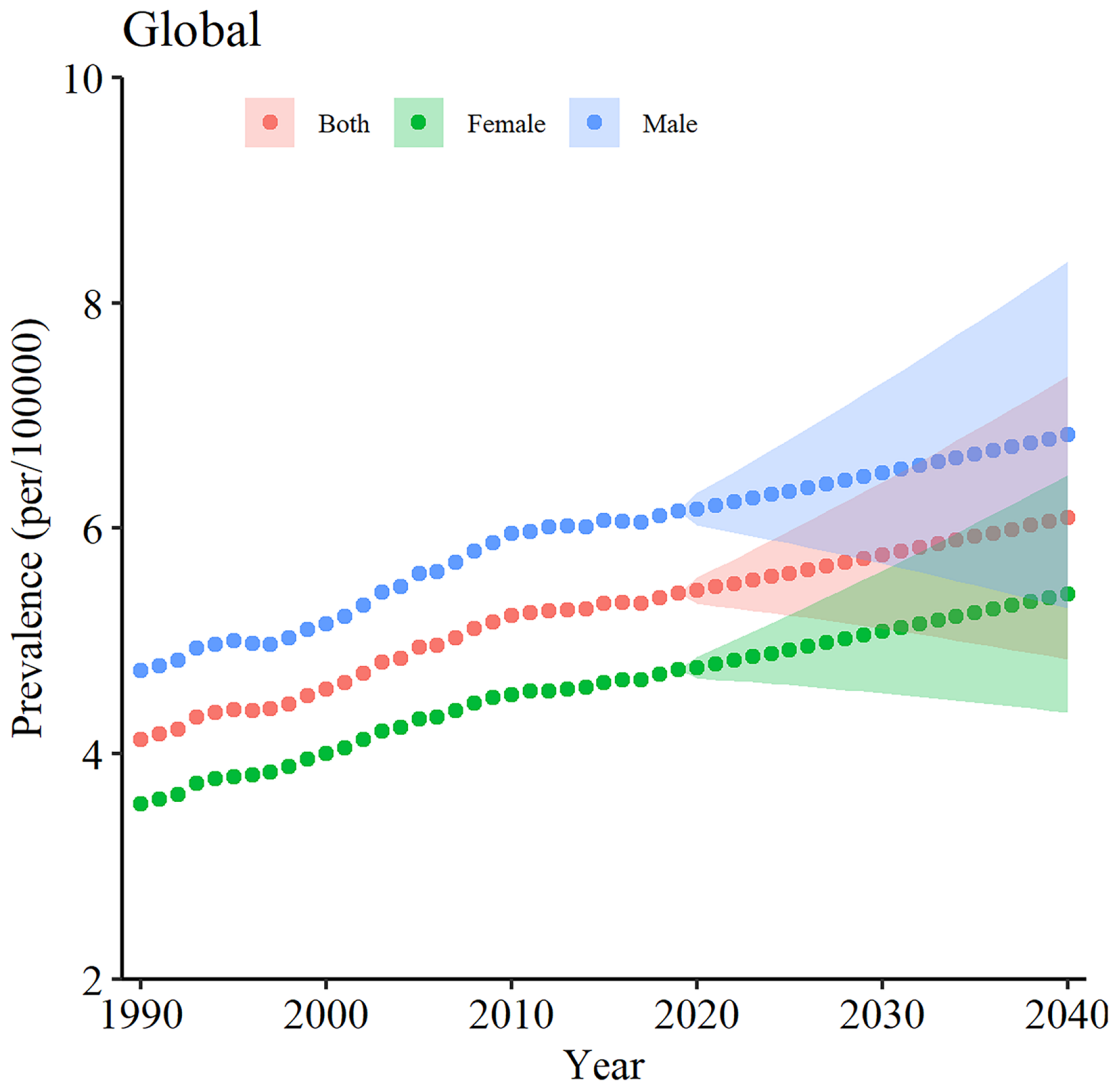
Overall forecast of global PC disease prevalence per 100,000 for male (blue lines), female (green lines), and both sexes (red lines). The halo effect observed in each scatter plot accurately represents projections that extend across the temporal span from 2019 to 2040 with 95% confidence intervals. PC, pancreatic cancer.

Figure 1 illustrates the projected global prevalence trend of PC until 2040, categorized by sex. As depicted, the ASPR of PC will gradually increase globally across both male and female populations, as well as the entire population. By 2040, it was estimated that the ASPR of PC will reach approximately 6.09 ([95% CI 5.47–6.786] per 100,000) within the overall population. Subsequently, the percentage changes from 1990 to 2019 and 2019 to 2040 were estimated to be 31.45% and 12.29%, respectively (Table 1). Additionally, throughout the entire period spanning from 2019 to 2040, the estimated prevalence rate of PC in men is expected to surpass that in women, similar to the pattern observed between 1990 and 2019 (Tables 2 and 3; Figure 1). However, the percentage changes in women, from 1990 to 2019 and from 2019 to 2040, demonstrated an increase of 11% and 29% compared to men, respectively (Tables 2 and 3). In males, the estimated ASPR in 2040 will be 6.828 ([95% CI 6.069–7.681] per 100,000), with an increase of 11.04% between 2019 and 2040 (Table 2). Remarkably, while the estimated ASPR among females was projected to reach 5.42 ([95% CI 4.895–6.002] per 100,000) in 2040, the calculation of the percentage change for PC from 2019 to 2040 indicated a slightly higher increase (14.26%) compared to males (Tables 2 and 3; Figure 3b).

**TABLE 1:** Predicted prevalence of PC from 2020 to 2040 per 100,000 population reported by 5‐year intervals and the corresponding percentage change in PC prevalence for 1990–2019 and 2019–2040, globally and for 21 GBD regions in overall population.

	ASPR, 2020	ASPR, 2025	ASPR, 2030	ASPR, 2035	ASPR, 2040	Percentage change (1990–2019)	Percentage change (2019–2040)
Global	5.448 (5.386–5.51)	5.602 (5.409–5.802)	5.761 (5.43–6.113)	5.925 (5.45–6.441)	6.093 (5.47–6.786)	31.45631768	12.29155567
Central Asia	4.407 (4.296–4.521)	4.32 (3.992–4.675)	4.235 (3.706–4.84)	4.151 (3.439–5.011)	4.07 (3.192–5.188)	129.4552361	−7.916674391
Central Europe	8.278 (8.116–8.442)	8.569 (8.065–9.105)	8.871 (8.006–9.829)	9.183 (7.948–10.611)	9.507 (7.889–11.456)	27.27798082	15.61054981
Eastern Europe	6.101 (5.603–6.643)	5.603 (4.307–7.288)	5.145 (3.299–8.025)	4.725 (2.526–8.84)	4.34 (1.934–9.739)	21.05956254	−31.08054446
Australasia	8.334 (8.108–8.566)	8.53 (7.837–9.284)	8.73 (7.565–10.075)	8.935 (7.302–10.934)	9.145 (7.047–11.867)	24.05433629	9.831235837
High‐income Asia Pacific	10.5 (10.305–10.699)	10.017 (9.454–10.613)	9.556 (8.666–10.537)	9.116 (7.943–10.462)	8.697 (7.28–10.388)	24.07071737	−18.19689508
High‐income North America	9.331 (9.176–9.489)	9.284 (8.817–9.776)	9.237 (8.466–10.08)	9.191 (8.128–10.393)	9.145 (7.803–10.717)	21.57127113	−2.300349259
Southern Latin America	8.274 (8.024–8.531)	8.737 (7.947–9.604)	9.225 (7.861–10.826)	9.742 (7.775–12.206)	10.287 (7.689–13.762)	26.53554757	26.16080513
Western Europe	9.162 (9.001–9.326)	8.919 (8.444–9.421)	8.682 (7.915–9.524)	8.452 (7.419–9.628)	8.227 (6.954–9.735)	32.53797176	−10.94565609
Andean Latin America	4.012 (3.872–4.157)	4.331 (3.882–4.832)	4.675 (3.886–5.626)	5.048 (3.889–6.552)	5.449 (3.892–7.63)	168.3107445	38.43488258
Caribbean	4.01 (3.89–4.134)	4.104 (3.738–4.507)	4.201 (3.586–4.921)	4.3 (3.44–5.374)	4.401 (3.3–5.869)	223.7059783	10.90478112
Central Latin America	4.242 (4.131–4.356)	4.785 (4.408–5.195)	5.398 (4.698–6.202)	6.089 (5.007–7.405)	6.868 (5.335–8.842)	27.42925662	65.72212431
Tropical Latin America	4.689 (4.592–4.788)	4.707 (4.412–5.021)	4.724 (4.235–5.27)	4.742 (4.065–5.531)	4.76 (3.902–5.806)	24.12891613	1.253435529
North Africa and Middle East	4.236 (4.195–4.278)	4.715 (4.575–4.86)	5.249 (4.988–5.524)	5.843 (5.437–6.279)	6.504 (5.927–7.137)	104.3317885	56.57407372
South Asia	2.163 (2.102–2.225)	2.447 (2.242–2.671)	2.769 (2.389–3.21)	3.133 (2.544–3.859)	3.545 (2.71–4.639)	104.1977898	67.64412555
East Asia	4.686 (4.623–4.751)	5.705 (5.469–5.951)	6.945 (6.467–7.458)	8.454 (7.646–9.348)	10.292 (9.039–11.718)	85.45130511	129.340114
Oceania	1.862 (1.845–1.879)	1.983 (1.928–2.039)	2.112 (2.014–2.214)	2.249 (2.104–2.404)	2.395 (2.197–2.61)	41.56778211	30.37931129
Southeast Asia	3.335 (3.299–3.371)	3.734 (3.612–3.861)	4.181 (3.952–4.424)	4.682 (4.324–5.069)	5.242 (4.731–5.809)	84.4994384	60.84487205
Central Sub‐Saharan Africa	2.087 (2.062–2.112)	2.417 (2.329–2.508)	2.799 (2.629–2.98)	3.242 (2.969–3.541)	3.756 (3.352–4.208)	27.86489339	85.92026432
Eastern Sub‐Saharan Africa	2.162 (2.146–2.179)	2.478 (2.419–2.539)	2.84 (2.727–2.958)	3.255 (3.073–3.447)	3.73 (3.464–4.017)	51.57360041	77.71598805
Southern Sub‐Saharan Africa	4.362 (3.894–4.886)	4.185 (2.949–5.939)	4.015 (2.222–7.255)	3.853 (1.674–8.868)	3.696 (1.26–10.841)	−0.695935808	−15.5488894
Western Sub‐Saharan Africa	3.37 (3.325–3.416)	3.76 (3.605–3.921)	4.195 (3.907–4.504)	4.68 (4.234–5.173)	5.221 (4.588–5.941)	101.8327318	58.9078446

*Note:* Data in parentheses are 95% uncertainty intervals.

Abbreviations: ASPR, age‐standardized prevalence rate; GBD, Global Burden of Disease; PC, pancreatic cancer.

**TABLE 2:** Predicted prevalence of PC from 2020 to 2040 per 100,000 population reported by 5‐year intervals and the corresponding percentage change in PC prevalence for 1990–2019 and 2019–2040, globally and for 21 GBD regions in male population.

	ASPR, 2020	ASPR, 2025	ASPR, 2030	ASPR, 2035	ASPR, 2040	Percentage change (1990–2019)	Percentage change (2019–2040)
Global	6.172 (6.096–6.249)	6.33 (6.092–6.577)	6.492 (6.084–6.926)	6.657 (6.077–7.294)	6.828 (6.069–7.681)	29.89103324	11.04291545
Central Asia	5.37 (5.233–5.509)	5.168 (4.774–5.595)	4.974 (4.351–5.687)	4.788 (3.964–5.783)	4.608 (3.612–5.88)	126.1904846	−14.6548582
Central Europe	10.3 (10.069–10.535)	10.557 (9.845–11.32)	10.821 (9.617–12.175)	11.091 (9.392–13.097)	11.368 (9.173–14.089)	23.17136354	10.92618448
Eastern Europe	8.186 (7.413–9.04)	7.173 (5.281–9.744)	6.285 (3.746–10.546)	5.508 (2.656–11.421)	4.826 (1.883–12.371)	17.00282452	−43.58754075
Australasia	9.298 (8.993–9.614)	9.477 (8.548–10.507)	9.659 (8.114–11.498)	9.845 (7.701–12.586)	10.034 (7.308–13.778)	19.48247418	7.863465327
High‐income Asia Pacific	12.218 (11.954–12.487)	11.422 (10.678–12.218)	10.678 (9.53–11.965)	9.983 (8.504–11.719)	9.332 (7.588–11.478)	13.14483352	−24.92169873
High‐income North America	10.446 (10.259–10.636)	10.545 (9.973–11.15)	10.645 (9.688–11.697)	10.746 (9.41–12.272)	10.848 (9.139–12.876)	18.02164247	4.168370618
Southern Latin America	8.996 (8.679–9.324)	9.557 (8.555–10.676)	10.152 (8.42–12.242)	10.785 (8.285–14.04)	11.458 (8.152–16.104)	21.76538083	29.49008242
Western Europe	9.756 (9.576–9.939)	9.395 (8.872–9.95)	9.048 (8.213–9.969)	8.714 (7.602–9.99)	8.392 (7.036–10.01)	20.70028125	−14.89184188
Andean Latin America	3.761 (3.652–3.874)	3.99 (3.641–4.371)	4.232 (3.627–4.938)	4.489 (3.611–5.58)	4.761 (3.596–6.305)	140.5693771	28.46308462
Caribbean	4.433 (4.282–4.59)	4.471 (4.016–4.977)	4.509 (3.762–5.405)	4.548 (3.522–5.871)	4.586 (3.298–6.378)	229.3857932	4.235745956
Central Latin America	4.392 (4.238–4.552)	5.162 (4.624–5.763)	6.067 (5.037–7.309)	7.131 (5.486–9.271)	8.382 (5.974–11.76)	33.56468621	97.37604016
Tropical Latin America	5.142 (5.021–5.266)	5.114 (4.751–5.504)	5.086 (4.492–5.759)	5.058 (4.246–6.027)	5.031 (4.013–6.307)	23.71252904	−2.739946978
North Africa and Middle East	4.96 (4.903–5.017)	5.468 (5.278–5.665)	6.029 (5.678–6.4)	6.647 (6.109–7.232)	7.328 (6.572–8.171)	95.08240941	50.26962746
South Asia	2.232 (2.172–2.293)	2.518 (2.314–2.739)	2.841 (2.464–3.276)	3.205 (2.622–3.918)	3.617 (2.791–4.687)	79.26705056	65.90415186
East Asia	5.995 (5.911–6.081)	7.139 (6.834–7.458)	8.501 (7.897–9.152)	10.123 (9.123–11.233)	12.055 (10.541–13.787)	94.76210057	108.8537441
Oceania	2.146 (2.12–2.171)	2.267 (2.185–2.352)	2.395 (2.25–2.549)	2.531 (2.318–2.763)	2.674 (2.387–2.995)	37.0576473	26.15644765
Southeast Asia	3.494 (3.438–3.55)	3.89 (3.703–4.087)	4.332 (3.986–4.708)	4.823 (4.289–5.424)	5.371 (4.616–6.249)	71.21988534	57.06851254
Central Sub‐Saharan Africa	2.637 (2.606–2.668)	2.999 (2.892–3.11)	3.41 (3.208–3.626)	3.878 (3.557–4.228)	4.41 (3.945–4.931)	20.3817037	72.06636496
Eastern Sub‐Saharan Africa	2.403 (2.384–2.422)	2.69 (2.623–2.757)	3.01 (2.886–3.14)	3.369 (3.176–3.575)	3.771 (3.494–4.071)	36.95528989	60.92474385
Southern Sub‐Saharan Africa	5.211 (4.667–5.819)	4.822 (3.432–6.777)	4.463 (2.511–7.93)	4.13 (1.836–9.286)	3.821 (1.343–10.875)	0.63798293	−28.29685185
Western Sub‐Saharan Africa	3.192 (3.149–3.237)	3.476 (3.332–3.627)	3.785 (3.523–4.067)	4.122 (3.725–4.561)	4.489 (3.939–5.115)	74.18520182	43.56823533

*Note:* Data in parentheses are 95% uncertainty intervals.

Abbreviations: ASPR, age‐standardized prevalence rate; GBD, Global Burden of Disease; PC, pancreatic cancer.

**TABLE 3:** Predicted prevalence of PC from 2020 to 2040 per 100,000 population reported by 5‐year intervals and the corresponding percentage change in PC prevalence for 1990–2019 and 2019–2040, globally and for 21 GBD regions in female population.

	ASPR, 2020	ASPR, 2025	ASPR, 2030	ASPR, 2035	ASPR, 2040	Percentage change (1990–2019)	Percentage change (2019–2040)
Global	4.765 (4.715–4.817)	4.921 (4.761–5.087)	5.082 (4.805–5.375)	5.249 (4.85–5.68)	5.42 (4.895–6.002)	33.37417557	14.26421972
Central Asia	3.641 (3.55–3.734)	3.635 (3.361–3.93)	3.629 (3.18–4.142)	3.623 (3.008–4.365)	3.617 (2.845–4.6)	130.0082217	−0.698268008
Central Europe	6.489 (6.375–6.604)	6.753 (6.394–7.132)	7.027 (6.408–7.707)	7.313 (6.422–8.329)	7.611 (6.435–9.002)	31.75538016	18.13835637
Eastern Europe	4.527 (4.245–4.827)	4.379 (3.592–5.338)	4.235 (3.03–5.919)	4.096 (2.556–6.566)	3.962 (2.155–7.284)	21.63644069	−13.9761656
Australasia	7.427 (7.211–7.651)	7.657 (6.988–8.389)	7.893 (6.764–9.211)	8.137 (6.545–10.115)	8.388 (6.334–11.108)	28.20263403	13.28506821
High‐income Asia Pacific	8.91 (8.741–9.082)	8.672 (8.174–9.201)	8.441 (7.638–9.328)	8.216 (7.136–9.458)	7.996 (6.667–9.591)	34.53437194	−10.94308966
High‐income North America	8.311 (8.167–8.457)	8.145 (7.718–8.596)	7.983 (7.289–8.743)	7.823 (6.882–8.893)	7.667 (6.498–9.047)	24.43271553	−8.622975155
Southern Latin America	7.589 (7.376–7.807)	7.946 (7.28–8.674)	8.321 (7.176–9.648)	8.713 (7.072–10.734)	9.123 (6.969–11.943)	31.62030547	21.68729343
Western Europe	8.569 (8.417–8.724)	8.421 (7.968–8.9)	8.276 (7.538–9.086)	8.133 (7.13–9.278)	7.993 (6.744–9.474)	45.22488754	−7.282678925
Andean Latin America	4.229 (4.037–4.429)	4.631 (4.013–5.343)	5.071 (3.982–6.457)	5.552 (3.949–7.806)	6.08 (3.917–9.437)	196.4701512	47.03825261
Caribbean	3.608 (3.507–3.713)	3.755 (3.438–4.101)	3.908 (3.367–4.536)	4.067 (3.297–5.018)	4.233 (3.228–5.551)	217.6179089	18.94236212
Central Latin America	4.099 (4.009–4.191)	4.455 (4.16–4.772)	4.843 (4.312–5.439)	5.264 (4.469–6.199)	5.721 (4.632–7.066)	21.99563769	41.53927619
Tropical Latin America	4.271 (4.189–4.354)	4.32 (4.069–4.586)	4.369 (3.95–4.833)	4.419 (3.833–5.095)	4.47 (3.72–5.371)	25.18657902	4.710942663
North Africa and Middle East	3.47 (3.441–3.499)	3.91 (3.81–4.013)	4.406 (4.217–4.603)	4.965 (4.667–5.281)	5.594 (5.166–6.058)	118.7298474	64.98363238
South Asia	2.093 (2.027–2.161)	2.374 (2.151–2.62)	2.693 (2.28–3.182)	3.056 (2.416–3.864)	3.467 (2.561–4.693)	144.8686925	69.2730155
East Asia	3.407 (3.325–3.491)	3.973 (3.745–4.216)	4.634 (4.214–5.095)	5.404 (4.741–6.16)	6.303 (5.334–7.448)	73.37105243	89.12314217
Oceania	1.556 (1.543–1.569)	1.677 (1.634–1.721)	1.807 (1.729–1.888)	1.947 (1.831–2.072)	2.098 (1.937–2.273)	48.27635807	36.91610623
Southeast Asia	3.17 (3.147–3.194)	3.569 (3.486–3.653)	4.017 (3.862–4.178)	4.521 (4.277–4.779)	5.089 (4.737–5.467)	99.08111851	64.48318657
Central Sub‐Saharan Africa	1.621 (1.6–1.643)	1.91 (1.832–1.99)	2.25 (2.098–2.412)	2.65 (2.402–2.924)	3.122 (2.75–3.544)	42.75499255	99.54882433
Eastern Sub‐Saharan Africa	1.932 (1.918–1.947)	2.275 (2.223–2.33)	2.68 (2.575–2.788)	3.155 (2.983–3.337)	3.716 (3.456–3.995)	75.13863977	99.21063109
Southern Sub‐Saharan Africa	3.641 (3.192–4.153)	3.649 (2.432–5.477)	3.657 (1.842–7.263)	3.666 (1.394–9.639)	3.674 (1.055–12.796)	−1.361161098	1.677326633
Western Sub‐Saharan Africa	3.542 (3.491–3.593)	4.027 (3.853–4.209)	4.579 (4.249–4.935)	5.207 (4.686–5.786)	5.921 (5.168–6.784)	136.3344395	72.13283556

*Note:* Data in parentheses are 95% uncertainty intervals.

Abbreviations: ASPR, age‐standardized prevalence rate; GBD, Global Burden of Disease; PC, pancreatic cancer.

### 3.2 Forecasted PC Prevalence for Regions

**FIGURE 2:**
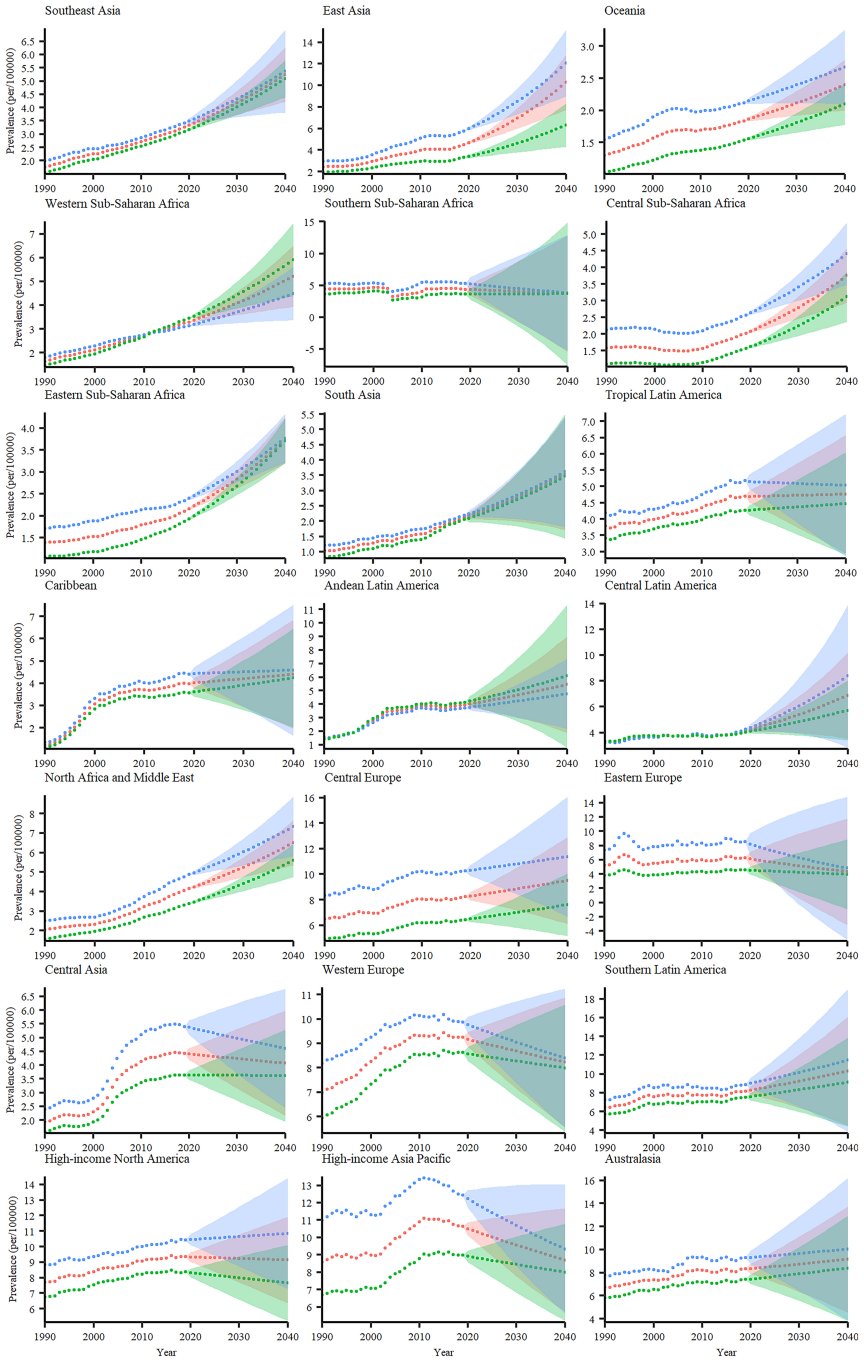
Regional forecast of PC prevalence per 100,000 for male (blue lines), female (green lines), and both sexes (red lines). The halo effect observed in each scatter plot accurately represents projections that extend across the temporal span from 2019 to 2040 with 95% confidence intervals. PC, pancreatic cancer.

Generally, the prevalence of PC displayed an upward trend in most geographical regions (Tables 1, 2, 3; Figure 2). Furthermore, among all regions, the estimated prevalence rates were higher among men compared to women, except for Western Sub‐Saharan Africa and Andean Latin America (Tables 2 and 3; Figure 2). According to the recent projection, East Asia had the highest ASPR in 2040 (10.292 [95% CI 9.039–11.718] per 100,000) among the total population as well as the male population (12.055 [95% CI 10.541–13.787] per 100,000) (Tables 1 and 2). In contrast, Southern Latin America held the highest ASPR among the female population (9.123 [95% CI 6.969–11.943] per 100,000) (Table 3). On the other end of the spectrum, Oceania recorded the lowest ASPR values in 2040 for the male, female, and total populations, with figures of 2.674 ([95% CI 2.387–2.995] per 100,000), 2.098 ([95% CI 1.937–2.273] per 100,000) and 2.395 ([95% CI 2.197–2.61] per 100,000), respectively (Tables 1, 2, 3).

**FIGURE 3:**
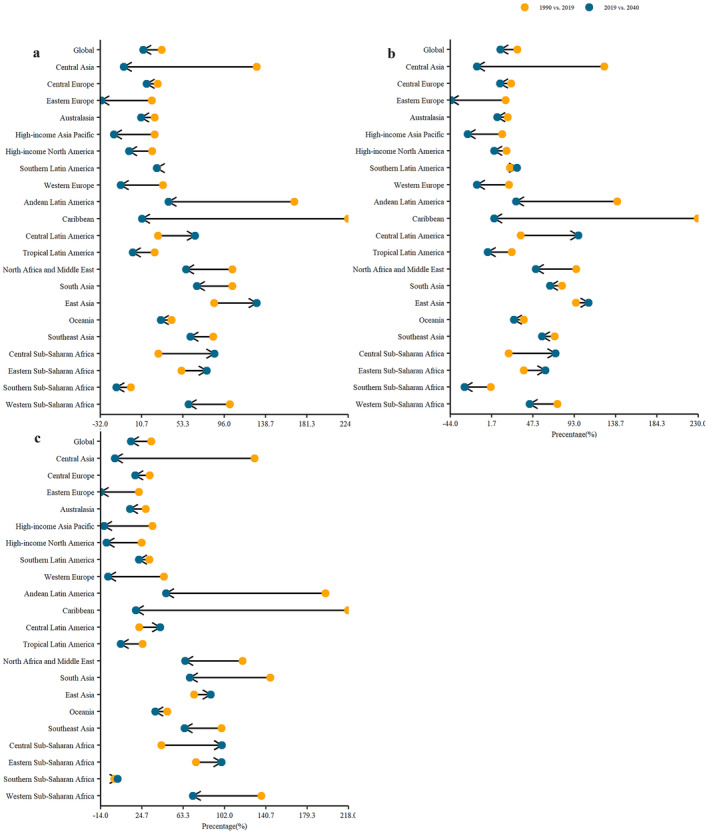
The lollipop plot between the two calculated percentage changes from 1990 to 2019 and 2019 to 2040 for both sexes (a) males (b) and females (c), globally and across 21 regions. Each line represents two time periods and show the change of ASPR increase or decrease during time. ASPR, age‐standardized prevalence rate; PC, pancreatic cancer.

When assessing the percentage changes from 1990 to 2019, the Caribbean represented the most substantial change, surpassing 200% across male, female, and total populations (Tables 1, 2, 3; Figure 3a). On the contrary, Southern Sub‐Saharan Africa emerged as the exclusive region with a negative change, thereby illustrating the declining pattern in prevalence rates over this period among the entire population (Table 1; Figure 2).

In the context of percentage changes from 2019 to 2040, 8 out of the 21 regions exhibited a percentage change exceeding 50% within the overall population. The highest rise, reaching nearly 130%, was observed in East Asia (Table 1; Figure 3b). Notably, a comparable pattern unfolded within the male population, mirroring the trend observed in the total population, where East Asia stood out with the highest percentage change 108.8% from 2019 to 2040 (Table 2). On the other hand, Eastern Europe demonstrated the most conservative percentage change, with a reduction of 31.08% in the total population (Table 1). Likewise, over the same period, the Eastern Europe region presented the highest negative shift for both women and men, registering a percentage change of approximately −14% and −43.6%, respectively (Tables 2 and 3; Figure 3b,c).

Comparing the percentage changes during the intervals of 1990–2019 and 2019–2040, the majority of regions are anticipated to experience a fall between these two periods. However, a few regions were estimated to experience an increase over the same time span (Tables 1, 2, 3; Figure 3). Particularly noteworthy was Central Sub‐Saharan Africa, where there was a substantial nearly three‐fold increase among males as well as in total populations (Tables 1 and 2). Similarly, within the female population, Central Sub‐Saharan Africa demonstrated the most prominent fold change of 2.3 between percentage changes across the two intervals (Table 3).

### 3.3 Trends in the Disease Burden at the National Level

#### 3.3.1 Temporal Trends in PC Prevalence Rate and Percentage Change Within the Overall Population: 1990–2019 and Projections to 2040

In 2019, Greenland had the highest ASPR (14.679 [95% CI 11.940–17.378] per 100,000) of all countries, although the lowest ASPR was observed in Ethiopia (1.06 [95% CI 0.712–1.528] per 100,000). From 1990 to 2019, when considering both sexes combined, Cabo Verde, experienced a remarkable increase of 741.13%, attaining the highest percentage change among all nations, whereas Sweden displayed the highest decline (−10.55%) (Data S1–S5).

Our projection indicated that Cabo Verde will exhibit the highest prevalence rate (28.961 [95% CI 14.864–56.428] per 100,000) in 2040. Subsequently, United Arab Emirates, Greenland, and Canada demonstrated the highest ASPR values following Cabo Verde, with United Arab Emirates recording an ASPR of 27.085 ([95% CI 17.879–41.033] per 100,000), Greenland with 15.519 ([95% CI 11.967–20.124] per 100,000), and Canada with 13.895 ([95% CI 11.628–16.604] per 100,000). By comparison, countries with the lowest prevalence rates of PC were projected to include Lao People's Democratic Republic (Laos) (0.162 [95% CI 0.038–0.703] per 100,000), and Somalia (1.033 [95% CI 0.958–1.115] per 100,000). Furthermore, between 2019 and 2040, approximately 4.6% of all countries are anticipated to exceed a 100% increase, with Cabo Verde demonstrating the most substantial percentage change, surpassing 250%. On the other hand, the lowest percentage change between 2019 and 2040 is expected to be observed in Laos, reaching a reduction of 90.5%. Within the subset of countries exhibiting negative percentage changes, almost 45% were situated within the high‐income super‐region. Nearly 85% of this portion specifically falling under the category of Western Europe, encompassing 73% of the countries within that region (Data S1–S5).

Evaluating the percentage changes between the periods 1990–2019 and 2019–2040, Kazakhstan was forecasted to have a significant drop, transitioning from 462.4% in the initial period to −19.29% in the subsequent period. Conversely, Colombia is predicted to undergo a shift, moving from an 8.93% decline between 1990 and 2019 to a 24.6% rise between 2019 and 2040 (Data [Link], [Link]).

#### 3.3.2 Projected Prevalence Rate and Percentage Change Stratified by Sex

Regarding sex differences, it could be perceived from our findings that there is a noticeable disparity in ASPRs among countries by 2040. Cabo Verde is expected to hold the highest ASPR among men (57.893 [95% CI 28.086–119.333] per 100,000) while Ghana showcased the highest ASPR in women (15.485 [95% CI 10.87–22.061] per 100,000) in 2040. Nevertheless, Laos was found to have the lowest prevalence rate in both men and women, with 0.483 [95% CI (0.209–1.113) per 100,000] and 0.048 [95% CI (0.005–0.43) per 100,000], respectively (Data [Link], [Link]).

When assessing the percentage changes between 1990 and 2019, Cabo Verde presented the highest change in both male (550.15%) and female (899.01%) populations among all nations, however, the female population showed a higher percentage change compared to males. Importantly, the majority of countries manifesting a rise reaching over 100% were positioned within Latin America and the Caribbean for both sexes. Upon comparing sex differences, it becomes apparent that approximately 46% of nations displayed a growth exceeding 100% among females, whereas the proportion for males was nearly 27%. In terms of the comparison of the lowest percentage changes from 1990 to 2019 (exceeding −10%), the countries demonstrating such patterns within the female population included Iceland, Sweden, and Colombia. In the male population, however, the countries were Burundi, Luxembourg, and Sweden (Data [Link], [Link], [Link], [Link], [Link]).

Analyzing the percentage changes between 2019 and 2040 from a sex perspective reveals that around 3.6% and 5.6% of all countries are projected to experience changes exceeding 100% for males and females, respectively. Super‐regions housing countries with the highest growth encompass Sub‐Saharan Africa, Latin America and the Caribbean (LAC), North Africa, and the Middle East, as well as South‐East Asia, East Asia, and Oceania. Among females, the United Republic of Tanzania showcased the highest rise, reaching nearly 140%. Alternatively, within the male subgroup, Cabo Verde exhibited a remarkable change with an increase of over 700%. In the group of nations exhibiting negative percentage changes, Laos is expected to witness the most substantial decline (exceeding −70%), in both males and females compared to changes during 1990–2019. Besides, it is anticipated that among the 195 countries, 51 countries for males and 41 countries for females would presumably experience a negative percentage change. The prominent region exhibiting this negative trend is expected to be Western Europe. Nevertheless, among males, Luxembourg, and, among females, Greece, demonstrated the highest growth within Western Europe, with percentages of 16.83% and 38.36%, respectively (Data [Link], [Link]).

## 4 Discussion

PC, characterized by its rapid fatality, presents a formidable challenge to clinicians in addressing various aspects, including early detection and optimal management. The unfavorable prognosis can be linked to the asymptomatic progression of pancreatic tumors during their initial phases. Typically, these tumors infiltrate nearby tissues and/or metastasize prior to being diagnosed. As a result, the mortality rates of PC closely align with its incidence rates. According to data from the SEER Program, the projected number of new PC cases in the United States for 2024 is expected to reach 66,440, accounting for 3.3% of all recently diagnosed cancer cases. In addition, it is indicated that, by 2024, 8.5% of cancer‐related deaths are attributed to PC among the American population [13].

A notable strength of our study is the methodology employed to project the future prevalence of PC. The IDM is particularly well‐suited for our study on forecasting PC prevalence due to its ability to capture the dynamic transitions between various health states, including healthy, ill, and deceased. This comprehensive representation allows us to model the complexities associated with PC, particularly given its aggressive nature and diverse patient pathways. Unlike simpler models, the IDM accurately reflects real‐world scenarios by incorporating not only the incidence and prevalence of the disease but also periods of remission and the potential impact of evolving treatment options. Additionally, the model's integration of time as a crucial variable enables us to consider shifts in population demographics and advancements in medical technology, making our forecasts more robust and relevant. This depth of analysis ultimately enriches our forecasts and highlights the importance of adaptive healthcare solutions in tackling this challenging disease. Unlike previous studies, our approach offers a more accurate estimation by accounting for fluctuations in incidence and mortality rates [16]. This is crucial because both incidence and mortality rates are subject to change over time due to various factors such as developments in medical science and technology, changes in healthcare policies, and shifts in population demographics. Besides, our model generates a more precise estimation of PC prevalence by considering the interactions between prevalence, incidence, and mortality rates [17]. Traditional models often handle these variables separately, which can result in less reliable estimates. Our approach, however, acknowledges the interconnected nature of these factors. For instance, a rise in PC incidence may be balanced by advances in treatment that decrease mortality rates, leading to stable prevalence. Likewise, variations in mortality rates can impact prevalence regardless of changes in incidence. As a result, the IDM approach enhances the reliability of our predictions.

Projecting into the future until 2040 on a global, regional, and national scale, our study marks the pioneering effort to elucidate the anticipated trends in PC prevalence. Our findings unveiled a general upward trend in the prevalence of PC rate over the course of the study period, globally. While comparing the periods from 1990 to 2019 and from 2019 to 2040, the percentage change in the prevalence rate decreased by more than double during the latter period. Increasing estimated ASPR with a slightly gentler slope could be explained by improvements in the healthcare system for screening and detection of PC at early stages in the future.

Globally, with regard to sex, the prevalence rate among males exhibited a consistent pattern with existing epidemiological data, indicating a higher prevalence compared to females up until 2040 [21]. Nevertheless, the percentage change of the estimated prevalence rate among females was higher than that among males. Furthermore, considering the ASPR in 2040 compared to the ASPR in 2019, the female population exhibited a slightly higher fold change relative to the male population, worldwide. In the United States, the percentage changes between 1990 and 2019 in men and women were 17.04% and 21.96%, respectively. Likewise, based on the nationwide study conducted on the majority of the US population from 2001 to 2018, findings indicated a greater increase in the age‐adjusted incidence rate (aIR) among women compared to men for patients younger than 55 years (average annual percentage change (AAPC) = 2.36% vs. 0.62%) [22]. Intriguingly, based on our recent prediction, a similar pattern to what has been reported in the US population is expected to emerge on a global scale by 2040. In contrast, the pattern of alteration in ASPR between 2019 and 2040 is anticipated to undergo notable changes in the United States. This transformation is particularly noteworthy, with an expected rise of 1.35% for men and a substantial negative change of 13.01% for women.

On a regional scale, consistent with previous reports, the highest prevalence rates between 1990 and 2019 predominantly located in high‐income regions rather than other geographic areas [23]. Noteworthy based on our study, the most substantial decline in percentage changes from 2019 to 2040 were observed in countries characterized by higher HDI scores. The initial increase pattern in the former time period (1990–2019) could potentially stem from a combination of factors. Primarily, the aging population, coupled with unhealthy modern lifestyle behaviors and prevalent metabolic disorders, offers an explanation for this increasing trend in high‐income regions. Additionally, the higher prevalence rates in high‐income regions during the period from 1990 to 2019, might partly be due to the quality and availability of advanced imaging technologies and an augmented emphasis on health awareness. Consequently, the higher self‐awareness of the disease and its symptoms, along with controlling for risk factors could explain the negative percentage change between 2019 and 2040 in countries with higher HDI scores [12].

Between 1990 and 2019, in comparison to the period from 2019 to 2040, there was heterogeneous trend in percentage changes for most of countries, either experiencing decreases or transitioning to negative changes. Among countries representing negative percentage changes from 2019 to 2040, a significant proportion (85%) belonged to Western Europe. Notably, within the Western Europe region, only Sweden, Greece, and Ireland exhibited increasing pattern when comparing percentage changes during 1990–2019 and 2019–2040.

Based on previous studies, Sweden is anticipated to see a substantial growth of 28% in PC incidence by 2030, despite ongoing efforts aimed at developing the novel blood‐based biomarkers for screening of the disease [24, 25]. Furthermore, in our calculations, Sweden is also expected to experience the growth of 15.5% in context of PC prevalence during 2019–2040 comparing to 1990–2019. According to the Global Cancer Observatory, Greece faced a significant burden of pancreatic cancer, ranking among the 10 most common cancers in the country, with an estimated 1000 new cases and 950 deaths in 2020 [23]. Consistent with the observed trend in PC burden in Greece, our projections indicated that Greece is expected to have both the highest prevalence rate and the highest percentage change during 2019–2040 in the Western European region. In the year 2040, Laos, situated in Southeast Asia, is projected to have the lowest prevalence rate for PC in both male and female populations. According to the most recent data from the World Health Organization (WHO) in 2020, PC accounted for 78 deaths, representing only 1.3% of total deaths in Laos [23]. Thus, PC is relatively less prevalent as a health issue in Laos compared to other countries. The main risk factors for PC include smoking, obesity, diabetes, and a family history of the disease [12]. The relatively low burden of PC in Laos may be partly due to the lower prevalence of these risk factors compared to other countries.

PC presents a significant global challenge that necessitates a coordinated international response. Healthcare providers and policymakers should intensify efforts to manage associated risk factors through strategies that promote lifestyle changes and elevate public awareness. Key interventions include dietary and lifestyle modifications, early screening for high‐risk individuals, access to quality healthcare, and the advancement of treatment modalities such as personalized therapy and access to clinical trials. Additionally, models for estimating future cancer burdens are essential for tracking PC incidence and survival rates. The projected global increase in PC, particularly the pronounced rise in prevalence among females compared to males, serves as an urgent warning for clinicians, researchers, and policymakers, underscoring the need for increased vigilance regarding these trends. This situation further emphasizes the necessity for additional research into the risk factors and mechanisms driving these changes. Ultimately, while our study provides crucial epidemiological insights, it also highlights the importance of a multifaceted approach to address the challenges associated with PC. Epidemiological studies are fundamental in revealing the underlying causes of the disease, establishing a foundation for controlling its burden in the future, and guiding the development of more effective diagnostic, preventive, and therapeutic strategies.

In conclusion, the global prevalence of PC is projected to increase by 2040, albeit at a slower pace compared to the 1990–2019 period. Contrary to the current trend where PC is more prevalent in men than in women, our findings suggest women might be at a higher risk of developing PC in the future. On the other hand, based on percentage changes from 2019 to 2040, among different continents, Africa and Asia are at greater risk of confronting the disease. To summarize, PC is a global challenge that demands a coordinated, global response. Healthcare providers and policymakers could intensify their efforts to manage the associated risk factors. This could encompass initiatives such as promoting lifestyle changes and conducting public awareness. To tackle the challenges associated with PC, epidemiological studies can play a pivotal role in unveiling its underlying causes and, consequently, form the groundwork to control the disease burden in the future.

## Author Contributions

**Zeinab Hesami:** data curation (equal), investigation (equal), writing – original draft (lead). **Meysam Olfatifar:** conceptualization (equal), data curation (lead), formal analysis (lead), funding acquisition (lead), methodology (lead), resources (lead), validation (equal). **Amir Sadeghi:** project administration (equal), resources (equal), validation (equal). **Mohammad Reza Zali:** data curation (supporting), project administration (supporting), resources (equal), supervision (supporting), validation (equal). **Samira Mohammadi‐Yeganeh:** methodology (equal), validation (equal), writing – original draft (equal). **Mohammad Amin Habibi:** investigation (equal). **Mohammad Reza Ghadir:** project administration (supporting), supervision (supporting), validation (equal). **Hamidreza Houri:** conceptualization (equal), data curation (equal), investigation (equal), methodology (equal), project administration (lead), supervision (lead), validation (equal), visualization (equal), writing – review and editing (lead).

## Conflicts of Interest

The authors declare no conflicts of interest.

## Supporting information


Data S1.

Data S2.

Data S3.

Data S4.

Data S5.

## Data Availability

All data generated or analyzed during this study are included in this published article and related supplementary data.
