# Correction to “Global Trend in Pancreatic Cancer Prevalence Rates Through 2040: An Illness‐Death Modeling Study”

**DOI:** 10.1002/cam4.70417

**Published:** 2024-11-18

Hesami Z, Olfatifar M, Sadeghi A, Zali MR, Mohammadi‐Yeganeh S, Habibi MA, Ghadir MR, Houri H. Global Trend in Pancreatic Cancer Prevalence Rates Through 2040: An Illness‐Death Modeling Study. *Cancer Med*. 2024 Oct;13(20):e70318. https://doi.org/10.1002/cam4.70318

The Ethical Code Acknowledgment is missing in the published paper. The Ethics Statement, “This project has been ethically approved by the Ethics Committee at Qom University of Medical Sciences, Qom, Iran (No. IR.MUQ.REC.1402.186).” should be placed after the Acknowledgment section.

We apologize for this error.

