# Supplementary material for: Global Trend in Pancreatic Cancer Prevalence Rates Through 2040: An Illness‐Death Modeling Study
Source: Cancer Med. 2024 Oct 23;13(20):e70318. doi: 10.1002/cam4.70318 (PMC11497012; doi:10.1002/cam4.70318)
Supplement: Supplementary file 1 — Data S1. [file CAM4-13-e70318-s003.docx]

**Global Trend in Pancreatic Cancer Prevalence Rates Through 2040; an Illness-Death Multi-state Modelling**

Table of Contents

[Maps 2](#_Toc165449710)

[Andean Latin America 2](#_Toc165449711)

[Australasia 5](#_Toc165449712)

[Caribbean 7](#_Toc165449713)

[Central Asia 11](#_Toc165449714)

[Central Europe 14](#_Toc165449715)

[Central Latin America 18](#_Toc165449716)

[Central Sub-Saharan Africa 21](#_Toc165449717)

[East Asia 24](#_Toc165449718)

[Eastern Europe 26](#_Toc165449719)

[Eastern Sub-Saharan Africa 29](#_Toc165449720)

[High-income Asia Pacific 33](#_Toc165449721)

[High-income North America 35](#_Toc165449722)

[North Africa and Middle East 37](#_Toc165449723)

[Oceania 43](#_Toc165449724)

[South Asia 46](#_Toc165449725)

[Southeast Asia 48](#_Toc165449726)

[Southern Latin America 52](#_Toc165449727)

[Southern Sub-Saharan Africa 54](#_Toc165449728)

[Tropical Latin America 57](#_Toc165449729)

[Western Europe 59](#_Toc165449730)

[Western Sub-Saharan Africa 65](#_Toc165449731)

In this section, the results of the 21 studied regions are represented by considering their constituent countries. The results of the regions are arranged in alphabetical order.

# Maps


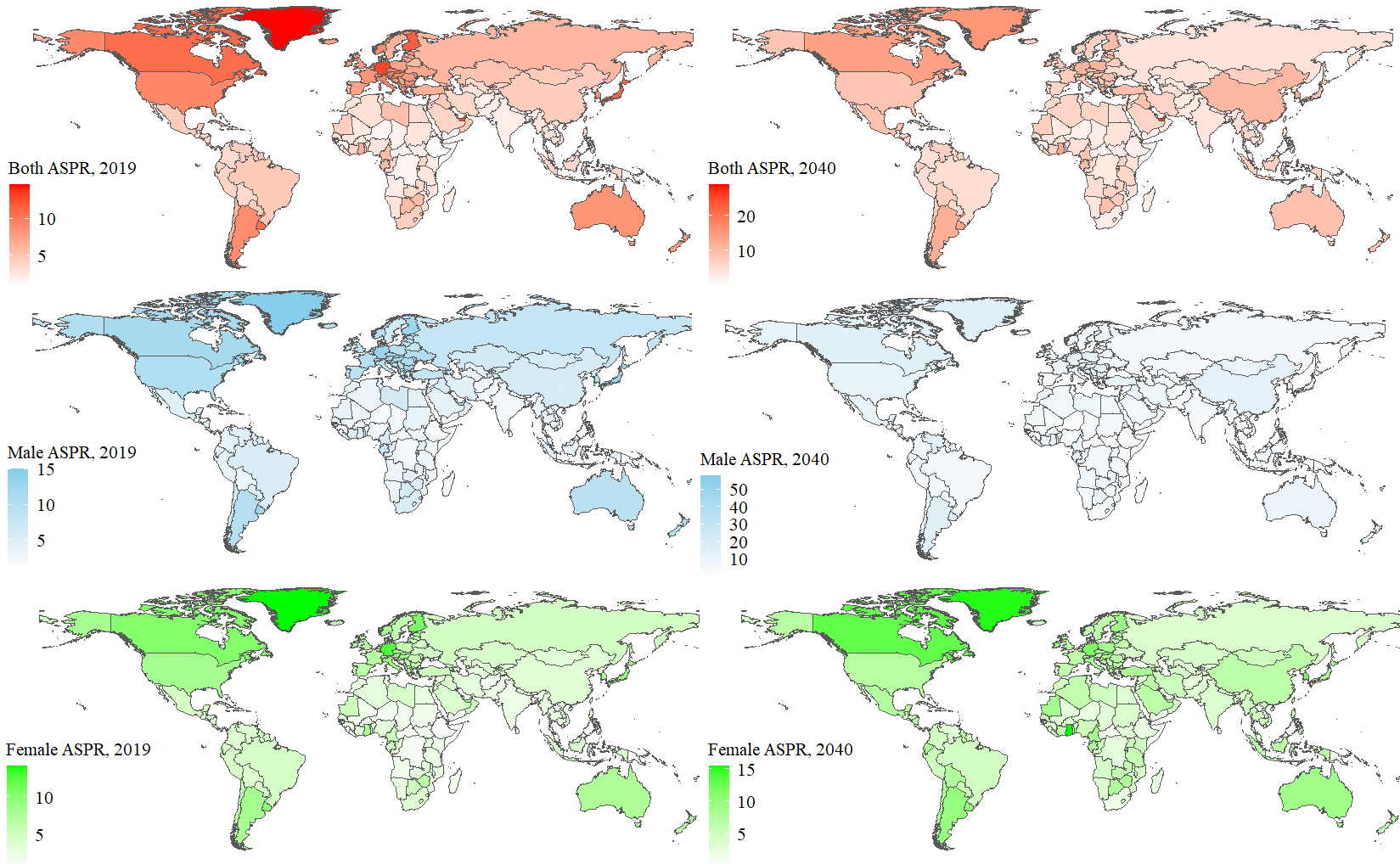


Supplemental Figure 1. Global GBD and temporal trends of PC prevalence in 195 countries or territories. The ASPR per 100,000 population for the overall, male, and female populations in 2019 and 2040 are depicted using red, blue, and green maps, respectively. PC, pancreatic cancer; ASPR, age- standardized prevalence rate; GBD, Global Burden of Disease.

# Andean Latin America

| Supplemental Table 1: Age-standardized prevalence rates (ASPR) from 2020 to 2040, and percentage changes for the time periods 1990 to 2019 and 2019 to 2040, for Andean Latin America. | | | | | | | | |
| --- | --- | --- | --- | --- | --- | --- | --- | --- |
| Group | Country | 2020 | 2025 | 2030 | 2035 | 2040 | 1990 vs. 2019 | 2019 vs. 2040 |
| Both | Bolivia (Plurinational State of) | 3.766(3.568-3.975) | 4.402(3.726-5.201) | 5.145(3.881-6.82) | 6.014(4.042-8.946) | 7.029(4.21-11.736) | 109.1184 | 94.15178 |
| Both | Ecuador | 4.217(3.964-4.486) | 4.822(3.984-5.836) | 5.514(3.994-7.612) | 6.305(4.002-9.933) | 7.209(4.01-12.962) | 206.9812 | 77.38227 |
| Both | Peru | 4.005(3.745-4.283) | 4.137(3.363-5.09) | 4.274(3.011-6.067) | 4.415(2.694-7.233) | 4.56(2.411-8.624) | 172.5063 | 14.56473 |
| Male | Bolivia (Plurinational State of) | 3.473(3.323-3.629) | 4.033(3.52-4.62) | 4.683(3.722-5.892) | 5.439(3.935-7.517) | 6.316(4.159-9.59) | 90.09851 | 88.64573 |
| Male | Ecuador | 3.978(3.757-4.211) | 4.441(3.725-5.294) | 4.958(3.684-6.672) | 5.535(3.642-8.412) | 6.18(3.601-10.606) | 187.8838 | 60.53933 |
| Male | Peru | 3.761(3.524-4.013) | 3.82(3.126-4.668) | 3.881(2.766-5.444) | 3.942(2.446-6.353) | 4.004(2.163-7.413) | 138.096 | 6.660972 |
| Female | Bolivia (Plurinational State of) | 4.019(3.776-4.278) | 4.723(3.895-5.726) | 5.549(4.007-7.684) | 6.52(4.121-10.316) | 7.661(4.238-13.85) | 126.8958 | 98.74443 |
| Female | Ecuador | 4.408(4.083-4.758) | 5.129(4.049-6.497) | 5.969(4.003-8.899) | 6.946(3.956-12.195) | 8.083(3.909-16.713) | 222.0089 | 90.84492 |
| Female | Peru | 4.224(3.905-4.569) | 4.425(3.472-5.639) | 4.635(3.077-6.982) | 4.856(2.726-8.649) | 5.087(2.415-10.716) | 210.3693 | 21.64693 |


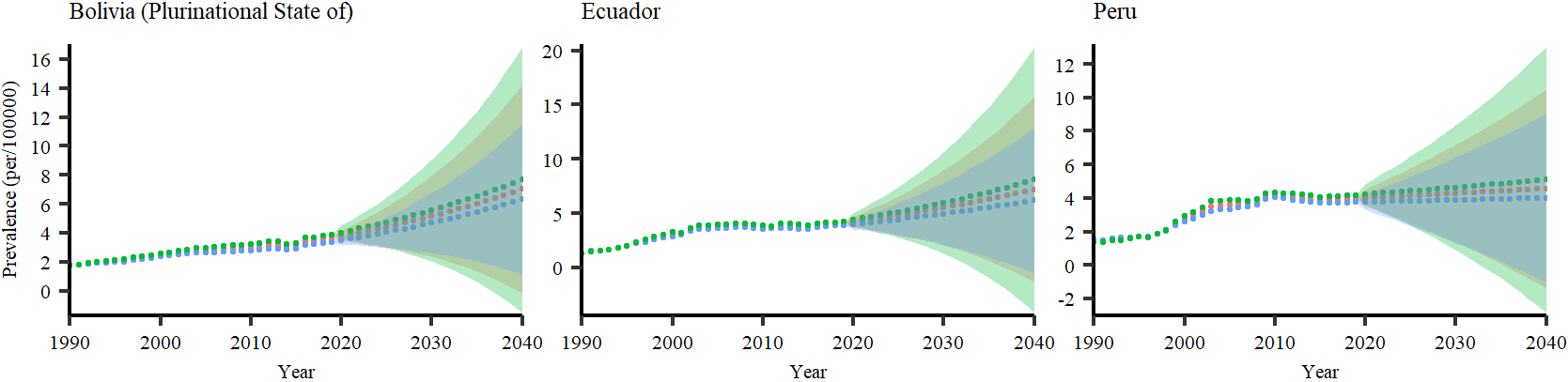


Supplemental Figure 2. Observed and projected age-standardized prevalence rate (ASPR) values from 1990 to 2040 for both sex (Red lines), females (Green lines), and men (Blue lines) in the Andean Latin America. The halo effect observed in each scatter plot accurately represents projections that extend across the temporal span from 2019 to 2040 with 95% confidence intervals.


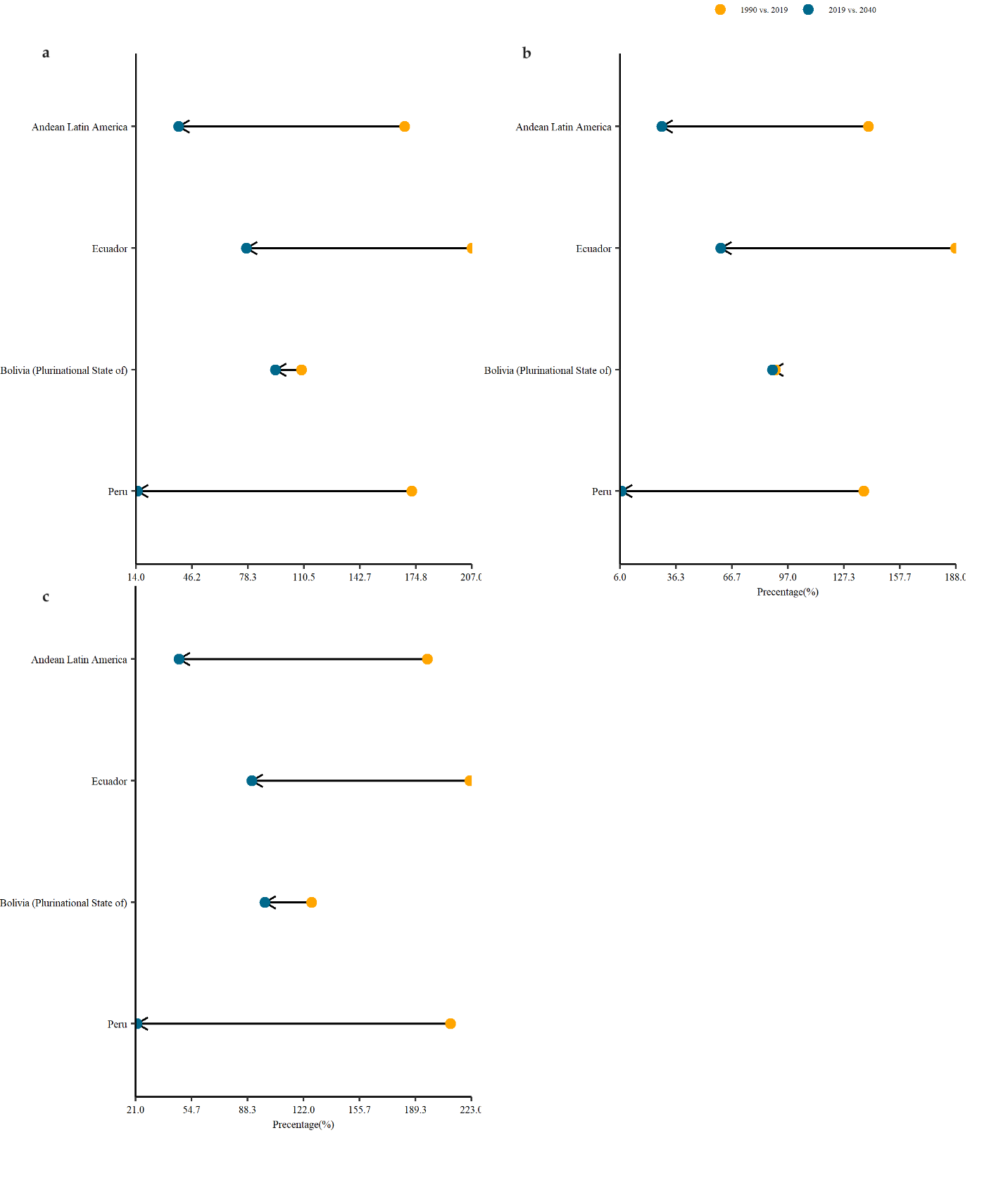


Supplemental Figure 3. The Lollipop plot between the two calculated percentage changes from 1990 to 2019 and 2019 to 2040 for both sexes (a), males (b), and females (c) in the Andean Latin America. Each line represents two time periods and show the change of ASPR increase or decrease during time.

# Australasia

| Supplemental Table 2: Age-standardized prevalence rates (ASPR) from 2020 to 2040, and percentage changes for the time periods 1990 to 2019 and 2019 to 2040, for Australasia. | | | | | | | | |
| --- | --- | --- | --- | --- | --- | --- | --- | --- |
| Group | Country | 2020 | 2025 | 2030 | 2035 | 2040 | 1990 vs. 2019 | 2019 vs. 2040 |
| Both | Australia | 8.382(8.118-8.654) | 8.577(7.771-9.468) | 8.777(7.428-10.371) | 8.982(7.099-11.363) | 9.191(6.784-12.451) | 24.51436 | 9.649666 |
| Both | New Zealand | 8.081(7.806-8.366) | 8.277(7.438-9.21) | 8.477(7.076-10.155) | 8.682(6.731-11.199) | 8.892(6.402-12.35) | 21.25889 | 10.69768 |
| Male | Australia | 9.257(8.894-9.635) | 9.361(8.273-10.593) | 9.467(7.683-11.665) | 9.573(7.132-12.848) | 9.68(6.621-14.153) | 20.75101 | 4.195963 |
| Male | New Zealand | 9.555(9.12-10.011) | 10.177(8.813-11.752) | 10.839(8.499-13.824) | 11.545(8.195-16.265) | 12.296(7.9-19.139) | 13.85054 | 30.81526 |
| Female | Australia | 7.54(7.283-7.806) | 7.839(7.043-8.726) | 8.15(6.801-9.768) | 8.474(6.566-10.937) | 8.81(6.338-12.246) | 27.79218 | 17.38352 |
| Female | New Zealand | 6.804(6.557-7.061) | 6.666(5.947-7.472) | 6.531(5.385-7.92) | 6.398(4.875-8.396) | 6.268(4.413-8.902) | 29.00543 | -8.42107 |


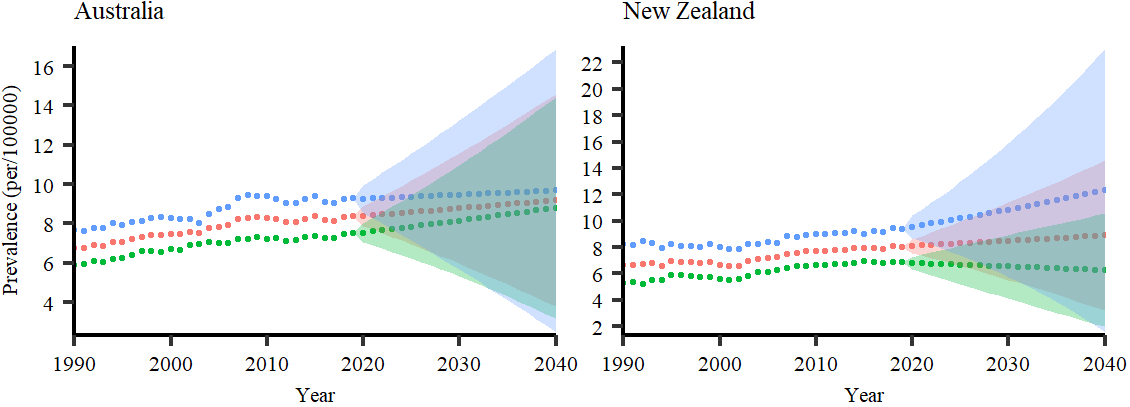


Supplemental Figure 4. Observed and projected age-standardized prevalence rate (ASPR) values from 1990 to 2040 for both sex (Red lines), females (Green lines), and men (Blue lines) in the Australasia. The halo effect observed in each scatter plot accurately represents projections that extend across the temporal span from 2019 to 2040 with 95% confidence intervals.


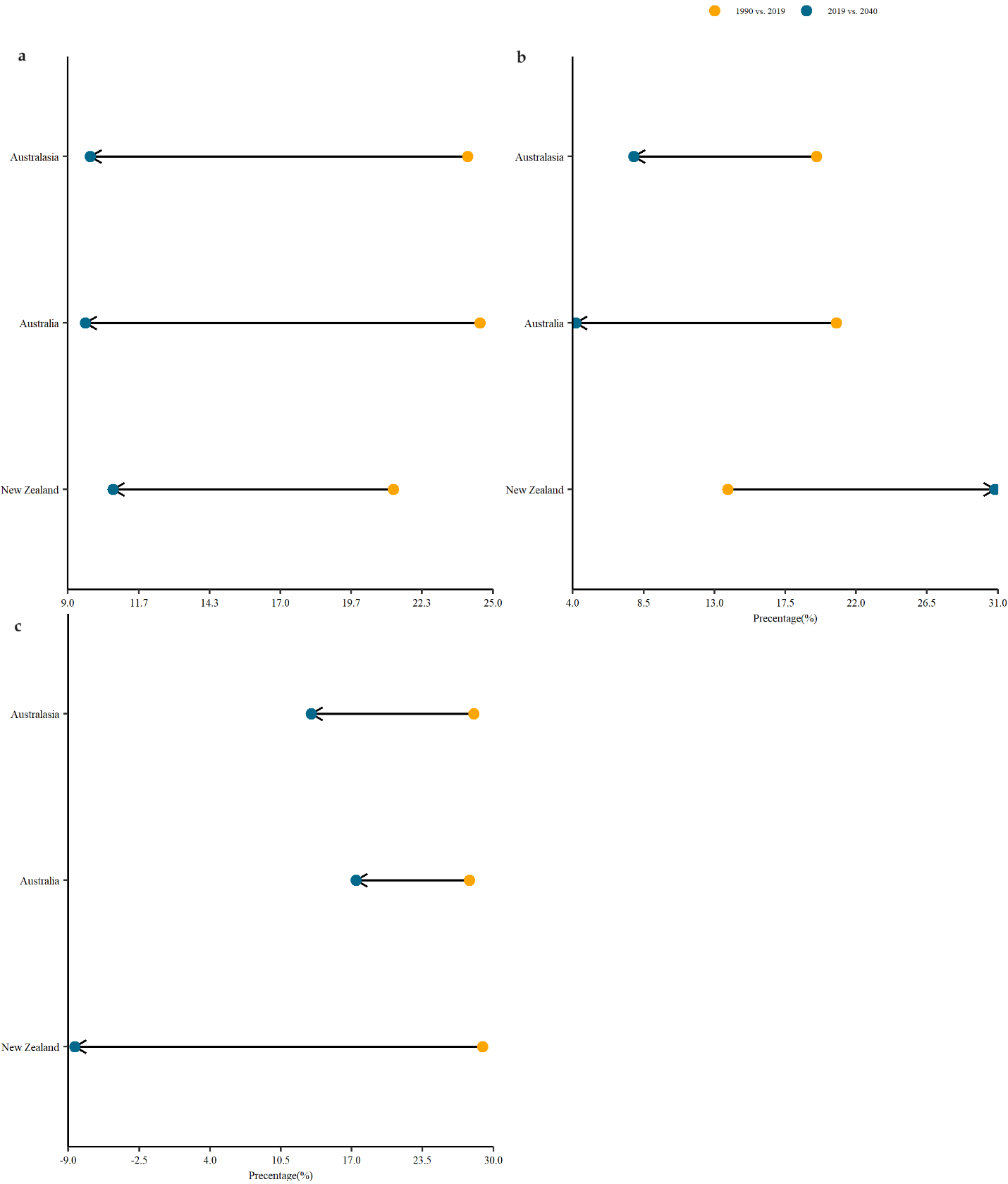


Supplemental Figure 5. The Lollipop plot between the two calculated percentage changes from 1990 to 2019 and 2019 to 2040 for both sexes (a), males (b), and females (c) in the Australasia. Each line represents two time periods and show the change of ASPR increase or decrease during time.

# Caribbean

| Supplemental Table 3: Age-standardized prevalence rates (ASPR) from 2020 to 2040, and percentage changes for the time periods 1990 to 2019 and 2019 to 2040, for Caribbean. | | | | | | | | |
| --- | --- | --- | --- | --- | --- | --- | --- | --- |
| Group | Country | 2020 | 2025 | 2030 | 2035 | 2040 | 1990 vs. 2019 | 2019 vs. 2040 |
| Both | Antigua and Barbuda | 4.65(4.406-4.907) | 4.967(4.205-5.866) | 5.305(4.005-7.027) | 5.667(3.813-8.422) | 6.053(3.63-10.094) | 336.5928 | 32.08425 |
| Both | Bahamas | 3.546(3.425-3.67) | 3.69(3.316-4.106) | 3.84(3.206-4.6) | 3.996(3.099-5.154) | 4.159(2.995-5.775) | 154.5097 | 18.26769 |
| Both | Barbados | 6.082(5.795-6.383) | 6.862(5.911-7.965) | 7.742(6.017-9.96) | 8.735(6.124-12.458) | 9.855(6.232-15.585) | 452.0547 | 65.41054 |
| Both | Belize | 4.783(4.567-5.009) | 5.248(4.551-6.051) | 5.758(4.526-7.326) | 6.318(4.5-8.871) | 6.932(4.473-10.743) | 304.0149 | 48.07835 |
| Both | Bermuda | 7.492(7.143-7.858) | 7.629(6.584-8.84) | 7.769(6.057-9.964) | 7.911(5.57-11.234) | 8.055(5.122-12.668) | 255.2794 | 7.847501 |
| Both | Cuba | 4.918(4.684-5.164) | 5.271(4.534-6.128) | 5.65(4.381-7.288) | 6.057(4.231-8.669) | 6.492(4.087-10.314) | 272.3589 | 35.57435 |
| Both | Dominica | 6.036(5.93-6.143) | 6.129(5.803-6.473) | 6.224(5.675-6.825) | 6.32(5.55-7.197) | 6.417(5.426-7.589) | 347.123 | 6.716512 |
| Both | Dominican Republic | 2.682(2.635-2.73) | 2.437(2.309-2.573) | 2.214(2.021-2.426) | 2.012(1.769-2.289) | 1.828(1.548-2.159) | 161.3716 | -33.2036 |
| Both | Grenada | 7.095(6.737-7.473) | 7.398(6.304-8.68) | 7.713(5.886-10.106) | 8.041(5.495-11.769) | 8.384(5.128-13.706) | 453.3143 | 19.3629 |
| Both | Guyana | 4.538(4.314-4.774) | 4.942(4.226-5.778) | 5.382(4.132-7.01) | 5.861(4.038-8.506) | 6.382(3.946-10.322) | 251.3026 | 43.28674 |
| Both | Haiti | 1.79(1.748-1.832) | 1.916(1.783-2.06) | 2.052(1.817-2.318) | 2.198(1.851-2.609) | 2.353(1.885-2.937) | 102.7974 | 33.47518 |
| Both | Jamaica | 3.725(3.345-4.148) | 3.541(2.539-4.937) | 3.366(1.919-5.903) | 3.2(1.45-7.062) | 3.042(1.095-8.451) | 278.4578 | -20.2281 |
| Both | Puerto Rico | 4.839(4.43-5.286) | 4.879(3.714-6.408) | 4.919(3.103-7.797) | 4.959(2.591-9.492) | 4.999(2.163-11.557) | 199.3631 | 4.047507 |
| Both | Saint Lucia | 5.693(5.527-5.864) | 6.139(5.603-6.728) | 6.621(5.673-7.728) | 7.14(5.742-8.879) | 7.7(5.813-10.201) | 305.6902 | 37.76598 |
| Both | Saint Vincent and the Grenadines | 4.711(4.508-4.923) | 5.048(4.406-5.783) | 5.409(4.298-6.807) | 5.796(4.193-8.013) | 6.211(4.089-9.434) | 283.312 | 33.67734 |
| Both | Suriname | 5.289(4.981-5.617) | 5.375(4.464-6.471) | 5.462(3.991-7.474) | 5.55(3.566-8.636) | 5.639(3.187-9.979) | 335.2985 | 6.667153 |
| Both | Trinidad and Tobago | 4.232(3.841-4.663) | 4.215(3.125-5.686) | 4.199(2.532-6.963) | 4.182(2.05-8.53) | 4.166(1.66-10.453) | 318.1611 | -2.15467 |
| Both | United States Virgin Islands | 8.196(7.908-8.494) | 7.759(6.948-8.666) | 7.346(6.095-8.854) | 6.955(5.346-9.048) | 6.585(4.689-9.247) | 195.3405 | -20.7531 |
| Male | Antigua and Barbuda | 5.434(4.969-5.943) | 5.896(4.472-7.773) | 6.396(4.009-10.204) | 6.939(3.593-13.402) | 7.528(3.219-17.605) | 313.7582 | 40.98889 |
| Male | Bahamas | 4.418(4.248-4.596) | 4.666(4.132-5.269) | 4.927(4.012-6.05) | 5.203(3.896-6.949) | 5.494(3.782-7.983) | 166.066 | 25.56265 |
| Male | Barbados | 6.762(6.454-7.085) | 7.622(6.808-8.534) | 8.592(7.168-10.299) | 9.684(7.543-12.433) | 10.916(7.938-15.011) | 460.3332 | 61.51309 |
| Male | Belize | 4.347(4.147-4.556) | 4.689(4.055-5.422) | 5.058(3.957-6.465) | 5.456(3.861-7.711) | 5.886(3.766-9.199) | 264.7327 | 37.18283 |
| Male | Bermuda | 8.941(8.497-9.409) | 9.256(7.907-10.834) | 9.581(7.342-12.502) | 9.918(6.816-14.43) | 10.266(6.327-16.658) | 320.8415 | 15.68045 |
| Male | Cuba | 5.447(5.148-5.764) | 5.927(4.977-7.057) | 6.448(4.801-8.66) | 7.015(4.629-10.631) | 7.632(4.463-13.052) | 272.4663 | 44.62151 |
| Male | Dominica | 7.254(7.122-7.389) | 7.321(6.916-7.749) | 7.389(6.712-8.134) | 7.457(6.512-8.539) | 7.526(6.319-8.964) | 372.5209 | 3.967645 |
| Male | Dominican Republic | 3.123(3.057-3.191) | 2.842(2.66-3.038) | 2.587(2.313-2.894) | 2.355(2.01-2.758) | 2.143(1.748-2.628) | 192.4694 | -32.6984 |
| Male | Grenada | 8.449(7.839-9.106) | 8.321(6.603-10.486) | 8.196(5.545-12.114) | 8.072(4.654-14.001) | 7.95(3.905-16.184) | 486.2817 | -6.29072 |
| Male | Guyana | 4.378(4.176-4.59) | 4.704(4.066-5.442) | 5.054(3.95-6.467) | 5.431(3.838-7.686) | 5.835(3.727-9.135) | 213.4756 | 35.44564 |
| Male | Haiti | 1.755(1.712-1.799) | 1.893(1.753-2.043) | 2.041(1.794-2.322) | 2.201(1.835-2.64) | 2.373(1.876-3.001) | 89.07829 | 37.50292 |
| Male | Jamaica | 4.045(3.415-4.792) | 3.453(2.047-5.826) | 2.948(1.218-7.135) | 2.516(0.724-8.745) | 2.148(0.43-10.723) | 291.435 | -49.4238 |
| Male | Puerto Rico | 5.548(5.012-6.141) | 5.214(3.81-7.134) | 4.9(2.884-8.323) | 4.605(2.182-9.717) | 4.327(1.651-11.345) | 210.6917 | -22.7124 |
| Male | Saint Lucia | 6.117(5.879-6.364) | 6.415(5.676-7.25) | 6.728(5.471-8.274) | 7.056(5.272-9.444) | 7.401(5.08-10.781) | 305.3607 | 22.15842 |
| Male | Saint Vincent and the Grenadines | 5.114(4.858-5.383) | 5.532(4.722-6.481) | 5.985(4.58-7.82) | 6.474(4.441-9.438) | 7.004(4.306-11.393) | 299.2182 | 39.1657 |
| Male | Suriname | 6.257(5.796-6.756) | 6.221(4.91-7.881) | 6.185(4.147-9.224) | 6.148(3.5-10.8) | 6.112(2.954-12.647) | 371.1192 | -2.73838 |
| Male | Trinidad and Tobago | 4.749(4.247-5.31) | 4.757(3.37-6.714) | 4.765(2.661-8.531) | 4.773(2.1-10.845) | 4.781(1.657-13.791) | 317.6121 | 0.119577 |
| Male | United States Virgin Islands | 10.994(10.402-11.62) | 10.337(8.713-12.264) | 9.72(7.281-12.974) | 9.139(6.083-13.73) | 8.593(5.081-14.531) | 252.5183 | -23.0401 |
| Female | Antigua and Barbuda | 3.947(3.832-4.064) | 4.102(3.746-4.492) | 4.264(3.657-4.97) | 4.431(3.57-5.5) | 4.606(3.485-6.087) | 355.6914 | 17.66095 |
| Female | Bahamas | 2.792(2.678-2.911) | 2.859(2.514-3.251) | 2.927(2.356-3.637) | 2.997(2.207-4.07) | 3.068(2.067-4.554) | 136.4158 | 10.59312 |
| Female | Barbados | 5.271(4.987-5.572) | 5.361(4.516-6.363) | 5.451(4.08-7.283) | 5.544(3.685-8.339) | 5.637(3.328-9.549) | 438.5183 | 7.221511 |
| Female | Belize | 5.24(4.976-5.519) | 5.817(4.958-6.825) | 6.457(4.929-8.459) | 7.168(4.899-10.488) | 7.956(4.868-13.004) | 350.8912 | 56.14353 |
| Female | Bermuda | 6.146(5.859-6.448) | 6.104(5.265-7.077) | 6.062(4.721-7.783) | 6.02(4.233-8.561) | 5.978(3.794-9.418) | 200.9057 | -3.07318 |
| Female | Cuba | 4.411(4.207-4.624) | 4.648(4.017-5.377) | 4.897(3.828-6.265) | 5.16(3.647-7.302) | 5.438(3.474-8.511) | 273.0738 | 25.89837 |
| Female | Dominica | 4.759(4.652-4.869) | 4.875(4.544-5.23) | 4.993(4.433-5.624) | 5.115(4.326-6.048) | 5.239(4.22-6.504) | 291.8028 | 10.72711 |
| Female | Dominican Republic | 2.254(2.202-2.306) | 2.048(1.907-2.2) | 1.861(1.649-2.1) | 1.691(1.427-2.005) | 1.537(1.234-1.915) | 128.6754 | -33.1899 |
| Female | Grenada | 5.697(5.403-6.007) | 6.129(5.204-7.218) | 6.594(5.002-8.693) | 7.094(4.806-10.472) | 7.632(4.617-12.617) | 396.6437 | 36.16708 |
| Female | Guyana | 4.629(4.367-4.908) | 5.09(4.25-6.095) | 5.596(4.126-7.589) | 6.153(4.005-9.452) | 6.764(3.887-11.773) | 288.6159 | 49.11421 |
| Female | Haiti | 1.82(1.778-1.863) | 1.937(1.803-2.081) | 2.061(1.826-2.326) | 2.193(1.849-2.601) | 2.334(1.873-2.908) | 117.1888 | 29.95204 |
| Female | Jamaica | 3.41(3.208-3.625) | 3.696(3.061-4.463) | 4.006(2.914-5.509) | 4.343(2.772-6.802) | 4.707(2.637-8.401) | 260.7033 | 39.12045 |
| Female | Puerto Rico | 4.226(3.89-4.592) | 4.616(3.574-5.963) | 5.042(3.271-7.771) | 5.507(2.993-10.132) | 6.015(2.738-13.212) | 188.1722 | 46.06825 |
| Female | Saint Lucia | 5.243(4.995-5.505) | 5.822(5.01-6.764) | 6.464(5.016-8.33) | 7.176(5.02-10.26) | 7.968(5.023-12.639) | 297.2825 | 56.22606 |
| Female | Saint Vincent and the Grenadines | 4.264(4.072-4.465) | 4.535(3.933-5.23) | 4.823(3.791-6.136) | 5.13(3.654-7.202) | 5.456(3.521-8.454) | 262.3949 | 29.52284 |
| Female | Suriname | 4.4(4.213-4.594) | 4.588(4.013-5.244) | 4.784(3.816-5.997) | 4.988(3.628-6.859) | 5.201(3.448-7.845) | 296.2277 | 18.92886 |
| Female | Trinidad and Tobago | 3.724(3.402-4.076) | 3.69(2.792-4.877) | 3.656(2.282-5.857) | 3.622(1.864-7.037) | 3.589(1.523-8.457) | 313.7204 | -4.24443 |
| Female | United States Virgin Islands | 5.813(5.722-5.906) | 5.626(5.358-5.907) | 5.444(5.013-5.912) | 5.268(4.69-5.918) | 5.098(4.388-5.924) | 136.0209 | -13.0716 |


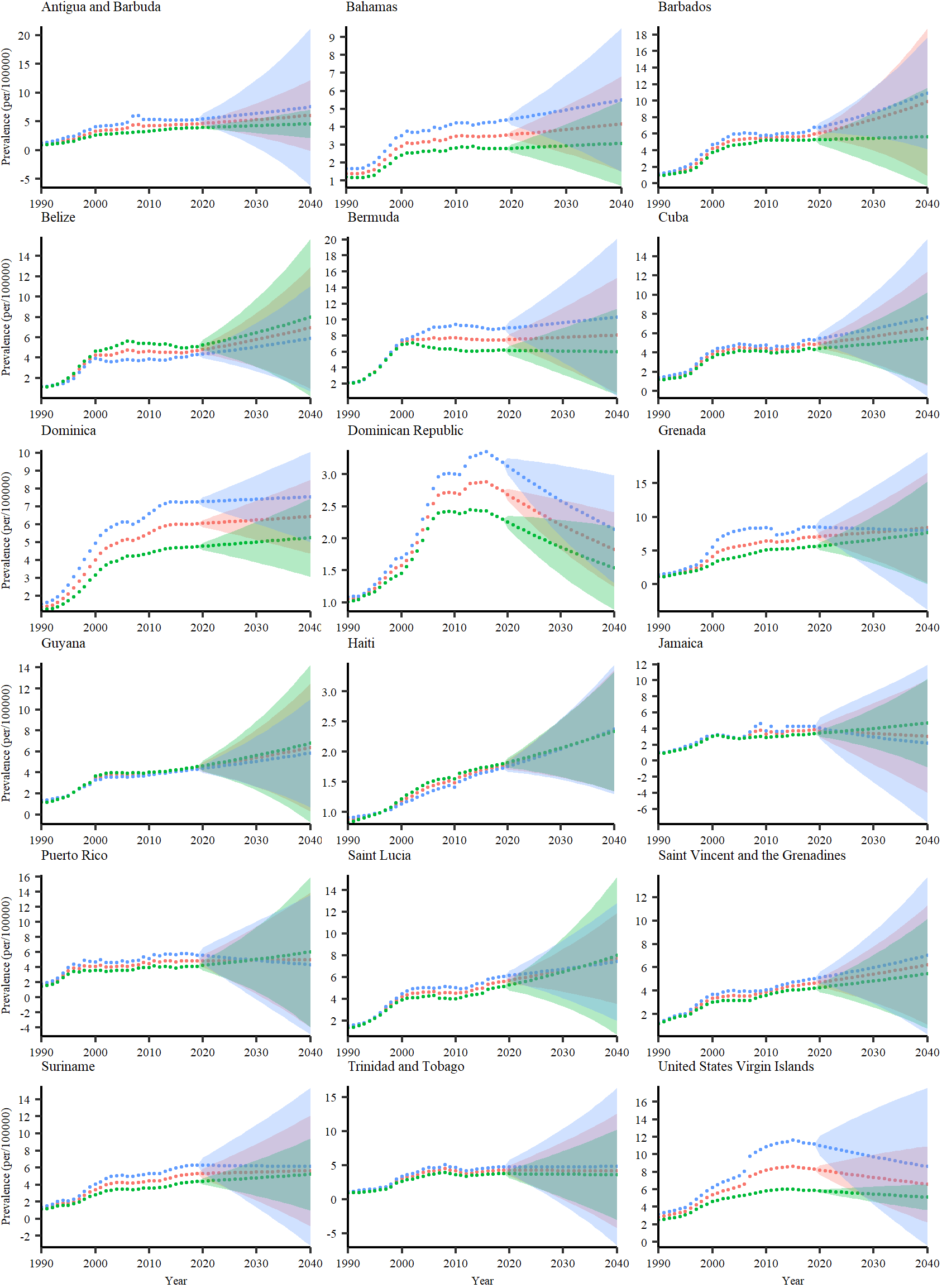


Supplemental Figure 6. Observed and projected age-standardized prevalence rate (ASPR) values from 1990 to 2040 for both sex (Red lines), females (Green lines), and men (Blue lines) in the Caribbean. The halo effect observed in each scatter plot accurately represents projections that extend across the temporal span from 2019 to 2040 with 95% confidence intervals.


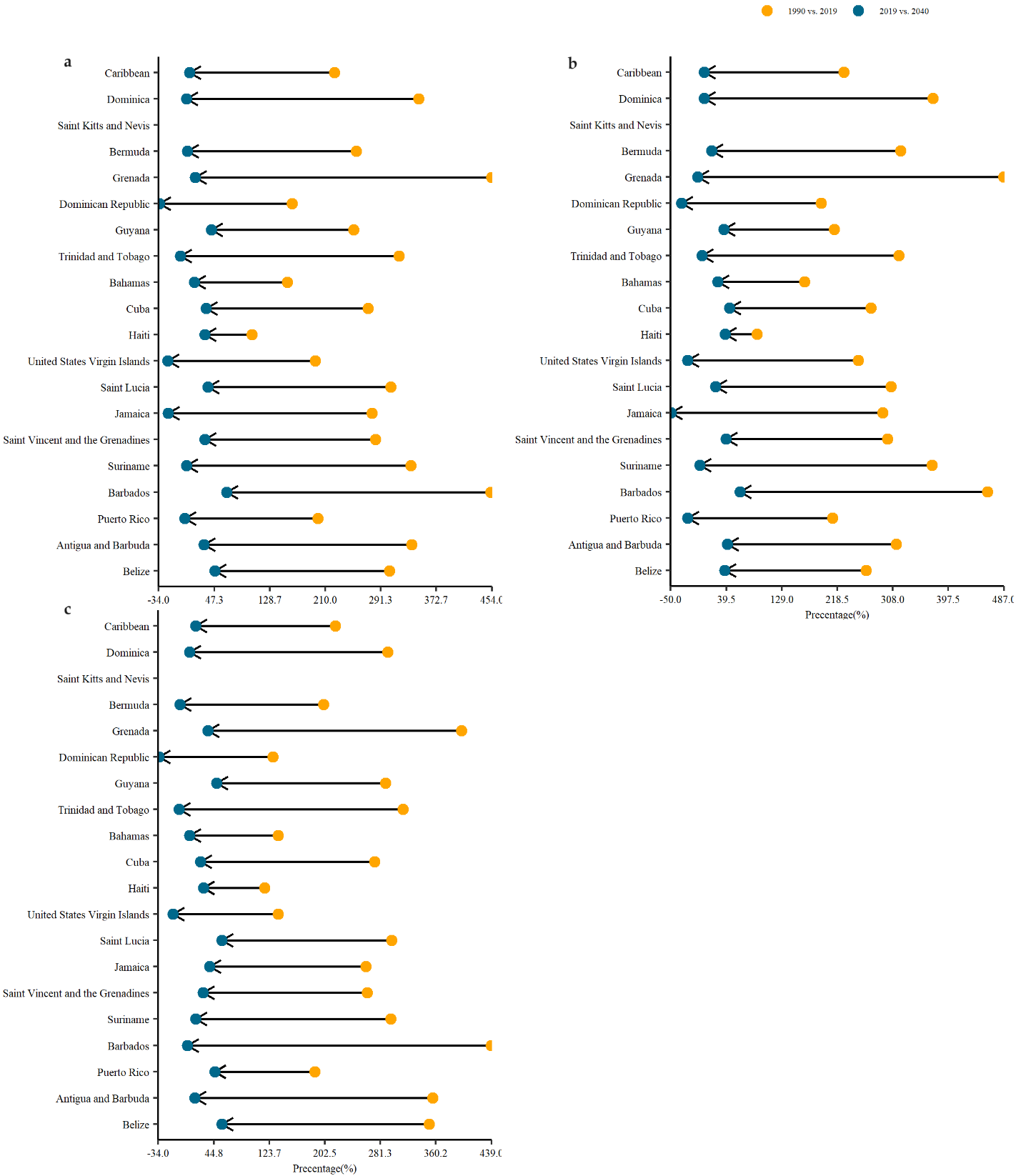


Supplemental Figure 7. The Lollipop plot between the two calculated percentage changes from 1990 to 2019 and 2019 to 2040 for both sexes (a), males (b), and females (c) in the Caribbean. Each line represents two time periods and show the change of ASPR increase or decrease during time.

# Central Asia

| Supplemental Table 4: Age-standardized prevalence rates (ASPR) from 2020 to 2040, and percentage changes for the time periods 1990 to 2019 and 2019 to 2040, for Central Asia. | | | | | | | | |
| --- | --- | --- | --- | --- | --- | --- | --- | --- |
| Group | Country | 2020 | 2025 | 2030 | 2035 | 2040 | 1990 vs. 2019 | 2019 vs. 2040 |
| Both | Armenia | 7.754(7.453-8.067) | 8.034(7.109-9.078) | 8.323(6.77-10.232) | 8.623(6.446-11.536) | 8.934(6.136-13.007) | 52.35357 | 15.6407 |
| Both | Azerbaijan | 5.332(5.188-5.479) | 5.389(4.954-5.862) | 5.447(4.725-6.279) | 5.505(4.506-6.726) | 5.565(4.297-7.206) | 100.1067 | 4.633838 |
| Both | Georgia | 4.889(4.675-5.113) | 4.948(4.31-5.682) | 5.009(3.966-6.326) | 5.07(3.648-7.045) | 5.131(3.356-7.847) | 60.1937 | 5.230734 |
| Both | Kazakhstan | 5.073(4.844-5.314) | 4.814(4.173-5.554) | 4.568(3.588-5.817) | 4.335(3.084-6.093) | 4.113(2.651-6.384) | 462.4067 | -19.2941 |
| Both | Kyrgyzstan | 3.894(3.678-4.123) | 3.804(3.188-4.539) | 3.716(2.757-5.008) | 3.63(2.384-5.527) | 3.546(2.06-6.101) | 34.8798 | -9.38551 |
| Both | Mongolia | 4.606(4.538-4.674) | 5.084(4.858-5.321) | 5.613(5.197-6.061) | 6.196(5.559-6.905) | 6.84(5.947-7.867) | 97.16635 | 51.61179 |
| Both | Tajikistan | 3.678(3.486-3.879) | 3.572(3.029-4.213) | 3.47(2.627-4.585) | 3.371(2.276-4.992) | 3.275(1.973-5.435) | 48.87728 | -11.4026 |
| Both | Turkmenistan | 2.889(2.734-3.053) | 3.137(2.647-3.717) | 3.406(2.556-4.538) | 3.698(2.468-5.54) | 4.014(2.382-6.766) | 178.6311 | 41.08483 |
| Both | Uzbekistan | 3.497(3.386-3.612) | 3.376(3.056-3.728) | 3.258(2.754-3.854) | 3.145(2.482-3.985) | 3.036(2.236-4.121) | 210.2792 | -13.6883 |
| Male | Armenia | 10.117(9.677-10.577) | 10.597(9.237-12.157) | 11.1(8.801-13.999) | 11.627(8.383-16.125) | 12.178(7.985-18.574) | 57.33766 | 21.23207 |
| Male | Azerbaijan | 6.675(6.536-6.818) | 6.715(6.292-7.167) | 6.755(6.051-7.54) | 6.794(5.819-7.934) | 6.835(5.595-8.349) | 88.00463 | 2.5854 |
| Male | Georgia | 6.737(6.363-7.133) | 6.786(5.69-8.093) | 6.835(5.075-9.206) | 6.885(4.525-10.474) | 6.934(4.035-11.918) | 71.75188 | 3.231644 |
| Male | Kazakhstan | 6.378(6.073-6.698) | 5.751(4.944-6.69) | 5.186(4.016-6.696) | 4.676(3.261-6.704) | 4.216(2.648-6.712) | 542.9565 | -34.6032 |
| Male | Kyrgyzstan | 4.699(4.4-5.018) | 4.52(3.689-5.537) | 4.348(3.085-6.127) | 4.182(2.579-6.783) | 4.023(2.155-7.509) | 24.21319 | -15.0771 |
| Male | Mongolia | 6.118(6.01-6.227) | 6.882(6.515-7.269) | 7.741(7.057-8.491) | 8.708(7.643-9.92) | 9.795(8.278-11.59) | 120.9231 | 63.85992 |
| Male | Tajikistan | 3.906(3.734-4.085) | 3.621(3.152-4.16) | 3.357(2.656-4.244) | 3.113(2.237-4.33) | 2.886(1.884-4.419) | 34.50572 | -27.2833 |
| Male | Turkmenistan | 3.328(3.098-3.576) | 3.478(2.787-4.34) | 3.634(2.5-5.283) | 3.797(2.241-6.434) | 3.968(2.009-7.837) | 199.4129 | 19.84758 |
| Male | Uzbekistan | 3.806(3.657-3.962) | 3.664(3.237-4.146) | 3.527(2.861-4.347) | 3.395(2.529-4.558) | 3.268(2.234-4.78) | 212.5284 | -14.7363 |
| Female | Armenia | 5.842(5.573-6.123) | 5.966(5.159-6.899) | 6.093(4.766-7.788) | 6.222(4.402-8.794) | 6.354(4.065-9.932) | 46.12225 | 8.685706 |
| Female | Azerbaijan | 4.155(3.986-4.331) | 4.205(3.7-4.78) | 4.256(3.428-5.285) | 4.308(3.175-5.845) | 4.36(2.94-6.465) | 111.7602 | 5.162717 |
| Female | Georgia | 3.411(3.23-3.602) | 3.461(2.925-4.095) | 3.512(2.643-4.666) | 3.563(2.387-5.319) | 3.616(2.156-6.064) | 42.29921 | 6.10811 |
| Female | Kazakhstan | 4.126(3.946-4.313) | 4.11(3.582-4.716) | 4.095(3.246-5.166) | 4.08(2.941-5.66) | 4.065(2.664-6.202) | 384.77 | -1.29463 |
| Female | Kyrgyzstan | 3.232(3.068-3.405) | 3.157(2.688-3.708) | 3.084(2.35-4.047) | 3.012(2.053-4.419) | 2.943(1.794-4.825) | 44.90722 | -9.45697 |
| Female | Mongolia | 3.455(3.388-3.524) | 3.78(3.558-4.016) | 4.136(3.733-4.582) | 4.525(3.917-5.227) | 4.95(4.109-5.963) | 73.06884 | 46.17912 |
| Female | Tajikistan | 3.491(3.268-3.729) | 3.562(2.904-4.368) | 3.634(2.574-5.13) | 3.707(2.28-6.027) | 3.782(2.02-7.082) | 62.56175 | 9.048353 |
| Female | Turkmenistan | 2.537(2.412-2.668) | 2.856(2.444-3.338) | 3.216(2.471-4.184) | 3.621(2.498-5.247) | 4.077(2.526-6.581) | 158.171 | 64.82471 |
| Female | Uzbekistan | 3.213(3.129-3.3) | 3.098(2.854-3.363) | 2.988(2.6-3.432) | 2.881(2.369-3.503) | 2.778(2.158-3.575) | 205.9349 | -14.0299 |


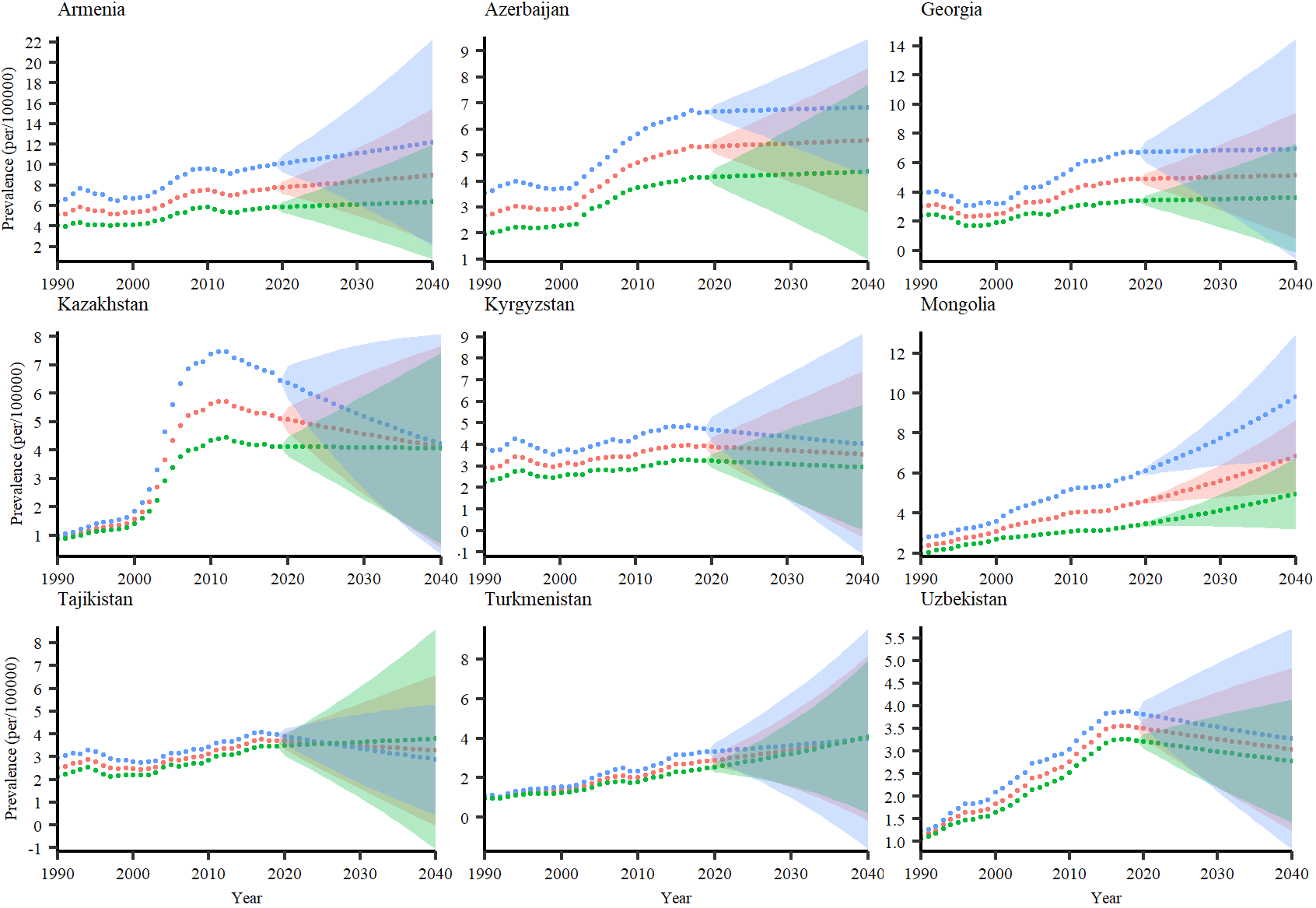


Supplemental Figure 8. Observed and projected age-standardized prevalence rate (ASPR) values from 1990 to 2040 for both sex (Red lines), females (Green lines), and men (Blue lines) in the Central Asia. The halo effect observed in each scatter plot accurately represents projections that extend across the temporal span from 2019 to 2040 with 95% confidence intervals.


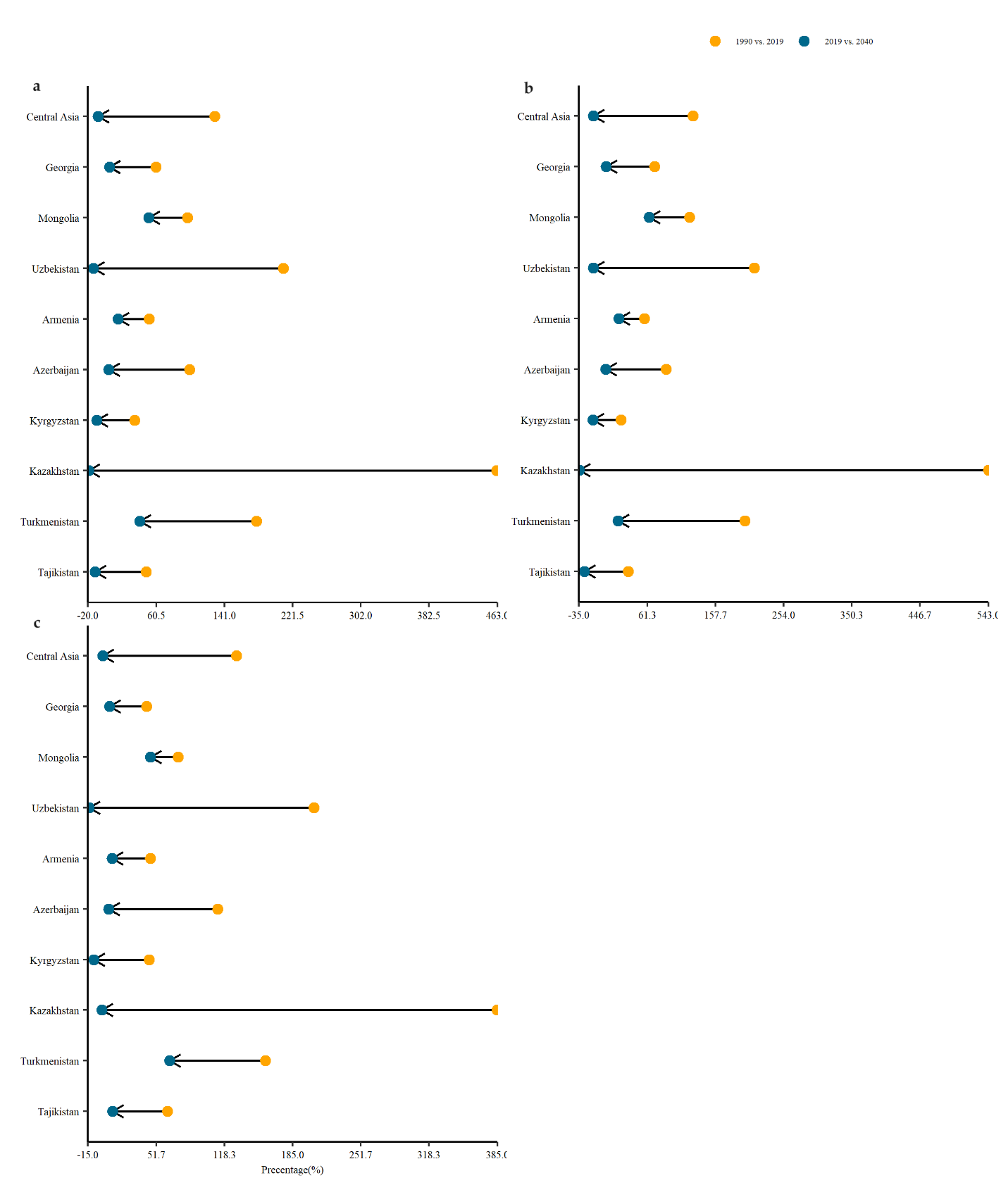


Supplemental Figure 9. The Lollipop plot between the two calculated percentage changes from 1990 to 2019 and 2019 to 2040 for both sexes (a), males (b), and females (c) in the Central Asia. Each line represents two time periods and show the change of ASPR increase or decrease during time.
