# Supplementary material for: Global Trend in Pancreatic Cancer Prevalence Rates Through 2040: An Illness‐Death Modeling Study
Source: Cancer Med. 2024 Oct 23;13(20):e70318. doi: 10.1002/cam4.70318 (PMC11497012; doi:10.1002/cam4.70318)
Supplement: Supplementary file 2 — Data S2. [file CAM4-13-e70318-s002.docx]

# Central Europe

| Supplemental Table 5: Age-standardized prevalence rates (ASPR) from 2020 to 2040, and percentage changes for the time periods 1990 to 2019 and 2019 to 2040, for Central Europe. | | | | | | | | |
| --- | --- | --- | --- | --- | --- | --- | --- | --- |
| Group | Country | 2020 | 2025 | 2030 | 2035 | 2040 | 1990 vs. 2019 | 2019 vs. 2040 |
| Both | Albania | 5.146(4.921-5.381) | 5.289(4.607-6.071) | 5.436(4.306-6.862) | 5.587(4.023-7.759) | 5.742(3.758-8.773) | 82.22299 | 11.79027 |
| Both | Bosnia and Herzegovina | 7.574(7.349-7.807) | 7.954(7.244-8.733) | 8.352(7.132-9.781) | 8.77(7.02-10.956) | 9.209(6.91-12.274) | 31.23063 | 22.566 |
| Both | Bulgaria | 8.649(8.34-8.969) | 8.569(7.659-9.587) | 8.49(7.023-10.263) | 8.412(6.438-10.99) | 8.334(5.902-11.768) | 65.60613 | -4.15386 |
| Both | Croatia | 8.526(7.988-9.1) | 7.837(6.41-9.583) | 7.204(5.129-10.119) | 6.622(4.102-10.689) | 6.087(3.281-11.292) | 20.45504 | -30.58 |
| Both | Czechia | 9.654(9.184-10.147) | 9.721(8.335-11.338) | 9.789(7.548-12.695) | 9.857(6.833-14.218) | 9.925(6.186-15.926) | 7.092393 | 3.141684 |
| Both | Hungary | 9.552(9.198-9.919) | 9.457(8.417-10.625) | 9.363(7.689-11.4) | 9.269(7.023-12.234) | 9.177(6.415-13.129) | 22.8294 | -3.5948 |
| Both | North Macedonia | 8.243(8.071-8.42) | 8.481(7.944-9.055) | 8.726(7.813-9.746) | 8.978(7.684-10.492) | 9.238(7.555-11.295) | 61.23187 | 12.54224 |
| Both | Montenegro | 8.519(8.35-8.692) | 8.438(7.931-8.977) | 8.358(7.528-9.281) | 8.279(7.144-9.595) | 8.201(6.779-9.921) | 47.04386 | -4.04625 |
| Both | Poland | 7.582(7.353-7.819) | 8.317(7.563-9.145) | 9.122(7.77-10.711) | 10.006(7.981-12.546) | 10.975(8.197-14.696) | 9.58718 | 47.8051 |
| Both | Romania | 7.98(7.559-8.424) | 8.314(7.034-9.828) | 8.663(6.53-11.492) | 9.025(6.06-13.442) | 9.403(5.623-15.725) | 55.01836 | 18.50714 |
| Both | Serbia | 7.649(7.371-7.938) | 7.64(6.815-8.566) | 7.631(6.29-9.257) | 7.621(5.805-10.007) | 7.612(5.356-10.818) | 57.24192 | -1.10526 |
| Both | Slovakia | 11.593(11.304-11.888) | 12.008(11.109-12.98) | 12.439(10.906-14.186) | 12.884(10.705-15.507) | 13.346(10.508-16.952) | 33.65739 | 15.70586 |
| Both | Slovenia | 7.971(7.645-8.311) | 8.083(7.106-9.195) | 8.197(6.593-10.191) | 8.312(6.115-11.298) | 8.429(5.672-12.526) | 27.12183 | 6.13827 |
| Male | Central Europe | 10.3(10.069-10.535) | 10.557(9.845-11.32) | 10.821(9.617-12.175) | 11.091(9.392-13.097) | 11.368(9.173-14.089) | 23.17136 | 10.92618 |
| Male | Albania | 6.775(6.445-7.122) | 6.793(5.823-7.926) | 6.811(5.249-8.838) | 6.829(4.731-9.859) | 6.848(4.263-10.998) | 78.84873 | 0.573909 |
| Male | Bosnia and Herzegovina | 9.146(8.818-9.486) | 9.553(8.535-10.692) | 9.978(8.248-12.071) | 10.422(7.97-13.63) | 10.886(7.7-15.391) | 28.09647 | 19.93899 |
| Male | Bulgaria | 11.503(11.078-11.944) | 11.327(10.085-12.723) | 11.154(9.166-13.574) | 10.983(8.328-14.485) | 10.815(7.567-15.458) | 63.28353 | -6.49183 |
| Male | Croatia | 10.226(9.547-10.954) | 9.729(7.868-12.03) | 9.256(6.466-13.251) | 8.807(5.312-14.6) | 8.379(4.363-16.09) | 14.24113 | -19.4162 |
| Male | Czechia | 11.351(10.709-12.032) | 11.356(9.487-13.593) | 11.361(8.384-15.394) | 11.366(7.407-17.44) | 11.37(6.543-19.76) | -4.25077 | 0.502128 |
| Male | Hungary | 11.668(11.206-12.148) | 11.146(9.841-12.624) | 10.648(8.628-13.142) | 10.173(7.562-13.684) | 9.718(6.628-14.25) | 15.67701 | -16.8495 |
| Male | North Macedonia | 10.224(9.912-10.547) | 10.455(9.5-11.506) | 10.691(9.093-12.57) | 10.933(8.703-13.734) | 11.18(8.328-15.008) | 58.35579 | 9.633365 |
| Male | Montenegro | 10.871(10.579-11.17) | 10.772(9.905-11.715) | 10.674(9.263-12.3) | 10.577(8.661-12.916) | 10.481(8.098-13.564) | 41.99242 | -3.9366 |
| Male | Poland | 9.421(9.051-9.806) | 10.241(9.05-11.59) | 11.133(9.034-13.72) | 12.103(9.015-16.247) | 13.156(8.996-19.24) | 6.913141 | 42.34711 |
| Male | Romania | 10.624(9.917-11.381) | 10.921(8.83-13.506) | 11.226(7.84-16.075) | 11.54(6.958-19.141) | 11.863(6.174-22.794) | 50.61457 | 11.97037 |
| Male | Serbia | 9.102(8.77-9.447) | 9.032(8.053-10.131) | 8.963(7.383-10.881) | 8.894(6.768-11.689) | 8.826(6.203-12.559) | 54.72372 | -3.80119 |
| Male | Slovakia | 13.954(13.542-14.378) | 14.163(12.912-15.535) | 14.375(12.296-16.806) | 14.591(11.708-18.184) | 14.81(11.146-19.677) | 19.74494 | 6.110364 |
| Male | Slovenia | 9.602(9.024-10.218) | 9.444(7.796-11.44) | 9.288(6.718-12.841) | 9.135(5.787-14.42) | 8.984(4.984-16.195) | 30.1674 | -6.6639 |
| Female | Central Europe | 6.489(6.375-6.604) | 6.753(6.394-7.132) | 7.027(6.408-7.707) | 7.313(6.422-8.329) | 7.611(6.435-9.002) | 31.75538 | 18.13836 |
| Female | Albania | 3.653(3.496-3.818) | 3.955(3.453-4.53) | 4.282(3.404-5.386) | 4.635(3.354-6.405) | 5.018(3.306-7.618) | 89.50912 | 39.4921 |
| Female | Bosnia and Herzegovina | 6.181(5.978-6.39) | 6.519(5.881-7.226) | 6.876(5.778-8.183) | 7.253(5.676-9.268) | 7.65(5.575-10.498) | 32.34966 | 24.78852 |
| Female | Bulgaria | 6.178(5.881-6.49) | 6.148(5.28-7.159) | 6.118(4.73-7.914) | 6.089(4.237-8.75) | 6.059(3.795-9.676) | 72.23014 | -2.53477 |
| Female | Croatia | 7.092(6.603-7.618) | 6.244(5.007-7.786) | 5.496(3.785-7.981) | 4.839(2.861-8.184) | 4.26(2.162-8.394) | 27.24213 | -42.3862 |
| Female | Czechia | 8.065(7.68-8.47) | 8.112(6.974-9.436) | 8.159(6.32-10.534) | 8.206(5.726-11.762) | 8.254(5.187-13.136) | 19.86545 | 2.470661 |
| Female | Hungary | 7.797(7.486-8.12) | 7.965(7.026-9.029) | 8.137(6.583-10.056) | 8.312(6.167-11.204) | 8.491(5.776-12.483) | 31.16769 | 9.803109 |
| Female | North Macedonia | 6.401(6.237-6.568) | 6.605(6.099-7.153) | 6.816(5.958-7.799) | 7.034(5.819-8.504) | 7.259(5.682-9.273) | 65.26882 | 14.01771 |
| Female | Montenegro | 6.45(6.346-6.555) | 6.389(6.077-6.716) | 6.328(5.815-6.886) | 6.268(5.564-7.061) | 6.209(5.324-7.241) | 51.96571 | -3.97598 |
| Female | Poland | 5.955(5.823-6.09) | 6.547(6.109-7.016) | 7.197(6.402-8.089) | 7.911(6.709-9.329) | 8.697(7.03-10.758) | 11.60011 | 49.15513 |
| Female | Romania | 5.656(5.474-5.843) | 5.988(5.415-6.621) | 6.34(5.349-7.513) | 6.712(5.283-8.527) | 7.106(5.218-9.678) | 61.29379 | 26.8578 |
| Female | Serbia | 6.317(6.056-6.59) | 6.322(5.548-7.203) | 6.326(5.074-7.886) | 6.33(4.639-8.637) | 6.335(4.242-9.46) | 60.91373 | -0.25975 |
| Female | Slovakia | 9.476(9.16-9.802) | 9.958(8.968-11.057) | 10.465(8.769-12.49) | 10.998(8.572-14.112) | 11.558(8.378-15.946) | 51.82698 | 23.11889 |
| Female | Slovenia | 6.53(6.192-6.886) | 6.828(5.796-8.044) | 7.14(5.413-9.419) | 7.467(5.054-11.032) | 7.808(4.718-12.922) | 20.84264 | 20.80024 |


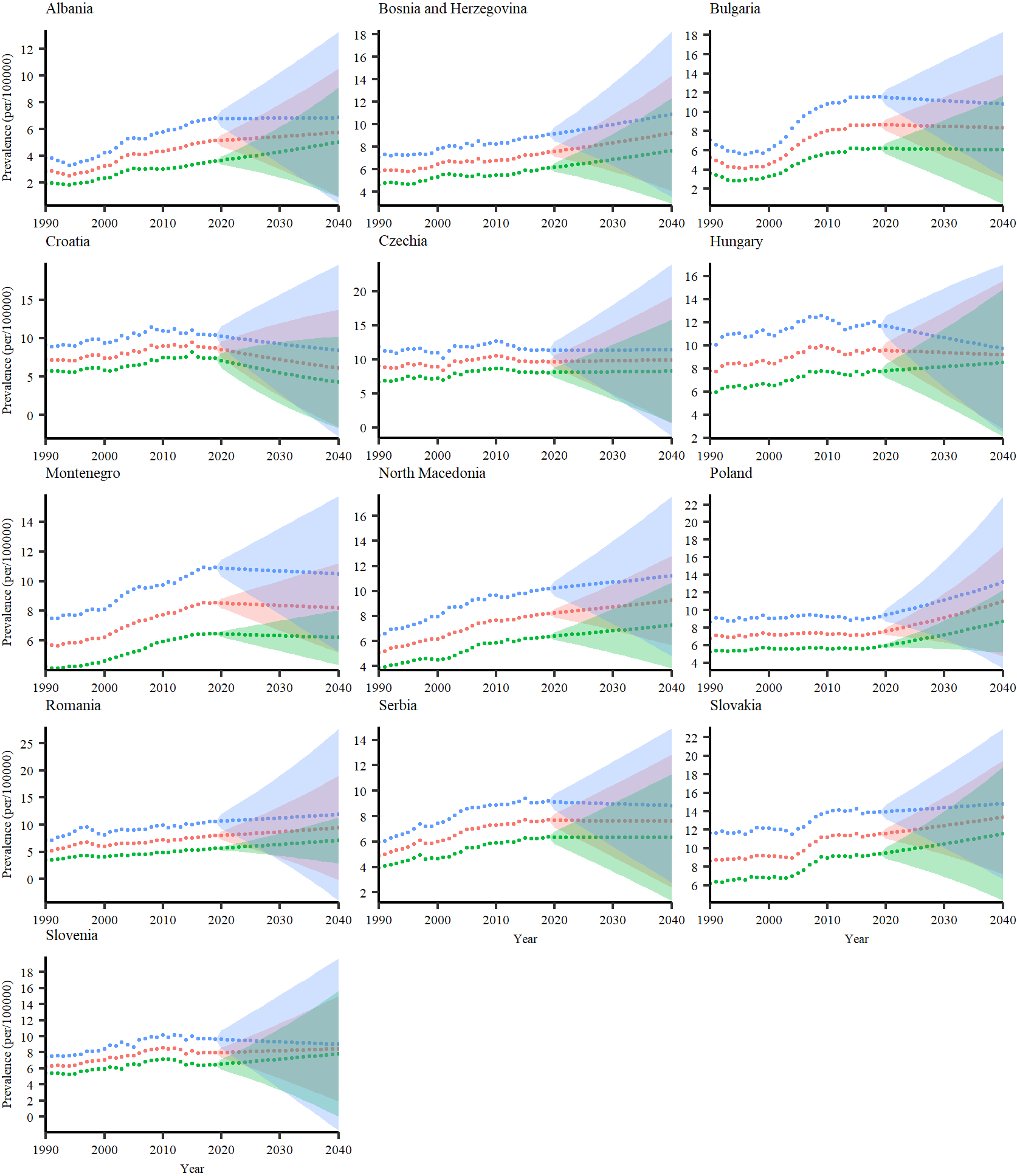


Supplemental Figure 10. Observed and projected age-standardized prevalence rate (ASPR) values from 1990 to 2040 for both sex (Red lines), females (Green lines), and men (Blue lines) in the Central Europe. The halo effect observed in each scatter plot accurately represents projections that extend across the temporal span from 2019 to 2040 with 95% confidence intervals.


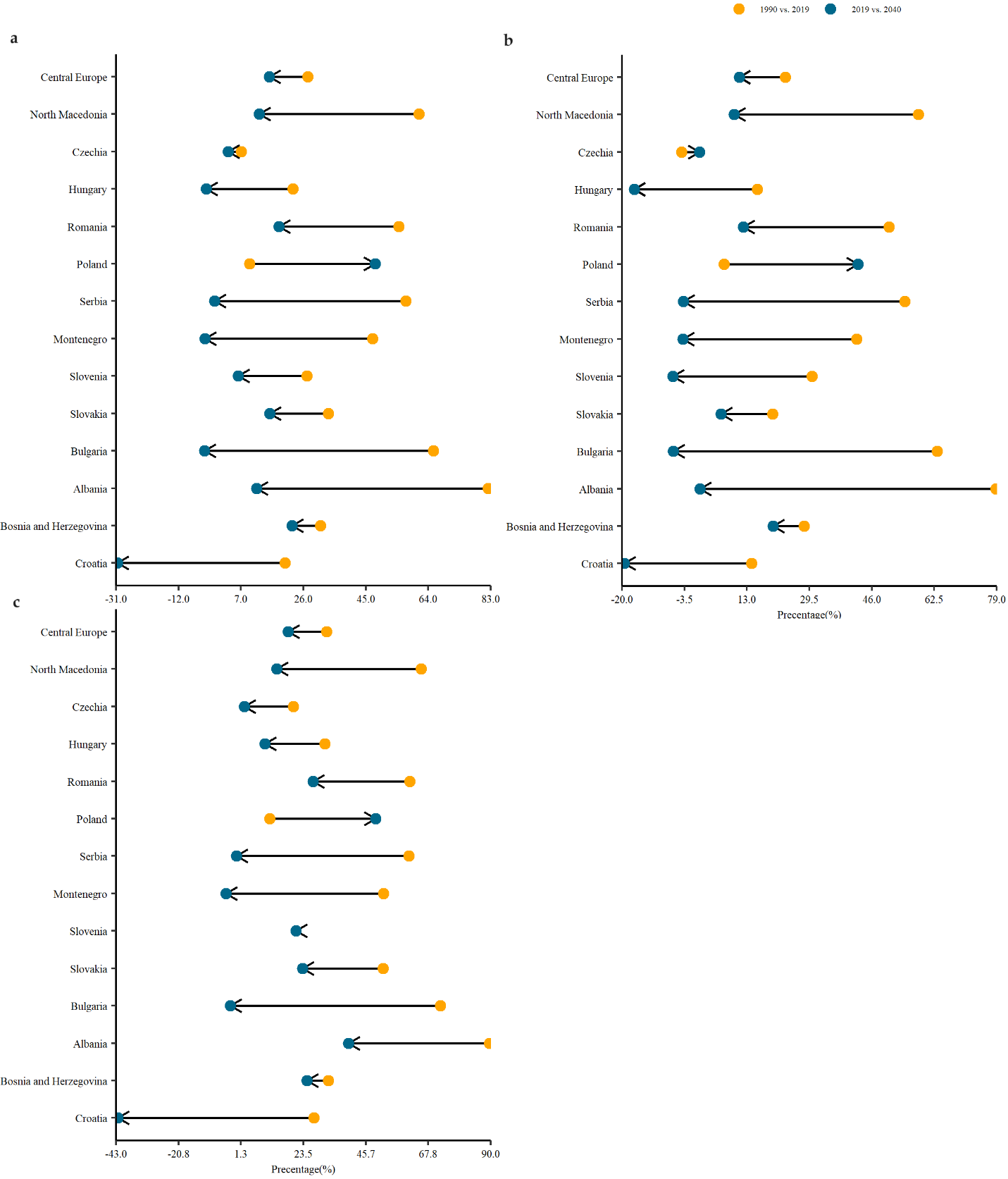


Supplemental Figure 11. The Lollipop plot between the two calculated percentage changes from 1990 to 2019 and 2019 to 2040 for both sexes (a), males (b), and females (c) in the Central Europe. Each line represents two time periods and show the change of ASPR increase or decrease during time.

# Central Latin America

| Supplemental Table 6: Age-standardized prevalence rates (ASPR) from 2020 to 2040, and percentage changes for the time periods 1990 to 2019 and 2019 to 2040, for Central Latin America. | | | | | | | | |
| --- | --- | --- | --- | --- | --- | --- | --- | --- |
| Group | Country | 2020 | 2025 | 2030 | 2035 | 2040 | 1990 vs. 2019 | 2019 vs. 2040 |
| Both | Colombia | 3.592(3.423-3.77) | 3.788(3.263-4.397) | 3.994(3.104-5.139) | 4.211(2.952-6.008) | 4.44(2.807-7.024) | -8.93279 | 24.60052 |
| Both | Costa Rica | 5.434(5.039-5.86) | 5.614(4.446-7.089) | 5.801(3.911-8.604) | 5.994(3.439-10.446) | 6.193(3.024-12.684) | 130.5606 | 14.70606 |
| Both | El Salvador | 3.835(3.621-4.061) | 3.859(3.234-4.604) | 3.883(2.881-5.234) | 3.907(2.565-5.951) | 3.932(2.284-6.768) | 207.2276 | 1.852699 |
| Both | Guatemala | 3.344(3.145-3.554) | 3.434(2.844-4.147) | 3.527(2.564-4.85) | 3.622(2.311-5.675) | 3.72(2.083-6.641) | 203.6618 | 11.6378 |
| Both | Honduras | 3.184(3.07-3.303) | 3.53(3.153-3.952) | 3.913(3.233-4.736) | 4.337(3.314-5.676) | 4.807(3.397-6.803) | 116.4923 | 54.66871 |
| Both | Mexico | 4.62(4.453-4.793) | 5.454(4.87-6.109) | 6.439(5.317-7.798) | 7.602(5.804-9.957) | 8.975(6.335-12.715) | 11.13636 | 101.0808 |
| Both | Nicaragua | 4.14(3.909-4.385) | 4.465(3.738-5.333) | 4.816(3.567-6.502) | 5.193(3.402-7.928) | 5.601(3.244-9.67) | 139.6174 | 37.35496 |
| Both | Panama | 3.69(3.53-3.858) | 4.065(3.544-4.664) | 4.478(3.551-5.648) | 4.934(3.557-6.842) | 5.435(3.564-8.289) | 99.71287 | 50.52408 |
| Both | Venezuela (Bolivarian Republic of) | 4.459(4.173-4.764) | 4.973(4.053-6.101) | 5.546(3.926-7.834) | 6.185(3.801-10.064) | 6.898(3.68-12.929) | 288.7744 | 57.20451 |
| Male | Colombia | 3.447(3.144-3.779) | 3.627(2.731-4.817) | 3.816(2.362-6.165) | 4.015(2.043-7.894) | 4.225(1.766-10.109) | -8.00595 | 23.59183 |
| Male | Costa Rica | 5.827(5.314-6.389) | 6.125(4.609-8.141) | 6.439(3.982-10.414) | 6.77(3.438-13.328) | 7.117(2.969-17.062) | 127.4497 | 23.36714 |
| Male | El Salvador | 3.803(3.489-4.145) | 3.601(2.76-4.699) | 3.41(2.176-5.346) | 3.23(1.714-6.086) | 3.059(1.35-6.929) | 205.2523 | -21.664 |
| Male | Guatemala | 3.303(3.093-3.528) | 3.255(2.657-3.988) | 3.208(2.276-4.521) | 3.161(1.949-5.127) | 3.115(1.669-5.814) | 178.6706 | -6.29723 |
| Male | Honduras | 2.39(2.321-2.46) | 2.641(2.414-2.889) | 2.919(2.508-3.397) | 3.226(2.605-3.994) | 3.565(2.705-4.697) | 102.0913 | 52.86616 |
| Male | Mexico | 4.85(4.644-5.066) | 5.955(5.207-6.81) | 7.311(5.827-9.172) | 8.975(6.52-12.355) | 11.019(7.294-16.645) | 18.82234 | 137.3644 |
| Male | Nicaragua | 4.427(4.208-4.658) | 4.772(4.08-5.581) | 5.144(3.948-6.702) | 5.544(3.818-8.05) | 5.976(3.693-9.67) | 130.2405 | 37.03797 |
| Male | Panama | 3.728(3.459-4.019) | 3.914(3.104-4.934) | 4.108(2.777-6.078) | 4.312(2.483-7.49) | 4.527(2.22-9.23) | 94.83235 | 22.57251 |
| Male | Venezuela (Bolivarian Republic of) | 5.005(4.562-5.491) | 6.375(5.092-7.979) | 8.119(5.663-11.639) | 10.34(6.293-16.989) | 13.169(6.992-24.804) | 285.6642 | 178.008 |
| Female | Colombia | 3.701(3.607-3.798) | 3.905(3.606-4.23) | 4.121(3.6-4.716) | 4.348(3.595-5.259) | 4.588(3.589-5.865) | -10.0964 | 24.91539 |
| Female | Costa Rica | 5.082(4.726-5.465) | 5.171(4.132-6.471) | 5.261(3.601-7.686) | 5.352(3.137-9.132) | 5.446(2.733-10.852) | 135.2319 | 7.518207 |
| Female | El Salvador | 3.857(3.669-4.055) | 4.064(3.483-4.743) | 4.283(3.3-5.56) | 4.514(3.125-6.519) | 4.756(2.959-7.645) | 209.7464 | 24.35098 |
| Female | Guatemala | 3.383(3.179-3.6) | 3.591(2.963-4.352) | 3.812(2.755-5.274) | 4.047(2.561-6.395) | 4.296(2.38-7.754) | 233.4463 | 28.46906 |
| Female | Honduras | 3.884(3.689-4.088) | 4.271(3.645-5.005) | 4.697(3.593-6.14) | 5.165(3.541-7.535) | 5.68(3.489-9.247) | 122.8619 | 49.54433 |
| Female | Mexico | 4.405(4.263-4.553) | 5.013(4.528-5.549) | 5.704(4.804-6.773) | 6.491(5.095-8.268) | 7.386(5.404-10.094) | 4.164494 | 72.01094 |
| Female | Nicaragua | 3.904(3.615-4.216) | 4.223(3.33-5.355) | 4.567(3.058-6.822) | 4.94(2.806-8.696) | 5.343(2.575-11.086) | 148.4627 | 39.0286 |
| Female | Panama | 3.648(3.488-3.815) | 4.211(3.667-4.835) | 4.86(3.847-6.14) | 5.61(4.035-7.798) | 6.475(4.233-9.904) | 105.9458 | 83.56003 |
| Female | Venezuela (Bolivarian Republic of) | 3.954(3.671-4.259) | 3.777(3.003-4.751) | 3.608(2.448-5.317) | 3.447(1.996-5.953) | 3.293(1.627-6.665) | 290.939 | -18.7541 |


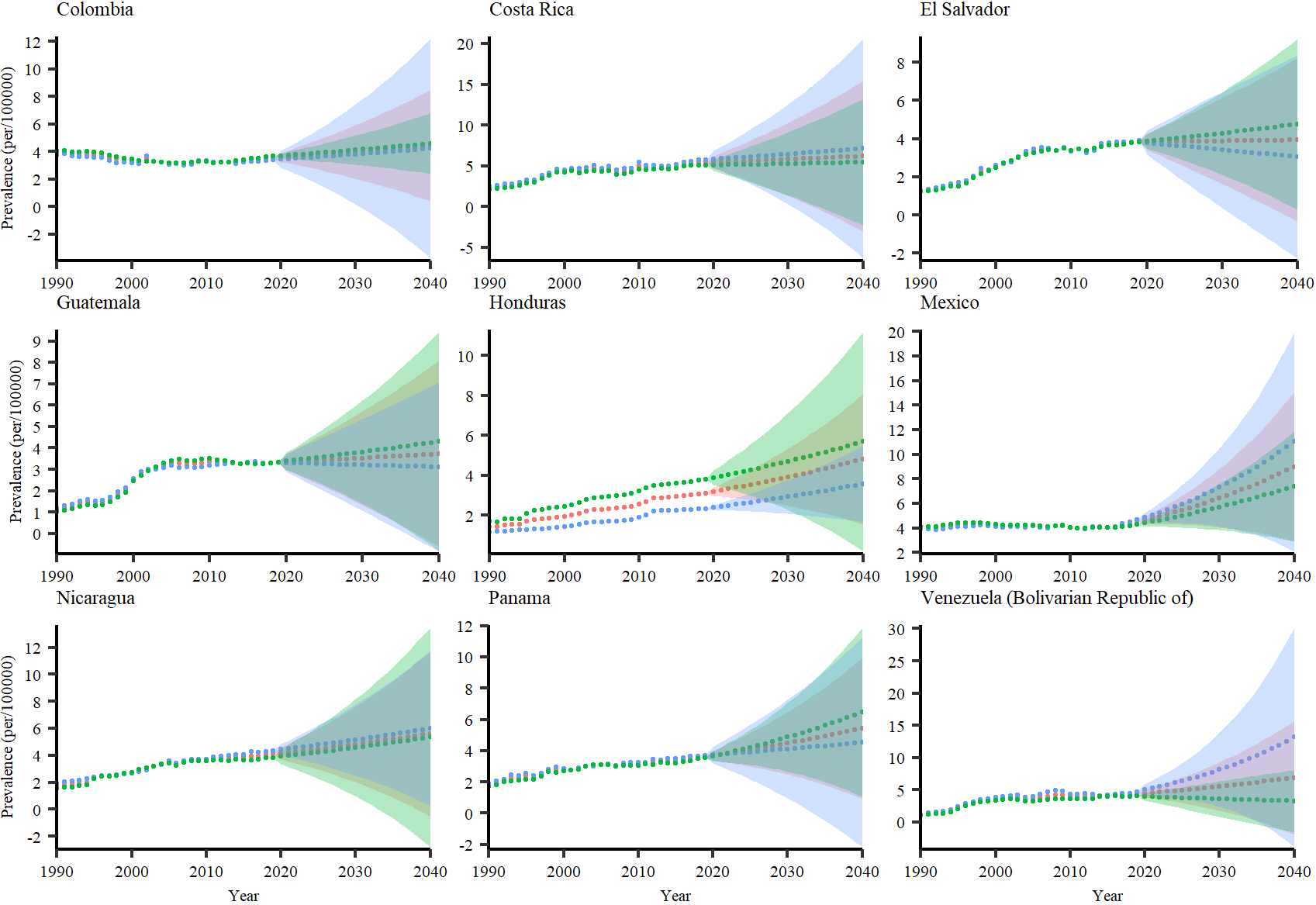


Supplemental Figure 12. Observed and projected age-standardized prevalence rate (ASPR) values from 1990 to 2040 for both sex (Red lines), females (Green lines), and men (Blue lines) in the Central Latin America. The halo effect observed in each scatter plot accurately represents projections that extend across the temporal span from 2019 to 2040 with 95% confidence intervals.


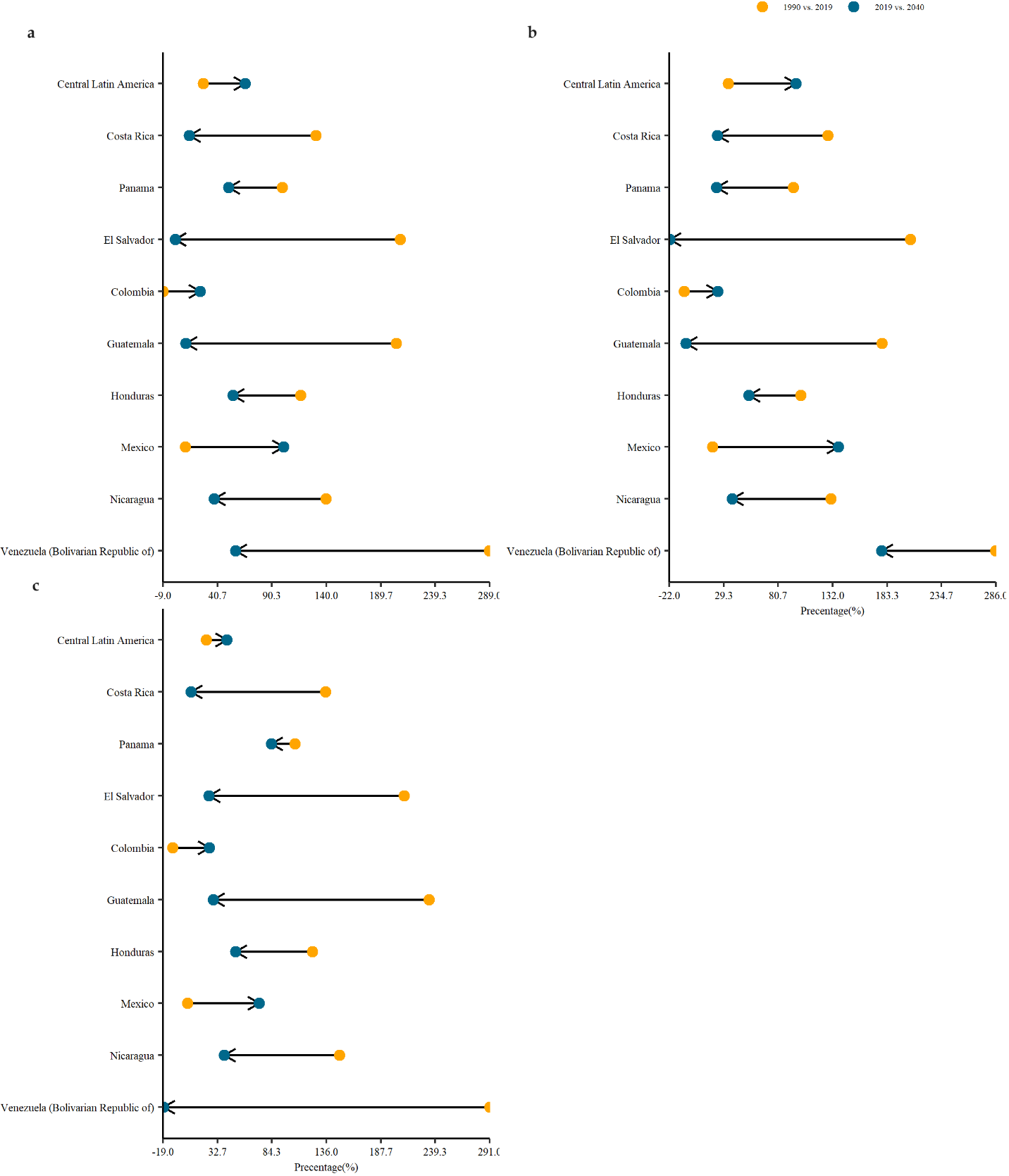


Supplemental Figure 13. The Lollipop plot between the two calculated percentage changes from 1990 to 2019 and 2019 to 2040 for both sexes (a), males (b), and females (c) in the Central Latin America. Each line represents two time periods and show the change of ASPR increase or decrease during time.

# Central Sub-Saharan Africa

| Supplemental Table 7: Age-standardized prevalence rates (ASPR) from 2020 to 2040, and percentage changes for the time periods 1990 to 2019 and 2019 to 2040, for Central Sub-Saharan Africa. | | | | | | | | |
| --- | --- | --- | --- | --- | --- | --- | --- | --- |
| Group | Country | 2020 | 2025 | 2030 | 2035 | 2040 | 1990 vs. 2019 | 2019 vs. 2040 |
| Both | Angola | 2.442(2.385-2.501) | 2.864(2.663-3.081) | 3.359(2.971-3.799) | 3.94(3.313-4.685) | 4.62(3.694-5.779) | 56.785 | 95.98529 |
| Both | Central African Republic | 1.537(1.517-1.558) | 1.562(1.499-1.629) | 1.588(1.48-1.704) | 1.614(1.461-1.782) | 1.64(1.442-1.864) | 2.185122 | 7.294729 |
| Both | Congo | 3.87(3.829-3.912) | 4.253(4.114-4.397) | 4.674(4.419-4.945) | 5.137(4.746-5.56) | 5.646(5.097-6.253) | 39.66606 | 48.85893 |
| Both | Democratic Republic of the Congo | 1.727(1.699-1.755) | 2.023(1.923-2.127) | 2.369(2.175-2.58) | 2.775(2.461-3.13) | 3.25(2.783-3.796) | 12.59126 | 94.76064 |
| Both | Equatorial Guinea | 4.459(4.37-4.55) | 4.994(4.694-5.315) | 5.594(5.036-6.213) | 6.265(5.403-7.265) | 7.017(5.797-8.494) | 225.7585 | 62.24267 |
| Both | Gabon | 5.864(5.778-5.951) | 6.592(6.299-6.899) | 7.411(6.863-8.003) | 8.332(7.477-9.285) | 9.368(8.146-10.773) | 100.3285 | 63.93199 |
| Male | Angola | 3.171(3.094-3.251) | 3.653(3.383-3.944) | 4.207(3.696-4.789) | 4.846(4.037-5.816) | 5.581(4.409-7.064) | 46.54103 | 81.62604 |
| Male | Central African Republic | 2.154(2.128-2.18) | 2.154(2.076-2.234) | 2.154(2.024-2.292) | 2.154(1.974-2.351) | 2.154(1.924-2.411) | -0.83016 | 0.242268 |
| Male | Congo | 4.367(4.32-4.415) | 4.834(4.675-4.999) | 5.351(5.057-5.662) | 5.924(5.47-6.414) | 6.557(5.917-7.266) | 17.35294 | 53.45339 |
| Male | Democratic Republic of the Congo | 2.18(2.145-2.214) | 2.498(2.378-2.623) | 2.862(2.635-3.109) | 3.28(2.919-3.685) | 3.759(3.234-4.369) | 6.845409 | 77.70416 |
| Male | Equatorial Guinea | 5.079(4.96-5.2) | 5.831(5.421-6.271) | 6.695(5.92-7.571) | 7.686(6.463-9.141) | 8.825(7.055-11.038) | 137.2717 | 80.43536 |
| Male | Gabon | 7.228(7.132-7.325) | 8.125(7.798-8.467) | 9.134(8.521-9.792) | 10.268(9.31-11.326) | 11.544(10.172-13.1) | 86.95727 | 63.87701 |
| Female | Angola | 1.841(1.799-1.884) | 2.227(2.074-2.391) | 2.694(2.389-3.038) | 3.259(2.752-3.86) | 3.943(3.17-4.905) | 92.90635 | 123.33 |
| Female | Central African Republic | 1.022(1.004-1.04) | 1.069(1.013-1.127) | 1.118(1.021-1.224) | 1.169(1.029-1.328) | 1.223(1.037-1.441) | 9.399207 | 21.14684 |
| Female | Congo | 3.383(3.332-3.435) | 3.665(3.498-3.841) | 3.971(3.669-4.297) | 4.302(3.848-4.809) | 4.66(4.036-5.381) | 68.66953 | 40.13582 |
| Female | Democratic Republic of the Congo | 1.345(1.32-1.369) | 1.601(1.514-1.694) | 1.907(1.734-2.098) | 2.272(1.987-2.598) | 2.706(2.276-3.217) | 23.59824 | 108.9102 |
| Female | Equatorial Guinea | 3.993(3.924-4.064) | 4.362(4.131-4.606) | 4.765(4.347-5.224) | 5.205(4.573-5.925) | 5.686(4.81-6.72) | 421.7112 | 45.79167 |
| Female | Gabon | 4.572(4.485-4.661) | 5.173(4.874-5.49) | 5.852(5.293-6.471) | 6.621(5.747-7.629) | 7.492(6.24-8.994) | 113.2085 | 68.30731 |


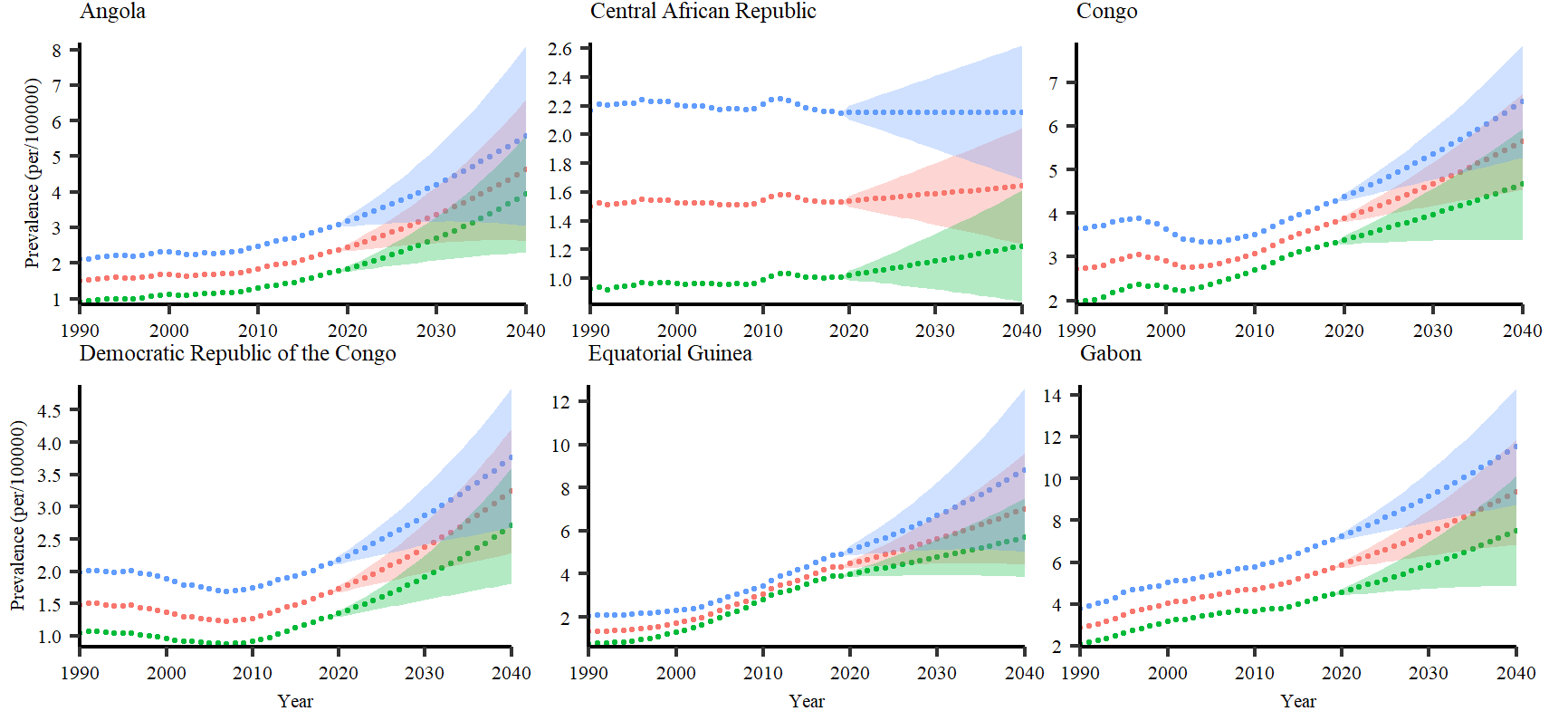


Supplemental Figure 14. Observed and projected age-standardized prevalence rate (ASPR) values from 1990 to 2040 for both sex (Red lines), females (Green lines), and men (Blue lines) in the Central Sub-Saharan Africa. The halo effect observed in each scatter plot accurately represents projections that extend across the temporal span from 2019 to 2040 with 95% confidence intervals.


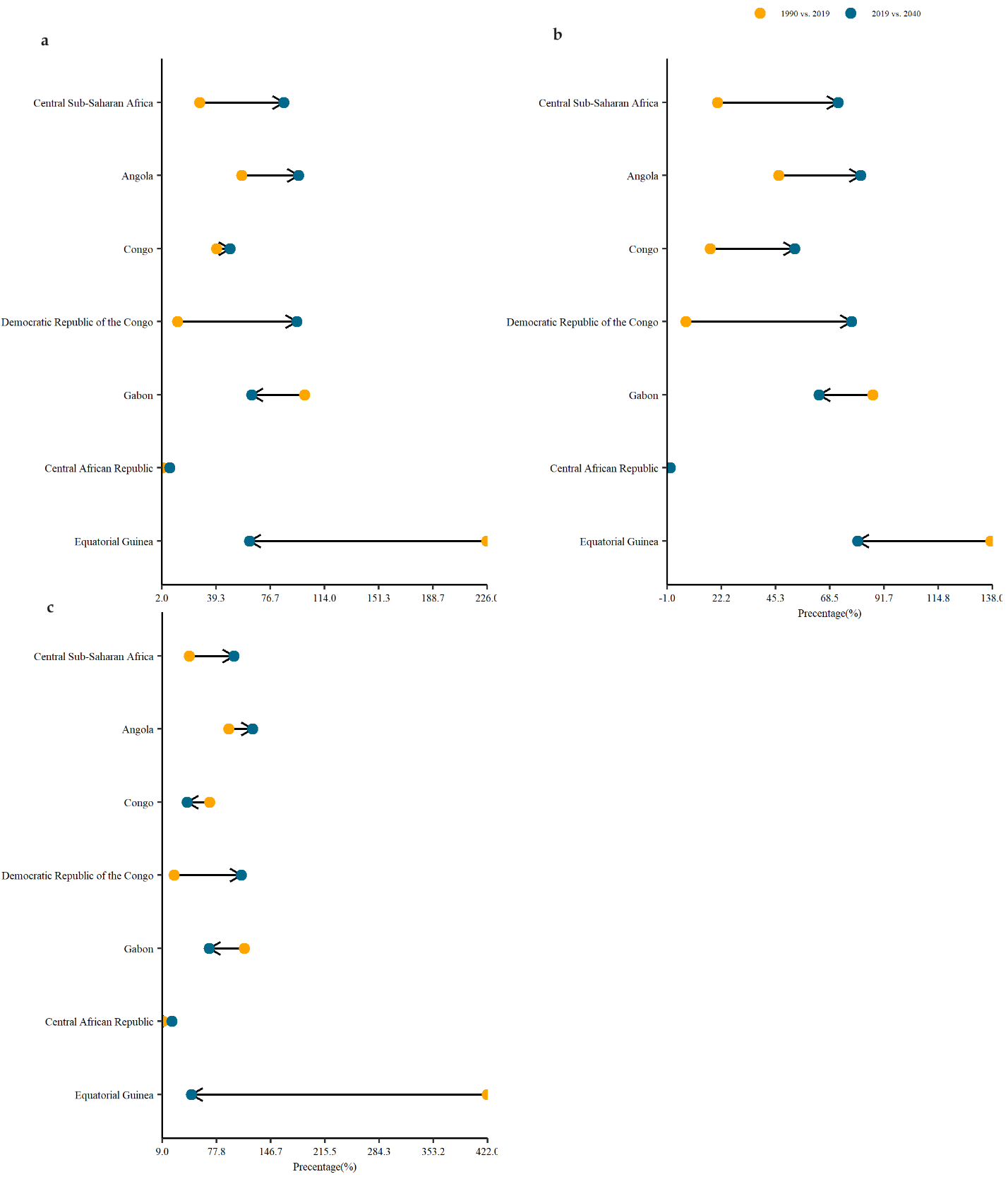


Supplemental Figure 15. The Lollipop plot between the two calculated percentage changes from 1990 to 2019 and 2019 to 2040 for both sexes (a), males (b), and females (c) in the Central Sub-Saharan Africa. Each line represents two time periods and show the change of ASPR increase or decrease during time.

# East Asia

| Supplemental Table 8: Age-standardized prevalence rates (ASPR) from 2020 to 2040, and percentage changes for the time periods 1990 to 2019 and 2019 to 2040, for East Asia. | | | | | | | | |
| --- | --- | --- | --- | --- | --- | --- | --- | --- |
| Group | Country | 2020 | 2025 | 2030 | 2035 | 2040 | 1990 vs. 2019 | 2019 vs. 2040 |
| Both | China | 4.664(4.599-4.73) | 5.721(5.479-5.974) | 7.018(6.523-7.55) | 8.608(7.765-9.542) | 10.559(9.244-12.061) | 85.41198 | 136.8183 |
| Both | Democratic People's Republic of Korea | 3.058(3.048-3.069) | 3.185(3.152-3.219) | 3.317(3.259-3.377) | 3.455(3.369-3.542) | 3.598(3.483-3.716) | 32.63812 | 18.71978 |
| Both | Taiwan (Province of China) | 7.219(6.992-7.453) | 7.466(6.766-8.239) | 7.722(6.539-9.12) | 7.987(6.318-10.098) | 8.261(6.104-11.181) | 113.1169 | 15.32344 |
| Male | China | 5.977(5.891-6.064) | 7.164(6.851-7.492) | 8.587(7.962-9.261) | 10.292(9.252-11.449) | 12.336(10.751-14.155) | 95.57307 | 114.6671 |
| Male | Democratic People's Republic of Korea | 3.743(3.73-3.756) | 3.827(3.785-3.869) | 3.912(3.84-3.986) | 4(3.897-4.106) | 4.09(3.954-4.231) | 23.01644 | 9.853708 |
| Male | Taiwan (Province of China) | 9.079(8.735-9.436) | 9.369(8.318-10.554) | 9.669(7.907-11.825) | 9.979(7.515-13.251) | 10.299(7.142-14.851) | 114.3114 | 14.30665 |
| Female | China | 3.385(3.299-3.473) | 3.976(3.736-4.231) | 4.67(4.227-5.16) | 5.486(4.781-6.295) | 6.445(5.408-7.68) | 72.45524 | 94.92543 |
| Female | Democratic People's Republic of Korea | 2.496(2.487-2.504) | 2.629(2.601-2.658) | 2.77(2.721-2.821) | 2.919(2.845-2.995) | 3.076(2.976-3.179) | 38.37173 | 24.69647 |
| Female | Taiwan (Province of China) | 5.478(5.309-5.654) | 5.709(5.181-6.291) | 5.949(5.049-7.01) | 6.2(4.92-7.812) | 6.46(4.793-8.707) | 123.7687 | 18.95264 |


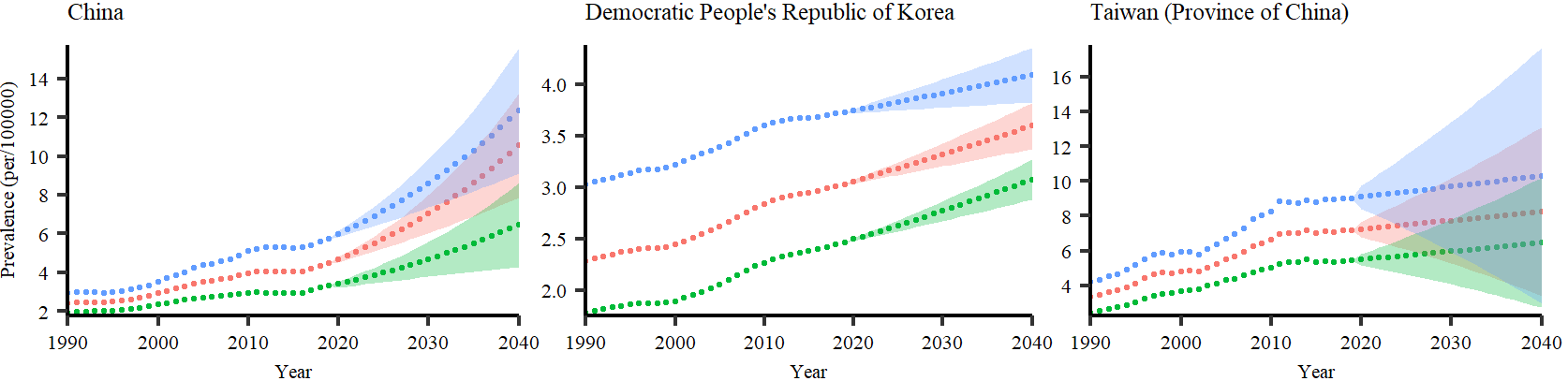


Supplemental Figure 16. Observed and projected age-standardized prevalence rate (ASPR) values from 1990 to 2040 for both sex (Red lines), females (Green lines), and men (Blue lines) in the East Asia. The halo effect observed in each scatter plot accurately represents projections that extend across the temporal span from 2019 to 2040 with 95% confidence intervals.


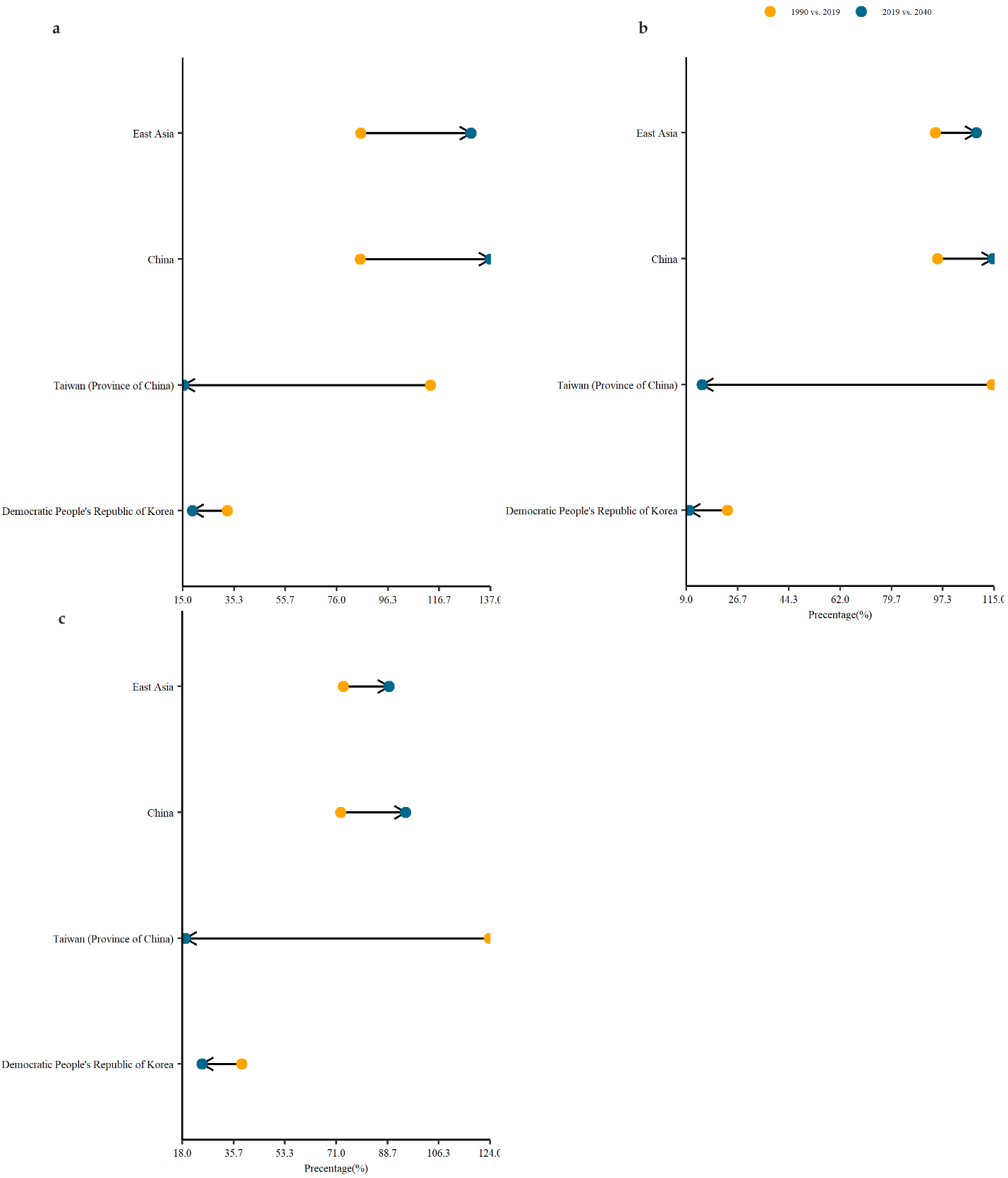


Supplemental Figure 17. The Lollipop plot between the two calculated percentage changes from 1990 to 2019 and 2019 to 2040 for both sexes (a), males (b), and females (c) in the East Asia. Each line represents two time periods and show the change of ASPR increase or decrease during time.

# Eastern Europe

| Supplemental Table 9: Age-standardized prevalence rates (ASPR) from 2020 to 2040, and percentage changes for the time periods 1990 to 2019 and 2019 to 2040, for Eastern Europe. | | | | | | | | |
| --- | --- | --- | --- | --- | --- | --- | --- | --- |
| Group | Country | 2020 | 2025 | 2030 | 2035 | 2040 | 1990 vs. 2019 | 2019 vs. 2040 |
| Both | Belarus | 5.777(5.413-6.166) | 6.088(4.979-7.444) | 6.415(4.566-9.012) | 6.759(4.187-10.913) | 7.123(3.838-13.217) | 50.31404 | 24.17176 |
| Both | Estonia | 9.224(8.308-10.241) | 9.491(6.871-13.109) | 9.765(5.658-16.854) | 10.048(4.656-21.683) | 10.338(3.831-27.9) | 32.77361 | 13.13948 |
| Both | Latvia | 7.785(6.944-8.728) | 8.613(6.051-12.259) | 9.529(5.248-17.301) | 10.542(4.548-24.433) | 11.663(3.941-34.513) | 28.23811 | 53.46019 |
| Both | Lithuania | 6.963(6.432-7.537) | 6.643(5.201-8.484) | 6.337(4.191-9.582) | 6.046(3.376-10.827) | 5.768(2.719-12.236) | 23.40345 | -18.4678 |
| Both | Republic of Moldova | 5.991(5.39-6.659) | 4.721(3.406-6.543) | 3.72(2.143-6.458) | 2.932(1.348-6.377) | 2.31(0.847-6.299) | 25.85286 | -64.0743 |
| Both | Russian Federation | 5.81(5.226-6.461) | 5.29(3.813-7.34) | 4.817(2.77-8.378) | 4.386(2.011-9.567) | 3.993(1.459-10.927) | 5.338062 | -33.7304 |
| Both | Ukraine | 6.85(6.16-7.616) | 6.243(4.5-8.663) | 5.691(3.272-9.898) | 5.187(2.378-11.316) | 4.728(1.728-12.939) | 75.32252 | -32.9451 |
| Male | Belarus | 7.804(7.254-8.396) | 7.895(6.301-9.893) | 7.987(5.456-11.693) | 8.08(4.722-13.826) | 8.174(4.087-16.351) | 38.56219 | 4.41758 |
| Male | Estonia | 11.166(9.89-12.607) | 11.14(7.659-16.201) | 11.113(5.901-20.929) | 11.087(4.543-27.053) | 11.06(3.497-34.979) | 16.40387 | -0.79316 |
| Male | Latvia | 9.664(8.485-11.007) | 10.381(6.947-15.512) | 11.151(5.656-21.983) | 11.978(4.602-31.177) | 12.867(3.744-44.225) | 15.18454 | 35.39759 |
| Male | Lithuania | 8.628(7.847-9.486) | 7.767(5.795-10.408) | 6.991(4.263-11.465) | 6.293(3.134-12.637) | 5.665(2.304-13.93) | 7.721024 | -36.251 |
| Male | Republic of Moldova | 8.555(7.587-9.647) | 6.612(4.563-9.58) | 5.11(2.73-9.562) | 3.949(1.633-9.551) | 3.052(0.976-9.542) | 32.71794 | -66.8506 |
| Male | Russian Federation | 7.545(6.667-8.538) | 6.497(4.435-9.517) | 5.594(2.935-10.664) | 4.817(1.94-11.958) | 4.148(1.283-13.412) | -2.56618 | -47.7659 |
| Male | Ukraine | 10.123(8.879-11.54) | 8.955(5.975-13.422) | 7.923(3.999-15.697) | 7.009(2.674-18.372) | 6.201(1.788-21.508) | 92.53865 | -40.9939 |
| Female | Belarus | 4.26(4.029-4.505) | 4.667(3.927-5.547) | 5.113(3.819-6.845) | 5.601(3.713-8.45) | 6.136(3.609-10.433) | 60.95072 | 46.5837 |
| Female | Estonia | 7.479(6.846-8.171) | 7.781(5.921-10.224) | 8.094(5.102-12.841) | 8.421(4.394-16.136) | 8.76(3.784-20.28) | 47.43186 | 18.63706 |
| Female | Latvia | 6.259(5.673-6.906) | 7.06(5.212-9.562) | 7.963(4.769-13.297) | 8.982(4.361-18.5) | 10.131(3.987-25.744) | 39.21263 | 66.68613 |
| Female | Lithuania | 5.706(5.307-6.136) | 5.753(4.598-7.198) | 5.8(3.971-8.471) | 5.848(3.429-9.972) | 5.895(2.96-11.741) | 43.18466 | 3.178161 |
| Female | Republic of Moldova | 3.944(3.609-4.31) | 3.19(2.425-4.196) | 2.58(1.623-4.101) | 2.086(1.086-4.009) | 1.687(0.726-3.92) | 12.63144 | -59.9849 |
| Female | Russian Federation | 4.517(4.17-4.893) | 4.318(3.374-5.526) | 4.127(2.72-6.263) | 3.945(2.192-7.101) | 3.772(1.767-8.052) | 11.44902 | -18.3537 |
| Female | Ukraine | 4.378(4.097-4.677) | 4.273(3.483-5.242) | 4.171(2.952-5.892) | 4.071(2.502-6.624) | 3.973(2.12-7.449) | 47.45263 | -10.0893 |


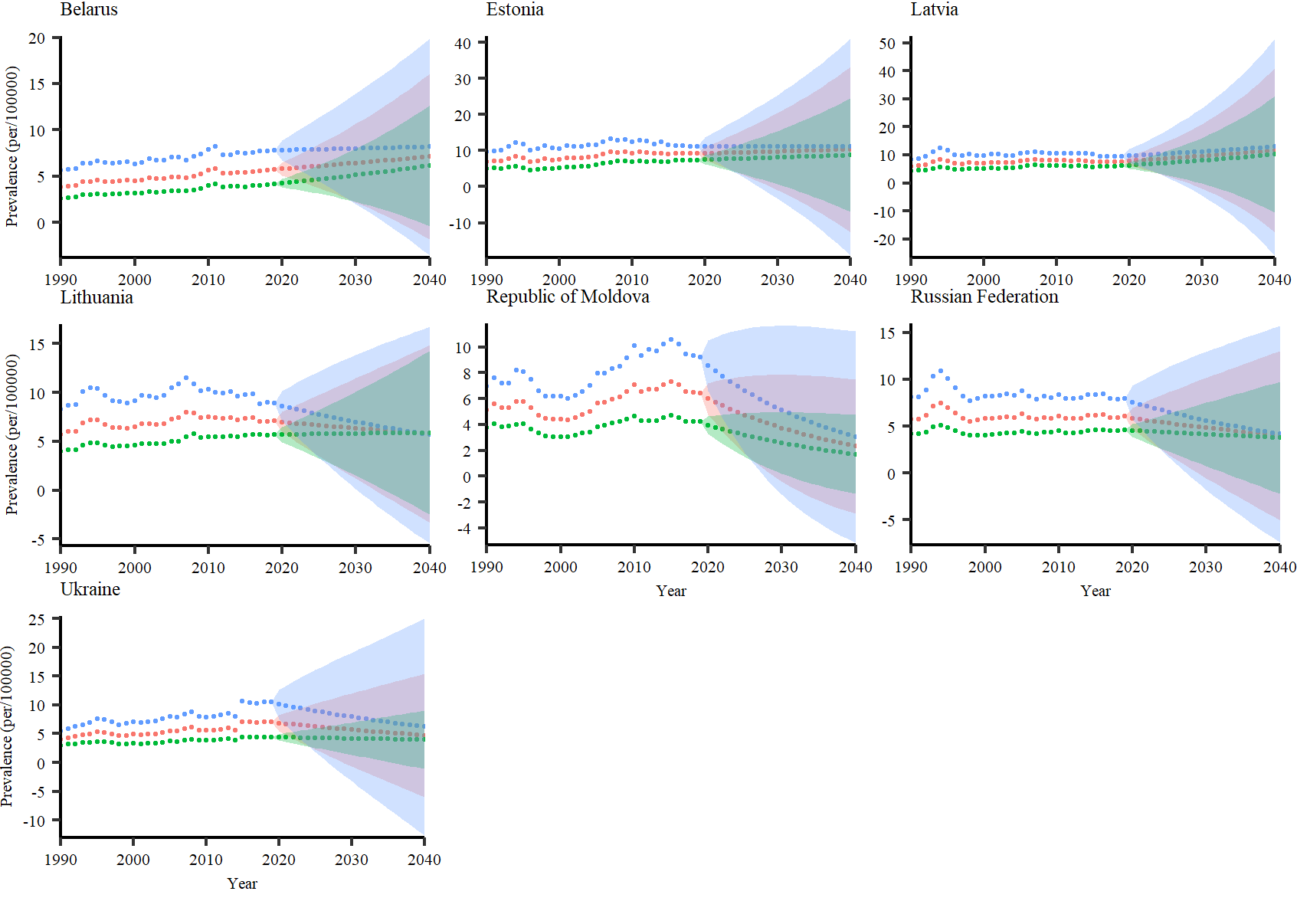


Supplemental Figure 18. Observed and projected age-standardized prevalence rate (ASPR) values from 1990 to 2040 for both sex (Red lines), females (Green lines), and men (Blue lines) in the Eastern Europe. The halo effect observed in each scatter plot accurately represents projections that extend across the temporal span from 2019 to 2040 with 95% confidence intervals.


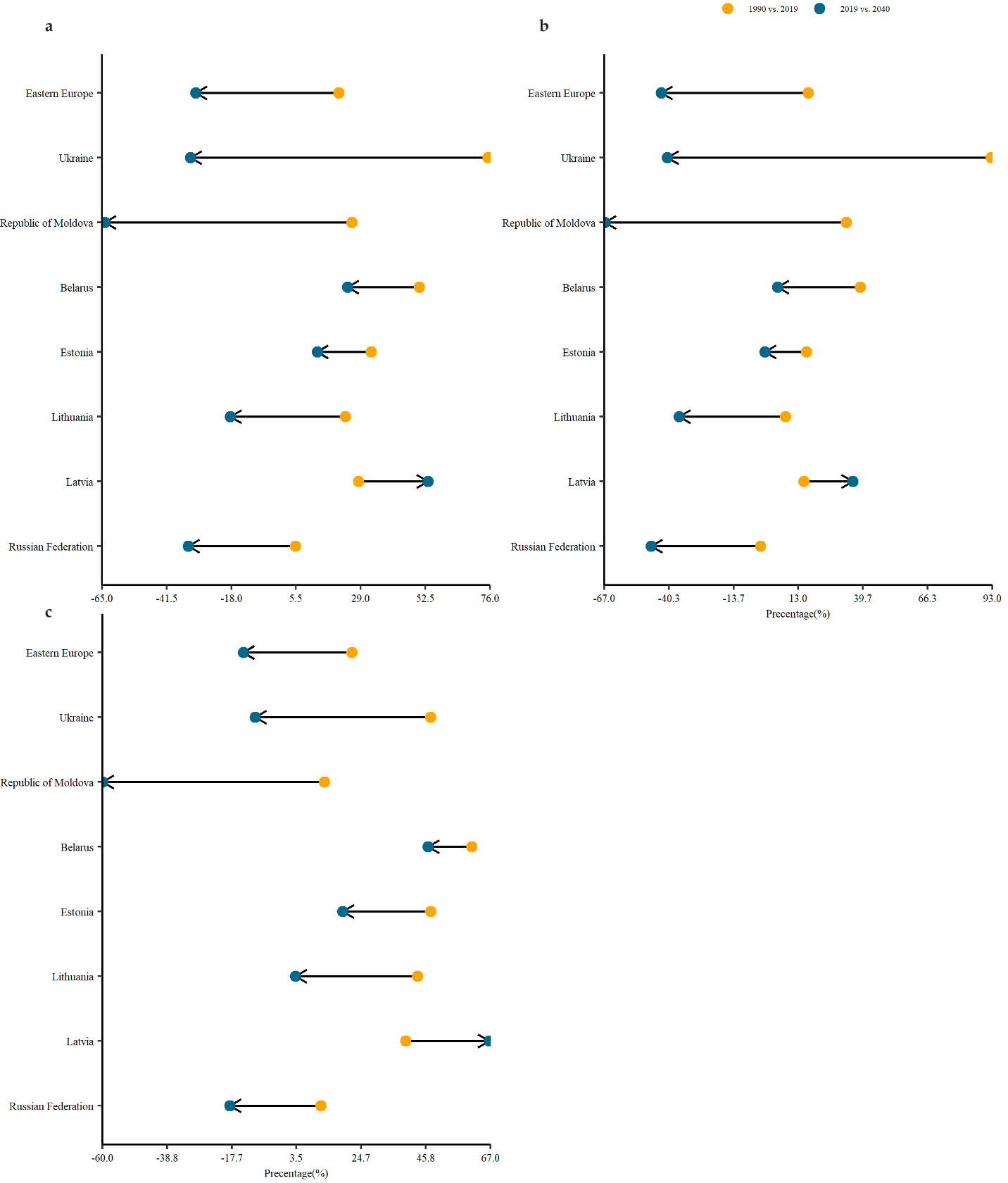


Supplemental Figure 19. The Lollipop plot between the two calculated percentage changes from 1990 to 2019 and 2019 to 2040 for both sexes (a), males (b), and females (c) in the Eastern Europe. Each line represents two time periods and show the change of ASPR increase or decrease during time.
