# Supplementary material for: Global Trend in Pancreatic Cancer Prevalence Rates Through 2040: An Illness‐Death Modeling Study
Source: Cancer Med. 2024 Oct 23;13(20):e70318. doi: 10.1002/cam4.70318 (PMC11497012; doi:10.1002/cam4.70318)
Supplement: Supplementary file 3 — Data S3. [file CAM4-13-e70318-s001.docx]

# Eastern Sub-Saharan Africa

| Supplemental Table 10: Age-standardized prevalence rates (ASPR) from 2020 to 2040, and percentage changes for the time periods 1990 to 2019 and 2019 to 2040, for Eastern Sub-Saharan Africa. | | | | | | | | |
| --- | --- | --- | --- | --- | --- | --- | --- | --- |
| Group | Country | 2020 | 2025 | 2030 | 2035 | 2040 | 1990 vs. 2019 | 2019 vs. 2040 |
| Both | Burundi | 1.619(1.606-1.632) | 1.691(1.649-1.734) | 1.766(1.693-1.842) | 1.844(1.737-1.958) | 1.927(1.783-2.081) | -2.57369 | 20.3121 |
| Both | Comoros | 2.681(2.591-2.773) | 2.677(2.411-2.972) | 2.673(2.24-3.189) | 2.669(2.08-3.423) | 2.664(1.932-3.675) | 36.12013 | -0.84679 |
| Both | Djibouti | 3.091(3.033-3.15) | 3.622(3.417-3.84) | 4.246(3.847-4.686) | 4.976(4.331-5.719) | 5.833(4.875-6.979) | 67.14444 | 95.65986 |
| Both | Eritrea | 2.226(2.195-2.257) | 2.467(2.364-2.575) | 2.735(2.544-2.939) | 3.031(2.738-3.355) | 3.36(2.947-3.83) | 74.3904 | 54.11657 |
| Both | Ethiopia | 1.105(1.089-1.122) | 1.303(1.244-1.365) | 1.537(1.421-1.663) | 1.813(1.623-2.025) | 2.138(1.854-2.467) | 26.1724 | 100.4348 |
| Both | Kenya | 2.27(2.235-2.306) | 2.612(2.489-2.74) | 3.004(2.77-3.259) | 3.456(3.082-3.876) | 3.976(3.429-4.61) | 115.5768 | 80.09072 |
| Both | Madagascar | 1.721(1.683-1.76) | 1.892(1.765-2.027) | 2.079(1.85-2.337) | 2.286(1.939-2.695) | 2.513(2.032-3.107) | 21.17758 | 49.00437 |
| Both | Malawi | 2.147(2.124-2.171) | 2.34(2.263-2.42) | 2.55(2.409-2.699) | 2.778(2.564-3.01) | 3.027(2.73-3.357) | 52.46602 | 43.563 |
| Both | Mozambique | 2.648(2.617-2.68) | 2.91(2.805-3.019) | 3.198(3.005-3.402) | 3.514(3.219-3.835) | 3.861(3.449-4.323) | 107.1938 | 48.95767 |
| Both | Rwanda | 2.561(2.433-2.695) | 2.98(2.545-3.489) | 3.468(2.657-4.526) | 4.036(2.772-5.874) | 4.696(2.893-7.624) | 16.93399 | 89.02273 |
| Both | Somalia | 1.169(1.159-1.178) | 1.133(1.106-1.162) | 1.099(1.054-1.146) | 1.066(1.005-1.13) | 1.033(0.958-1.115) | -8.71731 | -12.147 |
| Both | South Sudan | 2.055(2.025-2.085) | 2.211(2.113-2.314) | 2.379(2.204-2.569) | 2.561(2.299-2.852) | 2.756(2.398-3.167) | 12.79255 | 36.44398 |
| Both | United Republic of Tanzania | 2.812(2.762-2.862) | 3.402(3.22-3.594) | 4.116(3.752-4.515) | 4.98(4.37-5.674) | 6.025(5.091-7.13) | 42.22623 | 124.0981 |
| Both | Uganda | 3.594(3.546-3.643) | 4.064(3.899-4.236) | 4.595(4.284-4.929) | 5.196(4.708-5.735) | 5.875(5.172-6.674) | 108.6114 | 67.81054 |
| Both | Zambia | 3.691(3.638-3.745) | 4.258(4.071-4.453) | 4.912(4.553-5.299) | 5.666(5.092-6.305) | 6.536(5.694-7.503) | 47.18021 | 83.06411 |
| Male | Burundi | 1.93(1.913-1.948) | 1.995(1.941-2.051) | 2.062(1.968-2.16) | 2.131(1.995-2.276) | 2.202(2.023-2.397) | -12.4907 | 15.06304 |
| Male | Comoros | 2.884(2.781-2.992) | 2.831(2.528-3.17) | 2.778(2.294-3.363) | 2.726(2.082-3.569) | 2.675(1.889-3.788) | 22.26032 | -7.82439 |
| Male | Djibouti | 3.406(3.343-3.471) | 3.929(3.707-4.164) | 4.532(4.108-4.999) | 5.227(4.552-6.002) | 6.029(5.043-7.206) | 56.43449 | 82.87636 |
| Male | Eritrea | 2.394(2.359-2.43) | 2.586(2.469-2.707) | 2.792(2.583-3.018) | 3.015(2.702-3.364) | 3.255(2.826-3.75) | 40.42873 | 38.07715 |
| Male | Ethiopia | 1.292(1.276-1.308) | 1.491(1.435-1.549) | 1.721(1.613-1.836) | 1.987(1.814-2.176) | 2.293(2.039-2.579) | 9.302549 | 83.18342 |
| Male | Kenya | 2.106(2.073-2.14) | 2.327(2.216-2.443) | 2.571(2.367-2.792) | 2.84(2.529-3.19) | 3.138(2.701-3.646) | 102.8364 | 52.00188 |
| Male | Madagascar | 1.838(1.796-1.88) | 1.971(1.837-2.114) | 2.113(1.877-2.379) | 2.266(1.918-2.677) | 2.43(1.959-3.014) | 13.26238 | 34.23144 |
| Male | Malawi | 2.357(2.324-2.39) | 2.481(2.376-2.591) | 2.612(2.428-2.811) | 2.75(2.48-3.049) | 2.895(2.534-3.308) | 48.29074 | 24.28642 |
| Male | Mozambique | 2.982(2.946-3.018) | 3.198(3.082-3.318) | 3.429(3.222-3.65) | 3.678(3.368-4.016) | 3.944(3.52-4.418) | 101.5908 | 34.47764 |
| Male | Rwanda | 2.828(2.661-3.005) | 3.208(2.66-3.869) | 3.64(2.652-4.996) | 4.13(2.643-6.453) | 4.685(2.634-8.335) | 1.405923 | 69.85818 |
| Male | Somalia | 1.728(1.718-1.738) | 1.666(1.635-1.697) | 1.605(1.556-1.657) | 1.547(1.48-1.618) | 1.492(1.409-1.579) | -8.7101 | -14.2469 |
| Male | South Sudan | 2.337(2.303-2.372) | 2.463(2.353-2.579) | 2.596(2.403-2.805) | 2.736(2.453-3.052) | 2.884(2.505-3.32) | 7.787855 | 24.95626 |
| Male | United Republic of Tanzania | 3.162(3.112-3.212) | 3.776(3.598-3.963) | 4.51(4.156-4.894) | 5.387(4.8-6.044) | 6.433(5.544-7.465) | 29.96683 | 112.1192 |
| Male | Uganda | 4.601(4.549-4.653) | 4.994(4.823-5.171) | 5.421(5.111-5.749) | 5.884(5.416-6.393) | 6.388(5.739-7.109) | 90.70595 | 41.34115 |
| Male | Zambia | 4.246(4.185-4.308) | 4.824(4.613-5.044) | 5.48(5.081-5.91) | 6.225(5.597-6.924) | 7.072(6.164-8.113) | 45.28431 | 71.65736 |
| Female | Eastern Sub-Saharan Africa | 1.932(1.918-1.947) | 2.275(2.223-2.33) | 2.68(2.575-2.788) | 3.155(2.983-3.337) | 3.716(3.456-3.995) | 75.13864 | 99.21063 |
| Female | Burundi | 1.273(1.264-1.283) | 1.344(1.313-1.375) | 1.418(1.363-1.475) | 1.496(1.416-1.581) | 1.579(1.47-1.696) | 5.311673 | 25.5309 |
| Female | Comoros | 2.493(2.414-2.574) | 2.524(2.285-2.788) | 2.555(2.16-3.023) | 2.587(2.041-3.278) | 2.619(1.929-3.555) | 56.19303 | 5.165742 |
| Female | Djibouti | 2.702(2.649-2.755) | 3.252(3.06-3.456) | 3.914(3.532-4.338) | 4.711(4.076-5.445) | 5.671(4.704-6.836) | 79.67345 | 118.8338 |
| Female | Eritrea | 2.061(2.034-2.088) | 2.326(2.235-2.421) | 2.625(2.454-2.809) | 2.963(2.694-3.259) | 3.344(2.958-3.781) | 114.4165 | 66.25241 |
| Female | Ethiopia | 0.911(0.891-0.931) | 1.118(1.046-1.196) | 1.374(1.226-1.539) | 1.687(1.437-1.98) | 2.072(1.685-2.548) | 66.61868 | 137.5867 |
| Female | Kenya | 2.398(2.361-2.436) | 2.851(2.716-2.993) | 3.39(3.123-3.68) | 4.03(3.59-4.524) | 4.791(4.126-5.562) | 125.1182 | 106.7509 |
| Female | Madagascar | 1.608(1.568-1.649) | 1.815(1.679-1.962) | 2.05(1.797-2.339) | 2.315(1.923-2.787) | 2.614(2.058-3.322) | 32.57859 | 66.8216 |
| Female | Malawi | 1.966(1.948-1.984) | 2.208(2.145-2.273) | 2.48(2.361-2.604) | 2.785(2.599-2.984) | 3.128(2.862-3.419) | 57.49468 | 62.9928 |
| Female | Mozambique | 2.385(2.355-2.415) | 2.684(2.582-2.79) | 3.021(2.83-3.225) | 3.4(3.101-3.728) | 3.827(3.398-4.31) | 116.7045 | 64.70211 |
| Female | Rwanda | 2.343(2.256-2.433) | 2.778(2.472-3.123) | 3.294(2.704-4.014) | 3.907(2.957-5.161) | 4.633(3.234-6.636) | 37.35567 | 104.6244 |
| Female | Somalia | 0.753(0.744-0.763) | 0.733(0.706-0.762) | 0.714(0.669-0.762) | 0.695(0.635-0.762) | 0.677(0.602-0.761) | 2.2452 | -10.6344 |
| Female | South Sudan | 1.746(1.719-1.772) | 1.961(1.871-2.056) | 2.203(2.035-2.386) | 2.475(2.213-2.769) | 2.781(2.406-3.213) | 27.68592 | 63.50234 |
| Female | United Republic of Tanzania | 2.482(2.43-2.535) | 3.051(2.858-3.256) | 3.75(3.359-4.186) | 4.609(3.947-5.382) | 5.665(4.637-6.921) | 60.46771 | 139.6211 |
| Female | Uganda | 2.77(2.719-2.823) | 3.305(3.12-3.5) | 3.942(3.577-4.345) | 4.703(4.101-5.393) | 5.61(4.701-6.694) | 161.6949 | 110.2163 |
| Female | Zambia | 3.153(3.105-3.201) | 3.696(3.526-3.874) | 4.333(4.002-4.692) | 5.08(4.542-5.682) | 5.956(5.154-6.882) | 53.4079 | 95.93643 |


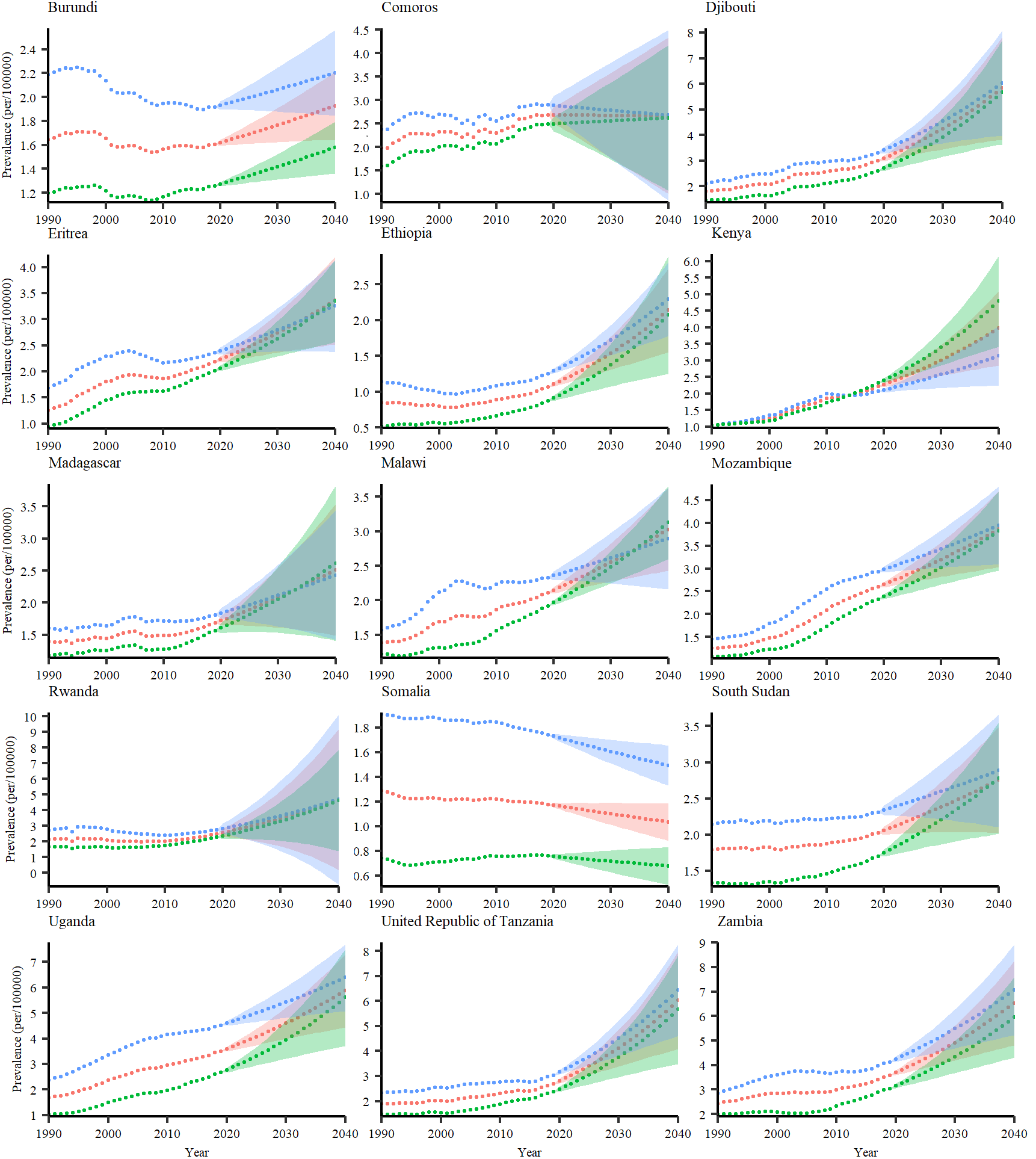


Supplemental Figure 20. Observed and projected age-standardized prevalence rate (ASPR) values from 1990 to 2040 for both sex (Red lines), females (Green lines), and men (Blue lines) in the Eastern Sub-Saharan Africa. The halo effect observed in each scatter plot accurately represents projections that extend across the temporal span from 2019 to 2040 with 95% confidence intervals.


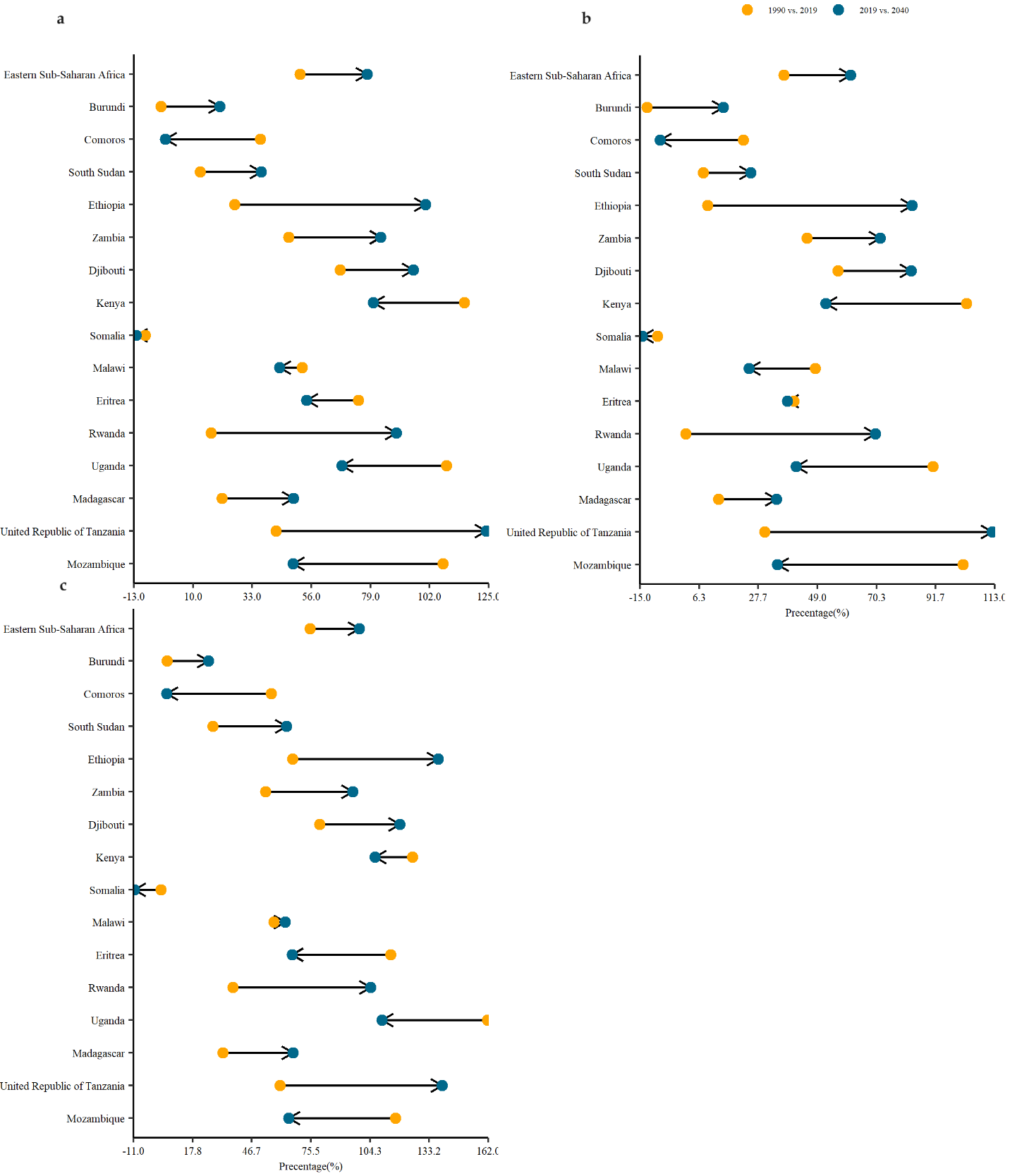


Supplemental Figure 21. The Lollipop plot between the two calculated percentage changes from 1990 to 2019 and 2019 to 2040 for both sexes (a), males (b), and females (c) in the Eastern Sub-Saharan Africa. Each line represents two time periods and show the change of ASPR increase or decrease during time.

# High-income Asia Pacific

| Supplemental Table 11: Age-standardized prevalence rates (ASPR) from 2020 to 2040, and percentage changes for the time periods 1990 to 2019 and 2019 to 2040, for High-income Asia Pacific. | | | | | | | | |
| --- | --- | --- | --- | --- | --- | --- | --- | --- |
| Group | Country | 2020 | 2025 | 2030 | 2035 | 2040 | 1990 vs. 2019 | 2019 vs. 2040 |
| Both | Brunei Darussalam | 7.344(7.225-7.465) | 7.431(7.066-7.814) | 7.519(6.906-8.186) | 7.607(6.749-8.575) | 7.697(6.595-8.984) | 51.39193 | 4.92703 |
| Both | Japan | 11.085(10.846-11.329) | 10.395(9.719-11.118) | 9.748(8.701-10.921) | 9.142(7.789-10.729) | 8.573(6.972-10.541) | 25.98555 | -23.9007 |
| Both | Republic of Korea | 8.922(8.782-9.065) | 9.594(9.136-10.075) | 10.317(9.499-11.205) | 11.094(9.874-12.464) | 11.929(10.265-13.864) | 46.3971 | 35.38144 |
| Both | Singapore | 5.933(5.739-6.134) | 5.749(5.188-6.372) | 5.572(4.683-6.628) | 5.399(4.227-6.896) | 5.232(3.815-7.175) | 34.44925 | -12.4571 |
| Male | Brunei Darussalam | 7.968(7.735-8.209) | 8.097(7.386-8.875) | 8.227(7.045-9.608) | 8.36(6.718-10.403) | 8.495(6.406-11.265) | 36.98184 | 6.814028 |
| Male | Japan | 12.959(12.634-13.292) | 11.985(11.081-12.962) | 11.084(9.709-12.654) | 10.251(8.505-12.355) | 9.48(7.45-12.064) | 16.56684 | -28.2551 |
| Male | Republic of Korea | 10.277(10.082-10.476) | 10.576(9.968-11.22) | 10.883(9.848-12.026) | 11.198(9.727-12.892) | 11.523(9.608-13.821) | 24.25812 | 12.35607 |
| Male | Singapore | 6.173(5.836-6.529) | 5.757(4.842-6.845) | 5.369(4.008-7.193) | 5.007(3.316-7.562) | 4.67(2.743-7.95) | 16.48451 | -25.3908 |
| Female | Brunei Darussalam | 7.061(6.91-7.216) | 7.031(6.576-7.517) | 7.001(6.253-7.839) | 6.971(5.945-8.174) | 6.941(5.652-8.525) | 71.97609 | -1.90285 |
| Female | Japan | 9.348(9.139-9.562) | 8.893(8.293-9.536) | 8.459(7.518-9.519) | 8.047(6.815-9.503) | 7.655(6.177-9.488) | 34.58603 | -19.1432 |
| Female | Republic of Korea | 7.668(7.53-7.808) | 8.55(8.086-9.041) | 9.534(8.676-10.478) | 10.632(9.308-12.144) | 11.855(9.985-14.075) | 71.33781 | 57.92008 |
| Female | Singapore | 5.686(5.413-5.972) | 5.728(4.922-6.666) | 5.771(4.466-7.457) | 5.813(4.05-8.343) | 5.857(3.674-9.336) | 54.74736 | 3.052963 |


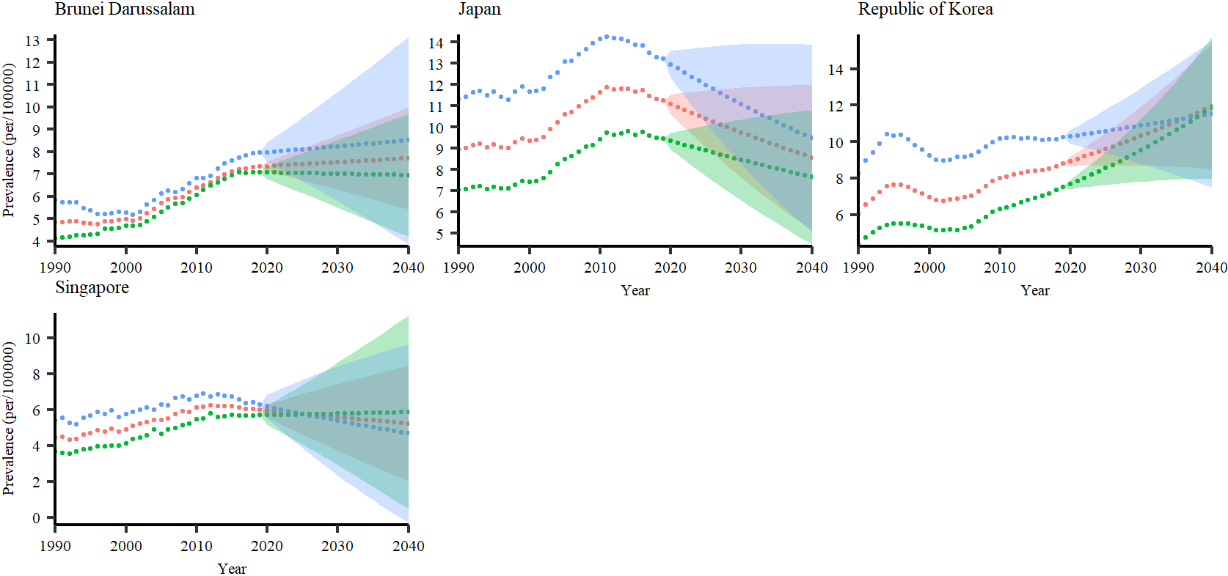


Supplemental Figure 22. Observed and projected age-standardized prevalence rate (ASPR) values from 1990 to 2040 for both sex (Red lines), females (Green lines), and men (Blue lines) in the High-income Asia Pacific. The halo effect observed in each scatter plot accurately represents projections that extend across the temporal span from 2019 to 2040 with 95% confidence intervals.


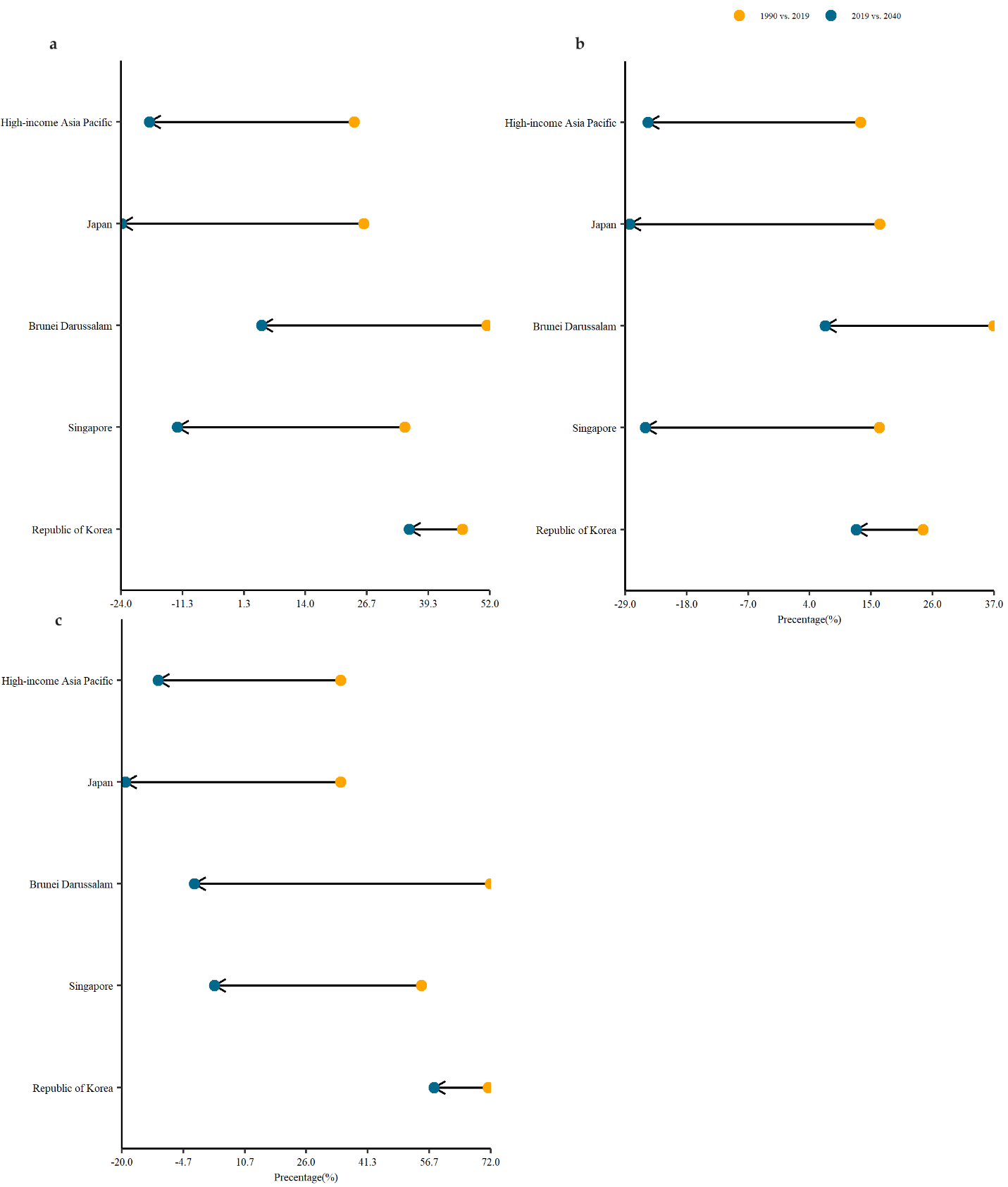


Supplemental Figure 23. The Lollipop plot between the two calculated percentage changes from 1990 to 2019 and 2019 to 2040 for both sexes (a), males (b), and females (c) in the High-income Asia Pacific. Each line represents two time periods and show the change of ASPR increase or decrease during time.

# High-income North America

| Supplemental Table 12: Age-standardized prevalence rates (ASPR) from 2020 to 2040, and percentage changes for the time periods 1990 to 2019 and 2019 to 2040, for High-income North America. | | | | | | | | |
| --- | --- | --- | --- | --- | --- | --- | --- | --- |
| Group | Country | 2020 | 2025 | 2030 | 2035 | 2040 | 1990 vs. 2019 | 2019 vs. 2040 |
| Both | Canada | 11.038(10.832-11.247) | 11.692(11.033-12.389) | 12.384(11.229-13.658) | 13.118(11.427-15.059) | 13.895(11.628-16.604) | 30.30934 | 27.6253 |
| Both | Greenland | 14.729(14.331-15.138) | 14.923(13.713-16.239) | 15.119(13.106-17.441) | 15.317(12.524-18.734) | 15.519(11.967-20.124) | 25.73471 | 5.717301 |
| Male | Canada | 11.731(11.418-12.053) | 12.39(11.396-13.47) | 13.086(11.362-15.071) | 13.821(11.326-16.865) | 14.597(11.29-18.873) | 20.56136 | 26.11312 |
| Male | Greenland | 15.072(14.869-15.276) | 15.276(14.652-15.926) | 15.483(14.429-16.613) | 15.692(14.209-17.331) | 15.905(13.992-18.08) | 35.99648 | 5.858582 |
| Female | Canada | 10.435(10.225-10.648) | 11.057(10.387-11.769) | 11.716(10.542-13.02) | 12.414(10.697-14.406) | 13.154(10.855-15.939) | 39.7769 | 27.75728 |
| Female | Greenland | 14.242(13.648-14.861) | 14.455(12.674-16.486) | 14.671(11.748-18.322) | 14.891(10.887-20.367) | 15.114(10.089-22.642) | 17.58847 | 6.523001 |


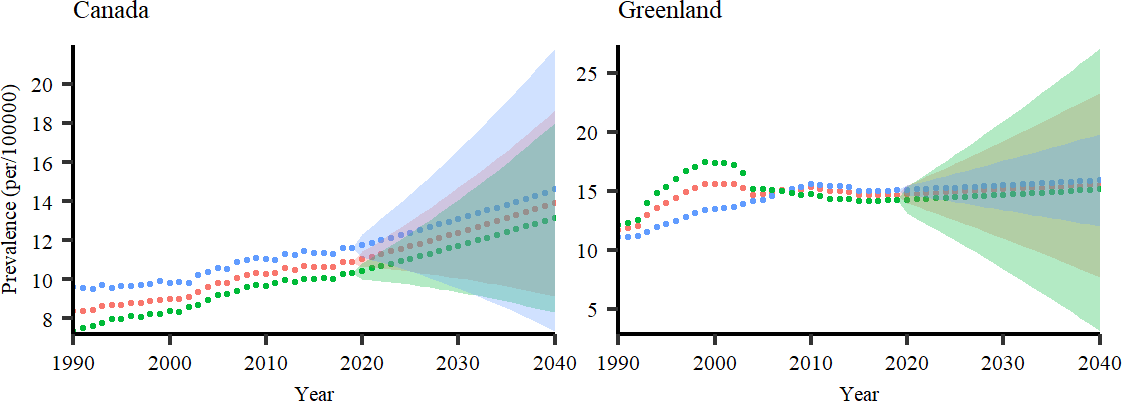


Supplemental Figure 24. Observed and projected age-standardized prevalence rate (ASPR) values from 1990 to 2040 for both sex (Red lines), females (Green lines), and men (Blue lines) in the High-income North America. The halo effect observed in each scatter plot accurately represents projections that extend across the temporal span from 2019 to 2040 with 95% confidence intervals.


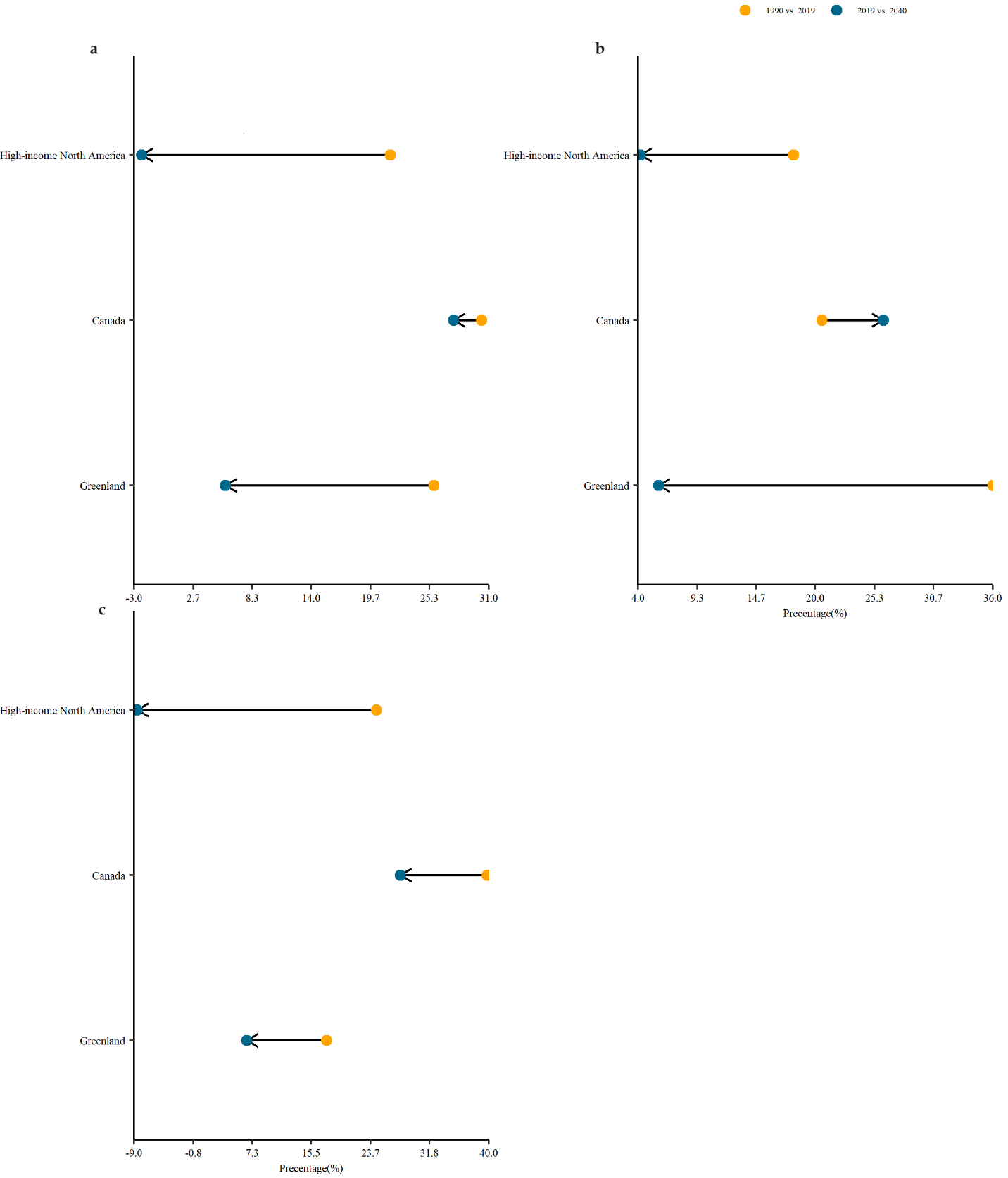


Supplemental Figure 25. The Lollipop plot between the two calculated percentage changes from 1990 to 2019 and 2019 to 2040 for both sexes (a), males (b), and females (c) in the High-income North America. Each line represents two time periods and show the change of ASPR increase or decrease during time.

# North Africa and Middle East

| Supplemental Table 13: Age-standardized prevalence rates (ASPR) from 2020 to 2040, and percentage changes for the time periods 1990 to 2019 and 2019 to 2040, for North Africa and Middle East. | | | | | | | | |
| --- | --- | --- | --- | --- | --- | --- | --- | --- |
| Group | Country | 2020 | 2025 | 2030 | 2035 | 2040 | 1990 vs. 2019 | 2019 vs. 2040 |
| Both | Afghanistan | 1.956(1.936-1.977) | 2.148(2.079-2.218) | 2.358(2.232-2.49) | 2.588(2.397-2.795) | 2.841(2.573-3.138) | 38.07837 | 48.21149 |
| Both | Algeria | 3.196(3.161-3.23) | 3.764(3.641-3.891) | 4.434(4.193-4.689) | 5.223(4.827-5.652) | 6.153(5.557-6.812) | 102.3834 | 98.9828 |
| Both | Bahrain | 5.58(5.132-6.066) | 6.741(5.208-8.725) | 8.145(5.267-12.596) | 9.841(5.324-18.191) | 11.89(5.38-26.276) | 17.6149 | 120.2579 |
| Both | Egypt | 3.418(3.243-3.602) | 3.629(3.085-4.267) | 3.852(2.929-5.067) | 4.09(2.78-6.018) | 4.342(2.638-7.148) | 122.0742 | 27.98025 |
| Both | Iran (Islamic Republic of) | 3.822(3.68-3.969) | 4.59(3.991-5.28) | 5.514(4.322-7.034) | 6.622(4.679-9.373) | 7.954(5.065-12.49) | 129.1919 | 115.9658 |
| Both | Iraq | 4.784(4.707-4.862) | 5.591(5.318-5.878) | 6.535(6.004-7.112) | 7.637(6.778-8.605) | 8.926(7.652-10.411) | 104.3915 | 92.37776 |
| Both | Jordan | 4.083(3.939-4.232) | 4.4(3.939-4.916) | 4.742(3.932-5.719) | 5.111(3.926-6.655) | 5.509(3.918-7.745) | 110.7554 | 37.14389 |
| Both | Kuwait | 4.316(3.897-4.779) | 5.018(3.663-6.874) | 5.834(3.428-9.931) | 6.784(3.206-14.355) | 7.888(2.998-20.755) | 40.78696 | 88.30326 |
| Both | Lebanon | 5.401(5.355-5.447) | 5.819(5.668-5.973) | 6.269(5.997-6.552) | 6.753(6.345-7.188) | 7.276(6.713-7.885) | 123.7525 | 36.6739 |
| Both | Libya | 5.439(5.286-5.597) | 5.624(5.15-6.143) | 5.816(5.011-6.751) | 6.014(4.875-7.42) | 6.219(4.742-8.156) | 88.07675 | 14.74881 |
| Both | Morocco | 2.945(2.897-2.994) | 3.426(3.256-3.605) | 3.986(3.657-4.344) | 4.637(4.107-5.235) | 5.395(4.613-6.309) | 104.1266 | 88.79993 |
| Both | Palestine | 5.087(4.915-5.264) | 5.143(4.627-5.717) | 5.201(4.35-6.218) | 5.259(4.088-6.765) | 5.318(3.842-7.36) | 107.2012 | 3.815707 |
| Both | Oman | 4.479(4.409-4.55) | 4.105(3.91-4.311) | 3.763(3.465-4.086) | 3.449(3.071-3.874) | 3.162(2.722-3.673) | 196.5841 | -31.1146 |
| Both | Qatar | 5.72(5.183-6.312) | 5.857(4.321-7.939) | 5.997(3.587-10.026) | 6.141(2.977-12.67) | 6.288(2.469-16.013) | 71.54517 | 10.32951 |
| Both | Saudi Arabia | 4.083(4.033-4.134) | 4.567(4.395-4.746) | 5.108(4.787-5.45) | 5.713(5.214-6.259) | 6.389(5.678-7.189) | 241.7129 | 59.83881 |
| Both | Sudan | 2.733(2.682-2.784) | 2.992(2.824-3.17) | 3.276(2.972-3.611) | 3.587(3.127-4.115) | 3.927(3.29-4.689) | 172.7007 | 45.61927 |
| Both | Syrian Arab Republic | 2.793(2.727-2.861) | 2.76(2.562-2.972) | 2.726(2.405-3.091) | 2.694(2.257-3.215) | 2.661(2.118-3.343) | 112.2732 | -4.98939 |
| Both | Tunisia | 3.011(2.969-3.055) | 3.342(3.198-3.493) | 3.709(3.443-3.997) | 4.117(3.706-4.574) | 4.569(3.989-5.234) | 99.76071 | 54.83588 |
| Both | Turkey | 6.501(6.403-6.6) | 7.073(6.75-7.411) | 7.695(7.11-8.327) | 8.371(7.489-9.358) | 9.108(7.888-10.516) | 66.00935 | 42.26653 |
| Both | United Arab Emirates | 13.625(13.041-14.235) | 16.178(14.133-18.519) | 19.21(15.288-24.139) | 22.81(16.533-31.471) | 27.085(17.879-41.033) | 123.086 | 107.0672 |
| Both | Yemen | 1.598(1.579-1.617) | 1.61(1.552-1.67) | 1.621(1.524-1.725) | 1.633(1.497-1.781) | 1.645(1.47-1.84) | 79.75534 | 3.202432 |
| Male | Afghanistan | 2.355(2.322-2.389) | 2.498(2.391-2.61) | 2.649(2.46-2.853) | 2.81(2.531-3.119) | 2.981(2.605-3.411) | 30.66655 | 28.27818 |
| Male | Algeria | 3.396(3.355-3.437) | 3.938(3.794-4.088) | 4.568(4.288-4.865) | 5.297(4.847-5.79) | 6.144(5.477-6.891) | 93.29617 | 86.27862 |
| Male | Bahrain | 6.259(5.698-6.875) | 7.714(5.772-10.307) | 9.506(5.824-15.515) | 11.715(5.874-23.365) | 14.437(5.922-35.193) | 14.28902 | 139.4009 |
| Male | Egypt | 4.099(3.883-4.326) | 4.383(3.709-5.18) | 4.687(3.535-6.216) | 5.013(3.368-7.461) | 5.361(3.208-8.957) | 109.135 | 32.05659 |
| Male | Iran (Islamic Republic of) | 4.51(4.369-4.656) | 5.324(4.9-5.784) | 6.284(5.489-7.195) | 7.418(6.147-8.953) | 8.757(6.883-11.141) | 121.0609 | 100.7591 |
| Male | Iraq | 5.87(5.779-5.962) | 6.906(6.581-7.247) | 8.126(7.49-8.816) | 9.56(8.523-10.724) | 11.249(9.699-13.046) | 93.31339 | 97.62922 |
| Male | Jordan | 4.416(4.285-4.551) | 4.772(4.348-5.237) | 5.156(4.407-6.033) | 5.572(4.465-6.952) | 6.02(4.524-8.011) | 107.866 | 38.70879 |
| Male | Kuwait | 4.913(4.285-5.633) | 5.805(3.805-8.856) | 6.859(3.359-14.002) | 8.104(2.964-22.158) | 9.575(2.614-35.072) | 64.31459 | 101.3316 |
| Male | Lebanon | 5.597(5.534-5.661) | 5.901(5.698-6.112) | 6.221(5.863-6.601) | 6.558(6.033-7.13) | 6.914(6.207-7.702) | 115.9472 | 24.58729 |
| Male | Libya | 6.633(6.449-6.823) | 6.892(6.319-7.518) | 7.162(6.183-8.295) | 7.442(6.05-9.153) | 7.732(5.919-10.1) | 77.8805 | 17.08406 |
| Male | Morocco | 3.462(3.373-3.553) | 4.03(3.718-4.367) | 4.69(4.094-5.372) | 5.459(4.508-6.611) | 6.354(4.963-8.134) | 87.29208 | 89.01439 |
| Male | Palestine | 5.899(5.64-6.171) | 5.725(4.983-6.577) | 5.555(4.394-7.023) | 5.39(3.873-7.502) | 5.231(3.414-8.014) | 88.86892 | -12.7764 |
| Male | Oman | 4.323(4.229-4.42) | 3.674(3.432-3.932) | 3.122(2.783-3.502) | 2.653(2.256-3.119) | 2.254(1.829-2.778) | 174.5264 | -50.0236 |
| Male | Qatar | 5.24(4.57-6.007) | 5.359(3.513-8.173) | 5.481(2.685-11.185) | 5.605(2.051-15.317) | 5.732(1.566-20.982) | 41.56327 | 9.551579 |
| Male | Saudi Arabia | 4.145(4.092-4.2) | 4.589(4.407-4.777) | 5.079(4.745-5.437) | 5.622(5.107-6.189) | 6.223(5.498-7.045) | 206.8725 | 52.92551 |
| Male | Sudan | 3.396(3.311-3.483) | 3.598(3.327-3.89) | 3.811(3.34-4.349) | 4.037(3.352-4.862) | 4.277(3.364-5.437) | 173.5143 | 26.43828 |
| Male | Syrian Arab Republic | 3.077(2.994-3.161) | 2.938(2.702-3.195) | 2.806(2.436-3.232) | 2.68(2.195-3.271) | 2.559(1.978-3.31) | 106.7965 | -17.7148 |
| Male | Tunisia | 3.747(3.692-3.802) | 4.162(3.979-4.354) | 4.625(4.286-4.99) | 5.138(4.615-5.72) | 5.708(4.97-6.556) | 95.20173 | 55.48745 |
| Male | Turkey | 7.982(7.793-8.176) | 8.496(7.889-9.149) | 9.042(7.979-10.248) | 9.624(8.068-11.48) | 10.243(8.158-12.862) | 60.88014 | 29.5874 |
| Male | United Arab Emirates | 14.638(14.126-15.168) | 16.35(14.648-18.25) | 18.263(15.166-21.991) | 20.399(15.7-26.504) | 22.785(16.252-31.945) | 111.2261 | 58.8592 |
| Male | Yemen | 1.902(1.881-1.923) | 1.919(1.856-1.986) | 1.937(1.83-2.051) | 1.955(1.804-2.12) | 1.974(1.778-2.19) | 55.38791 | 4.105445 |
| Female | Afghanistan | 1.6(1.586-1.614) | 1.83(1.781-1.88) | 2.092(1.998-2.19) | 2.392(2.242-2.551) | 2.734(2.516-2.972) | 61.94757 | 75.85778 |
| Female | Algeria | 2.999(2.968-3.031) | 3.601(3.485-3.72) | 4.323(4.09-4.569) | 5.19(4.801-5.61) | 6.23(5.634-6.89) | 113.113 | 115.6531 |
| Female | Bahrain | 4.789(4.409-5.201) | 5.457(4.228-7.043) | 6.218(4.04-9.57) | 7.086(3.859-13.009) | 8.074(3.686-17.687) | 20.1216 | 72.24939 |
| Female | Egypt | 2.694(2.549-2.847) | 2.824(2.382-3.348) | 2.961(2.221-3.948) | 3.105(2.07-4.656) | 3.255(1.929-5.492) | 142.9318 | 21.27107 |
| Female | Iran (Islamic Republic of) | 3.115(2.982-3.254) | 3.707(3.154-4.357) | 4.411(3.331-5.843) | 5.249(3.516-7.837) | 6.247(3.712-10.513) | 145.944 | 107.6507 |
| Female | Iraq | 3.762(3.692-3.833) | 4.374(4.128-4.634) | 5.084(4.61-5.607) | 5.91(5.149-6.784) | 6.871(5.751-8.209) | 124.0613 | 88.40837 |
| Female | Jordan | 3.709(3.533-3.893) | 3.963(3.412-4.603) | 4.235(3.289-5.454) | 4.526(3.169-6.464) | 4.837(3.053-7.662) | 112.3523 | 32.22875 |
| Female | Kuwait | 3.399(3.153-3.664) | 3.847(3.05-4.851) | 4.353(2.941-6.443) | 4.927(2.835-8.561) | 5.576(2.733-11.377) | 7.614265 | 67.86665 |
| Female | Lebanon | 5.239(5.188-5.29) | 5.755(5.585-5.932) | 6.323(6.009-6.653) | 6.946(6.465-7.463) | 7.631(6.956-8.371) | 134.3899 | 48.54822 |
| Female | Libya | 4.199(4.054-4.35) | 4.319(3.874-4.814) | 4.442(3.697-5.336) | 4.568(3.527-5.916) | 4.698(3.365-6.559) | 116.6723 | 12.22147 |
| Female | Morocco | 2.436(2.403-2.47) | 2.839(2.72-2.963) | 3.309(3.078-3.557) | 3.856(3.483-4.27) | 4.494(3.941-5.126) | 132.554 | 90.35985 |
| Female | Palestine | 4.382(4.268-4.499) | 4.644(4.281-5.038) | 4.923(4.29-5.649) | 5.218(4.298-6.334) | 5.53(4.306-7.103) | 127.9815 | 26.69027 |
| Female | Oman | 4.704(4.614-4.796) | 4.611(4.343-4.895) | 4.519(4.084-5) | 4.43(3.841-5.108) | 4.342(3.612-5.219) | 226.0516 | -8.45405 |
| Female | Qatar | 7.149(6.749-7.573) | 7.579(6.345-9.053) | 8.034(5.95-10.848) | 8.516(5.578-13.003) | 9.028(5.228-15.589) | 141.0092 | 27.97037 |
| Female | Saudi Arabia | 3.985(3.929-4.042) | 4.52(4.326-4.723) | 5.126(4.76-5.521) | 5.814(5.237-6.455) | 6.595(5.762-7.547) | 306.5034 | 69.72556 |
| Female | Sudan | 1.947(1.932-1.962) | 2.304(2.251-2.359) | 2.727(2.621-2.837) | 3.227(3.052-3.412) | 3.819(3.554-4.105) | 162.4547 | 103.0512 |
| Female | Syrian Arab Republic | 2.535(2.465-2.608) | 2.628(2.408-2.867) | 2.724(2.35-3.156) | 2.823(2.294-3.474) | 2.926(2.238-3.825) | 124.9412 | 16.39809 |
| Female | Tunisia | 2.323(2.282-2.364) | 2.583(2.445-2.728) | 2.872(2.617-3.15) | 3.193(2.802-3.639) | 3.55(3-4.202) | 115.8626 | 56.08514 |
| Female | Turkey | 5.109(5.052-5.167) | 5.731(5.536-5.933) | 6.428(6.062-6.816) | 7.21(6.639-7.831) | 8.087(7.27-8.997) | 75.0068 | 61.98235 |
| Female | United Arab Emirates | 9.378(8.277-10.626) | 9.365(6.921-12.674) | 9.352(5.757-15.194) | 9.34(4.784-18.232) | 9.327(3.975-21.885) | 121.0952 | -3.55379 |
| Female | Yemen | 1.307(1.288-1.325) | 1.321(1.263-1.38) | 1.335(1.238-1.438) | 1.349(1.214-1.499) | 1.363(1.19-1.562) | 120.4811 | 4.699083 |


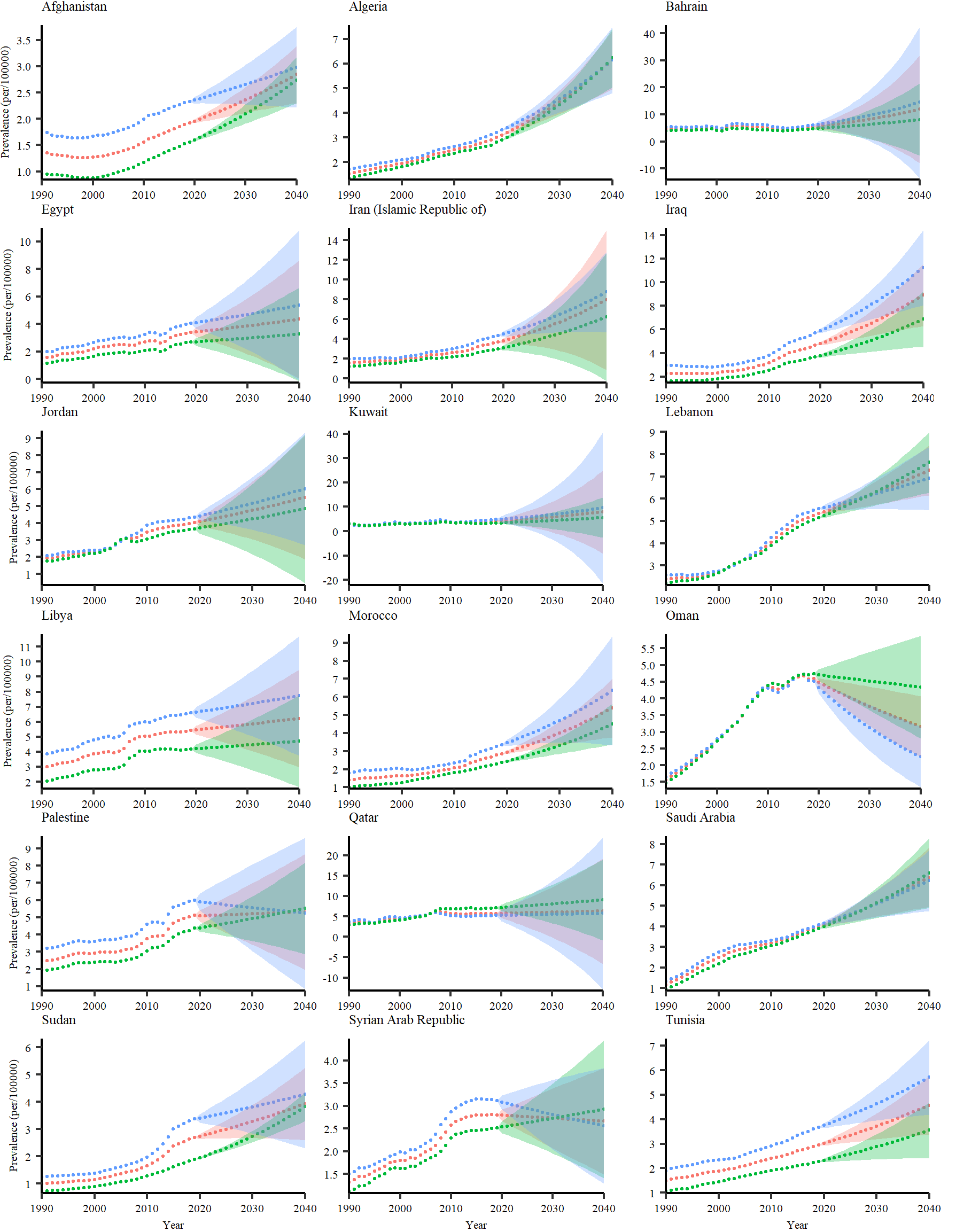


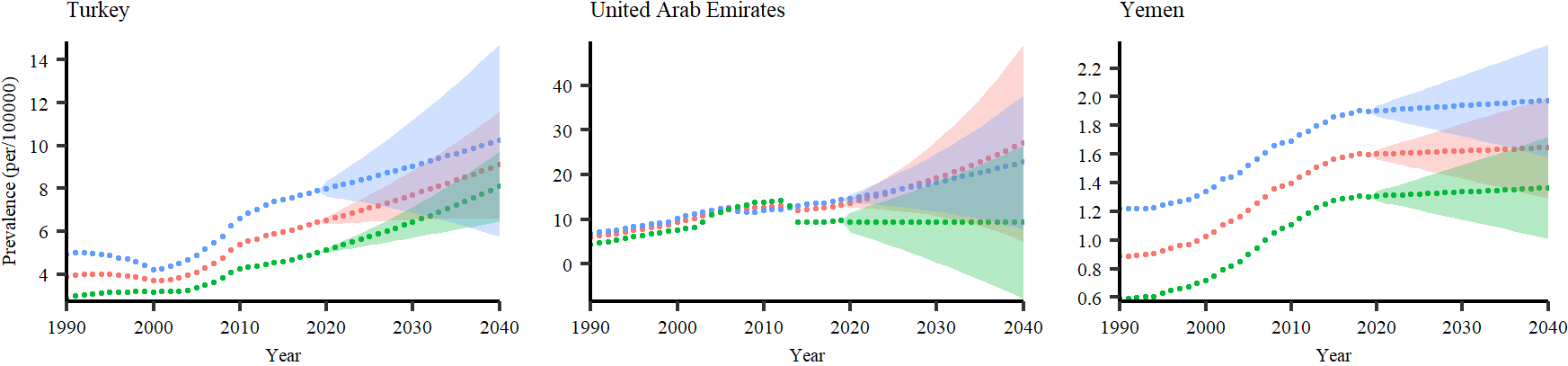


Supplemental Figure 26. Observed and projected age-standardized prevalence rate (ASPR) values from 1990 to 2040 for both sex (Red lines), females (Green lines), and men (Blue lines) in the North Africa and Middle East. The halo effect observed in each scatter plot accurately represents projections that extend across the temporal span from 2019 to 2040 with 95% confidence intervals.


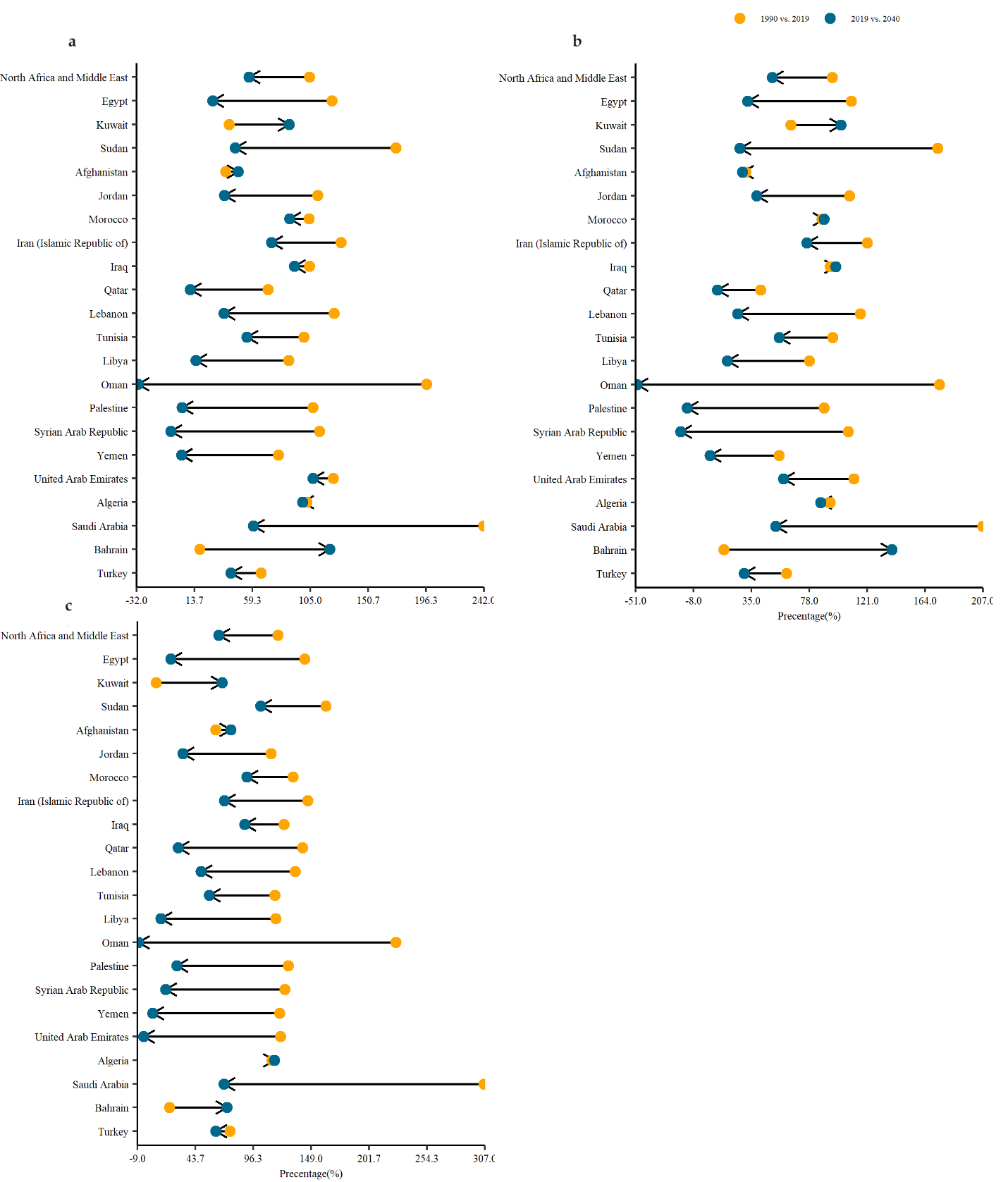


Supplemental Figure 27. The Lollipop plot between the two calculated percentage changes from 1990 to 2019 and 2019 to 2040 for both sexes (a), males (b), and females (c) in the North Africa and Middle East. Each line represents two time periods and show the change of ASPR increase or decrease during time.

# 
