# Supplementary material for: Global Trend in Pancreatic Cancer Prevalence Rates Through 2040: An Illness‐Death Modeling Study
Source: Cancer Med. 2024 Oct 23;13(20):e70318. doi: 10.1002/cam4.70318 (PMC11497012; doi:10.1002/cam4.70318)
Supplement: Supplementary file 4 — Data S4. [file CAM4-13-e70318-s004.docx]

#

# Oceania

| Supplemental Table 14: Age-standardized prevalence rates (ASPR) from 2020 to 2040, and percentage changes for the time periods 1990 to 2019 and 2019 to 2040, for Oceania. | | | | | | | | |
| --- | --- | --- | --- | --- | --- | --- | --- | --- |
| Group | Country | 2020 | 2025 | 2030 | 2035 | 2040 | 1990 vs. 2019 | 2019 vs. 2040 |
| Both | American Samoa | 3.687(3.556-3.824) | 3.528(3.155-3.945) | 3.375(2.794-4.077) | 3.229(2.474-4.215) | 3.089(2.191-4.357) | 63.314 | -17.1418 |
| Both | Micronesia (Federated States of) | 3.857(3.826-3.889) | 4.107(4.006-4.211) | 4.373(4.193-4.562) | 4.657(4.388-4.942) | 4.959(4.593-5.354) | 66.44955 | 30.29005 |
| Both | Fiji | 3.085(2.993-3.18) | 3.452(3.144-3.791) | 3.864(3.299-4.526) | 4.324(3.461-5.403) | 4.839(3.63-6.452) | 58.0335 | 60.61239 |
| Both | Guam | 4.063(3.908-4.224) | 4.35(3.858-4.905) | 4.658(3.803-5.705) | 4.988(3.748-6.638) | 5.34(3.693-7.723) | 34.58384 | 32.90136 |
| Both | Kiribati | 2.081(2.046-2.118) | 2.388(2.264-2.519) | 2.74(2.504-2.998) | 3.144(2.769-3.569) | 3.607(3.062-4.25) | 25.29254 | 78.99387 |
| Both | Marshall Islands | 2.88(2.805-2.957) | 3.077(2.837-3.337) | 3.287(2.866-3.77) | 3.511(2.894-4.259) | 3.751(2.923-4.813) | 68.9443 | 32.19669 |
| Both | Northern Mariana Islands | 5.791(5.555-6.037) | 5.708(5.02-6.49) | 5.626(4.528-6.989) | 5.545(4.084-7.529) | 5.465(3.683-8.11) | 125.5702 | -6.03505 |
| Both | Papua New Guinea | 1.286(1.273-1.298) | 1.391(1.35-1.433) | 1.505(1.431-1.583) | 1.629(1.516-1.749) | 1.762(1.607-1.932) | 44.7917 | 39.42655 |
| Both | Samoa | 3.294(3.225-3.363) | 3.634(3.406-3.877) | 4.009(3.593-4.473) | 4.423(3.791-5.162) | 4.88(3.999-5.956) | 5.660083 | 51.42495 |
| Both | Solomon Islands | 2.325(2.27-2.382) | 2.521(2.341-2.714) | 2.733(2.412-3.096) | 2.963(2.485-3.533) | 3.212(2.56-4.031) | 66.62026 | 40.42637 |
| Both | Tonga | 3.234(3.109-3.365) | 3.453(3.056-3.902) | 3.687(2.999-4.532) | 3.937(2.943-5.266) | 4.203(2.888-6.119) | 55.02927 | 31.82823 |
| Both | Vanuatu | 2.231(2.17-2.293) | 2.409(2.213-2.623) | 2.601(2.253-3.004) | 2.809(2.294-3.44) | 3.033(2.335-3.94) | 66.80662 | 38.32901 |
| Male | American Samoa | 4.846(4.629-5.073) | 4.55(3.95-5.242) | 4.273(3.364-5.426) | 4.012(2.864-5.619) | 3.767(2.439-5.818) | 65.61572 | -23.4581 |
| Male | Micronesia (Federated States of) | 4.304(4.271-4.338) | 4.555(4.446-4.667) | 4.82(4.627-5.021) | 5.101(4.815-5.404) | 5.398(5.011-5.815) | 63.10216 | 26.9259 |
| Male | Fiji | 3.999(3.853-4.151) | 4.557(4.062-5.113) | 5.194(4.277-6.308) | 5.919(4.501-7.784) | 6.746(4.737-9.606) | 54.02081 | 73.55684 |
| Male | Guam | 4.707(4.338-5.108) | 4.927(3.829-6.341) | 5.157(3.368-7.898) | 5.398(2.961-9.843) | 5.651(2.602-12.269) | 42.55191 | 20.99688 |
| Male | Kiribati | 2.202(2.163-2.241) | 2.449(2.319-2.587) | 2.725(2.484-2.989) | 3.032(2.661-3.454) | 3.373(2.85-3.991) | 24.37845 | 57.11162 |
| Male | Marshall Islands | 3.122(3.002-3.246) | 3.334(2.956-3.761) | 3.561(2.905-4.365) | 3.803(2.855-5.067) | 4.062(2.805-5.882) | 53.09896 | 32.03301 |
| Male | Northern Mariana Islands | 5.698(5.542-5.857) | 5.448(5.002-5.933) | 5.209(4.51-6.016) | 4.98(4.065-6.102) | 4.762(3.664-6.189) | 127.7023 | -17.2354 |
| Male | Papua New Guinea | 1.501(1.487-1.515) | 1.615(1.57-1.661) | 1.737(1.656-1.822) | 1.868(1.747-1.998) | 2.01(1.843-2.192) | 41.40846 | 36.0554 |
| Male | Samoa | 3.856(3.779-3.934) | 4.184(3.932-4.453) | 4.54(4.087-5.044) | 4.927(4.248-5.714) | 5.347(4.416-6.474) | -1.0504 | 41.22288 |
| Male | Solomon Islands | 2.778(2.717-2.841) | 2.953(2.757-3.164) | 3.139(2.794-3.526) | 3.336(2.832-3.931) | 3.546(2.87-4.382) | 63.61764 | 29.20276 |
| Male | Tonga | 4.58(4.361-4.81) | 4.812(4.137-5.598) | 5.056(3.916-6.528) | 5.313(3.706-7.616) | 5.582(3.507-8.885) | 62.55793 | 23.20107 |
| Male | Vanuatu | 2.559(2.502-2.617) | 2.725(2.542-2.922) | 2.902(2.58-3.264) | 3.09(2.618-3.648) | 3.291(2.656-4.076) | 66.40257 | 30.44436 |
| Female | American Samoa | 2.597(2.51-2.688) | 2.595(2.335-2.885) | 2.593(2.169-3.1) | 2.591(2.014-3.333) | 2.589(1.87-3.583) | 65.85772 | -0.37581 |
| Female | Micronesia (Federated States of) | 3.427(3.397-3.457) | 3.672(3.574-3.773) | 3.936(3.76-4.119) | 4.218(3.955-4.498) | 4.52(4.16-4.912) | 72.30655 | 33.87689 |
| Female | Fiji | 2.306(2.239-2.375) | 2.529(2.309-2.77) | 2.774(2.379-3.236) | 3.043(2.45-3.78) | 3.339(2.524-4.417) | 73.36161 | 47.46671 |
| Female | Guam | 3.402(3.197-3.62) | 3.713(3.065-4.498) | 4.052(2.931-5.604) | 4.423(2.801-6.984) | 4.828(2.677-8.706) | 26.45233 | 43.86525 |
| Female | Kiribati | 1.998(1.963-2.034) | 2.344(2.219-2.477) | 2.75(2.506-3.018) | 3.226(2.83-3.678) | 3.785(3.196-4.482) | 26.6326 | 96.65361 |
| Female | Marshall Islands | 2.636(2.6-2.672) | 2.835(2.718-2.957) | 3.049(2.839-3.275) | 3.279(2.965-3.626) | 3.527(3.097-4.016) | 90.41814 | 36.03617 |
| Female | Northern Mariana Islands | 5.862(5.481-6.27) | 5.87(4.769-7.224) | 5.877(4.139-8.347) | 5.885(3.59-9.648) | 5.893(3.113-11.153) | 124.4265 | 0.471962 |
| Female | Papua New Guinea | 1.043(1.032-1.054) | 1.143(1.106-1.18) | 1.252(1.186-1.322) | 1.372(1.27-1.481) | 1.503(1.361-1.659) | 48.27691 | 46.99559 |
| Female | Samoa | 2.735(2.673-2.799) | 3.075(2.864-3.302) | 3.458(3.066-3.9) | 3.888(3.282-4.606) | 4.372(3.514-5.441) | 16.17596 | 64.04185 |
| Female | Solomon Islands | 1.855(1.8-1.91) | 2.089(1.907-2.289) | 2.354(2.017-2.747) | 2.651(2.133-3.296) | 2.987(2.255-3.956) | 83.24439 | 65.07622 |
| Female | Tonga | 2.044(2.001-2.087) | 2.267(2.125-2.419) | 2.515(2.254-2.805) | 2.789(2.391-3.254) | 3.094(2.536-3.776) | 46.59192 | 54.89694 |
| Female | Vanuatu | 1.868(1.803-1.935) | 2.053(1.84-2.291) | 2.257(1.875-2.716) | 2.481(1.91-3.221) | 2.727(1.946-3.82) | 71.15002 | 49.17593 |


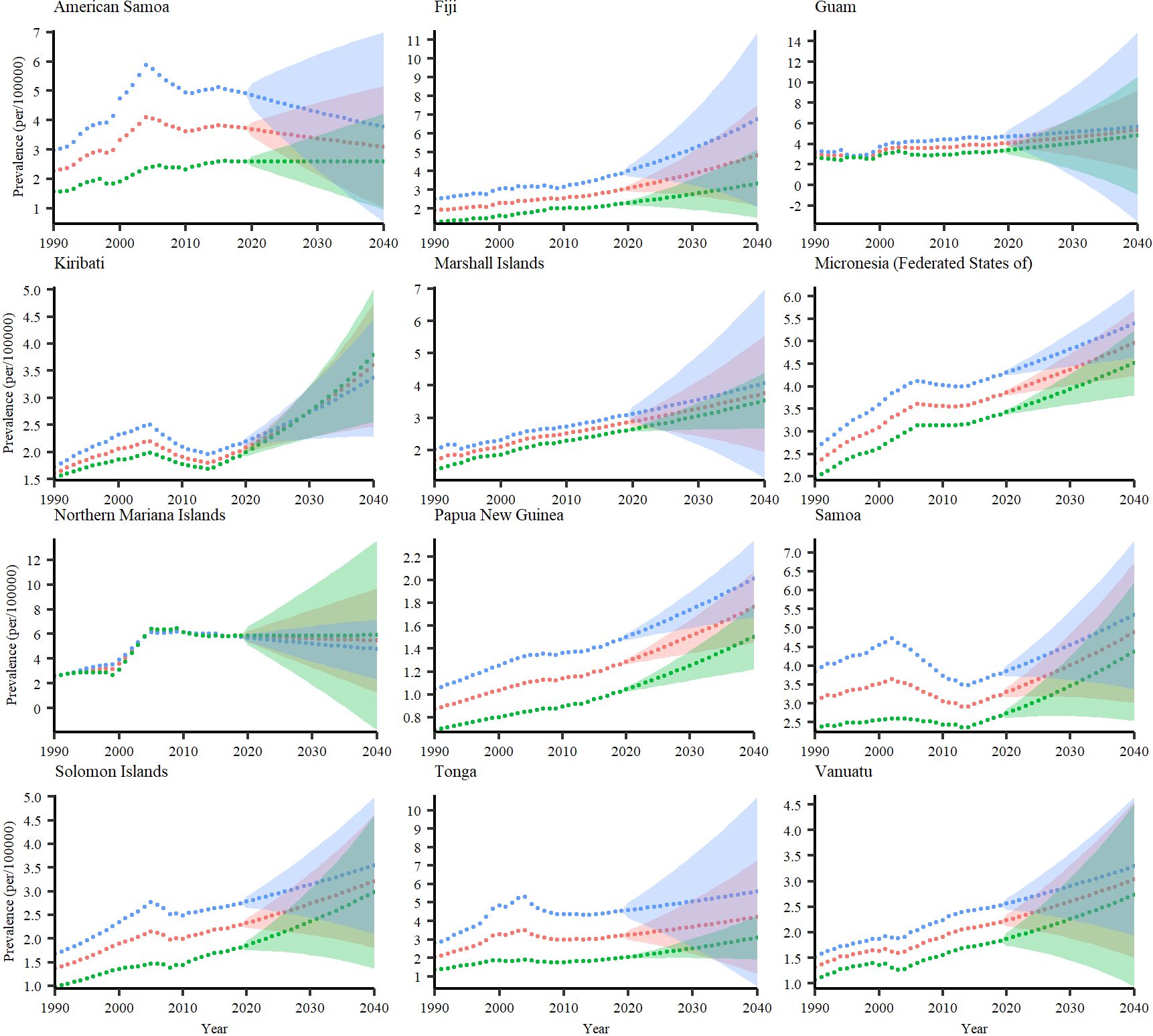


Supplemental Figure 28. Observed and projected age-standardized prevalence rate (ASPR) values from 1990 to 2040 for both sex (Red lines), females (Green lines), and men (Blue lines) in the Oceania. The halo effect observed in each scatter plot accurately represents projections that extend across the temporal span from 2019 to 2040 with 95% confidence intervals.


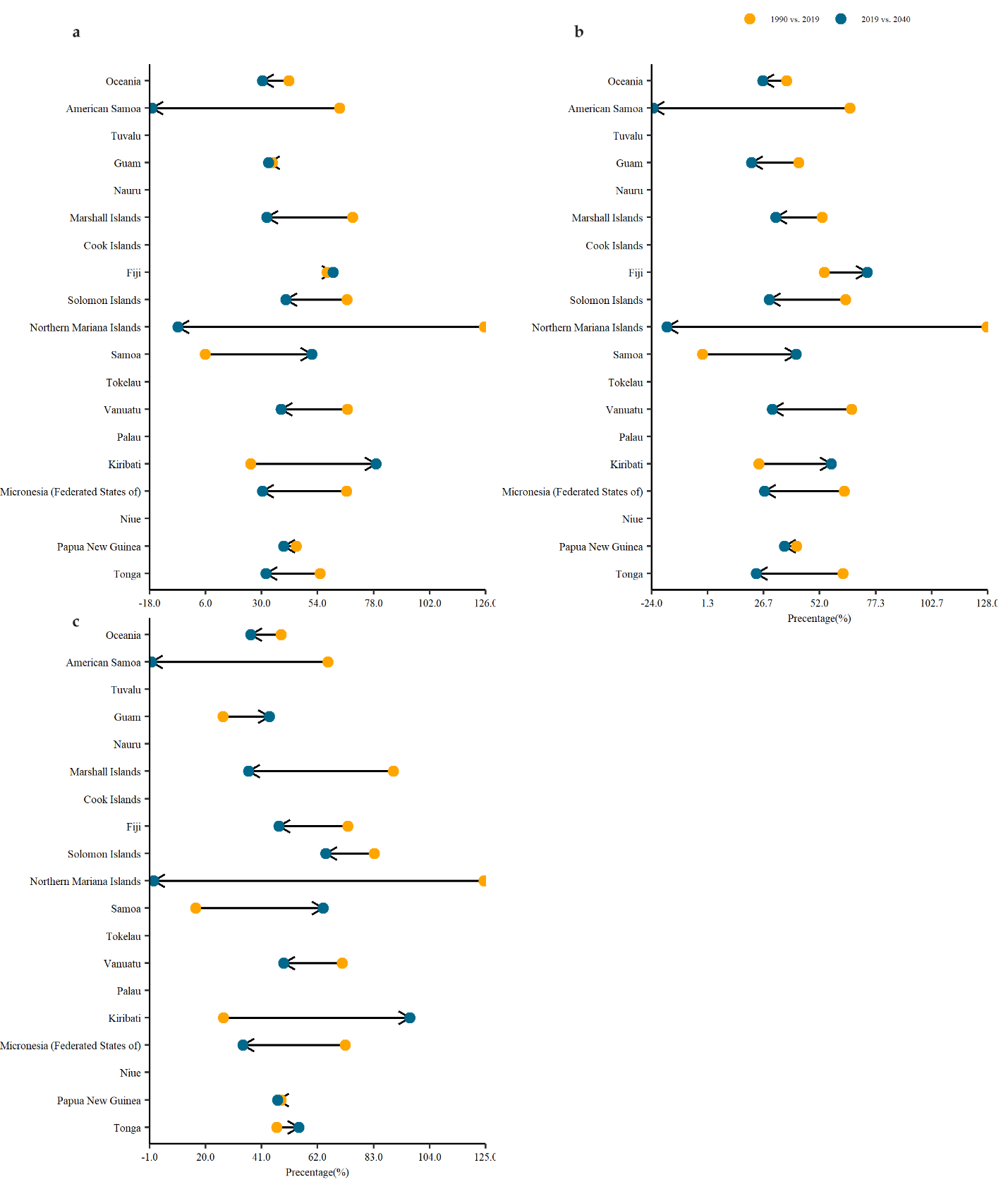


Supplemental Figure 29. The Lollipop plot between the two calculated percentage changes from 1990 to 2019 and 2019 to 2040 for both sexes (a), males (b), and females (c) in the Oceania. Each line represents two time periods and show the change of ASPR increase or decrease during time.

# South Asia

| Supplemental Table 15: Age-standardized prevalence rates (ASPR) from 2020 to 2040, and percentage changes for the time periods 1990 to 2019 and 2019 to 2040, for South Asia. | | | | | | | | |
| --- | --- | --- | --- | --- | --- | --- | --- | --- |
| Group | Country | 2020 | 2025 | 2030 | 2035 | 2040 | 1990 vs. 2019 | 2019 vs. 2040 |
| Both | Bangladesh | 1.589(1.543-1.636) | 1.887(1.724-2.064) | 2.24(1.924-2.608) | 2.66(2.147-3.296) | 3.159(2.396-4.165) | 60.32962 | 106.4193 |
| Both | Bhutan | 2.297(2.269-2.326) | 2.627(2.529-2.729) | 3.004(2.818-3.203) | 3.436(3.139-3.761) | 3.929(3.496-4.415) | 126.7231 | 75.52474 |
| Both | India | 2.231(2.152-2.312) | 2.529(2.264-2.825) | 2.868(2.379-3.457) | 3.251(2.498-4.231) | 3.686(2.624-5.179) | 108.1834 | 68.97918 |
| Both | Nepal | 2.057(2.03-2.085) | 2.371(2.276-2.471) | 2.733(2.549-2.931) | 3.15(2.856-3.475) | 3.631(3.199-4.122) | 164.5933 | 81.54361 |
| Both | Pakistan | 2.161(2.116-2.207) | 2.317(2.171-2.473) | 2.484(2.225-2.774) | 2.664(2.281-3.111) | 2.856(2.338-3.489) | 99.76036 | 33.80647 |
| Male | Bangladesh | 1.803(1.748-1.859) | 2.11(1.919-2.32) | 2.47(2.104-2.9) | 2.891(2.306-3.625) | 3.384(2.527-4.532) | 44.69466 | 94.33506 |
| Male | Bhutan | 2.523(2.485-2.562) | 2.876(2.743-3.014) | 3.278(3.027-3.549) | 3.736(3.34-4.179) | 4.258(3.685-4.92) | 98.60709 | 73.07252 |
| Male | India | 2.278(2.201-2.358) | 2.582(2.32-2.872) | 2.926(2.443-3.504) | 3.316(2.572-4.275) | 3.758(2.707-5.217) | 82.11049 | 68.966 |
| Male | Nepal | 2.326(2.282-2.37) | 2.664(2.514-2.824) | 3.053(2.767-3.368) | 3.497(3.045-4.017) | 4.007(3.351-4.791) | 158.7867 | 77.09628 |
| Male | Pakistan | 2.284(2.239-2.33) | 2.391(2.249-2.542) | 2.502(2.256-2.775) | 2.619(2.263-3.03) | 2.741(2.27-3.309) | 80.74943 | 20.9075 |
| Female | Bangladesh | 1.362(1.319-1.407) | 1.668(1.509-1.844) | 2.043(1.724-2.42) | 2.502(1.97-3.176) | 3.063(2.251-4.169) | 100.5817 | 134.7934 |
| Female | Bhutan | 2.06(2.04-2.08) | 2.373(2.302-2.446) | 2.734(2.597-2.878) | 3.149(2.929-3.386) | 3.628(3.304-3.983) | 166.9342 | 81.11345 |
| Female | India | 2.181(2.097-2.269) | 2.471(2.187-2.792) | 2.799(2.278-3.441) | 3.171(2.371-4.241) | 3.592(2.469-5.228) | 148.1108 | 68.09438 |
| Female | Nepal | 1.815(1.8-1.83) | 2.12(2.066-2.175) | 2.476(2.371-2.586) | 2.892(2.721-3.074) | 3.378(3.122-3.654) | 178.734 | 91.88929 |
| Female | Pakistan | 2.027(1.98-2.076) | 2.24(2.081-2.411) | 2.474(2.185-2.802) | 2.733(2.294-3.256) | 3.02(2.409-3.785) | 137.5756 | 51.74118 |


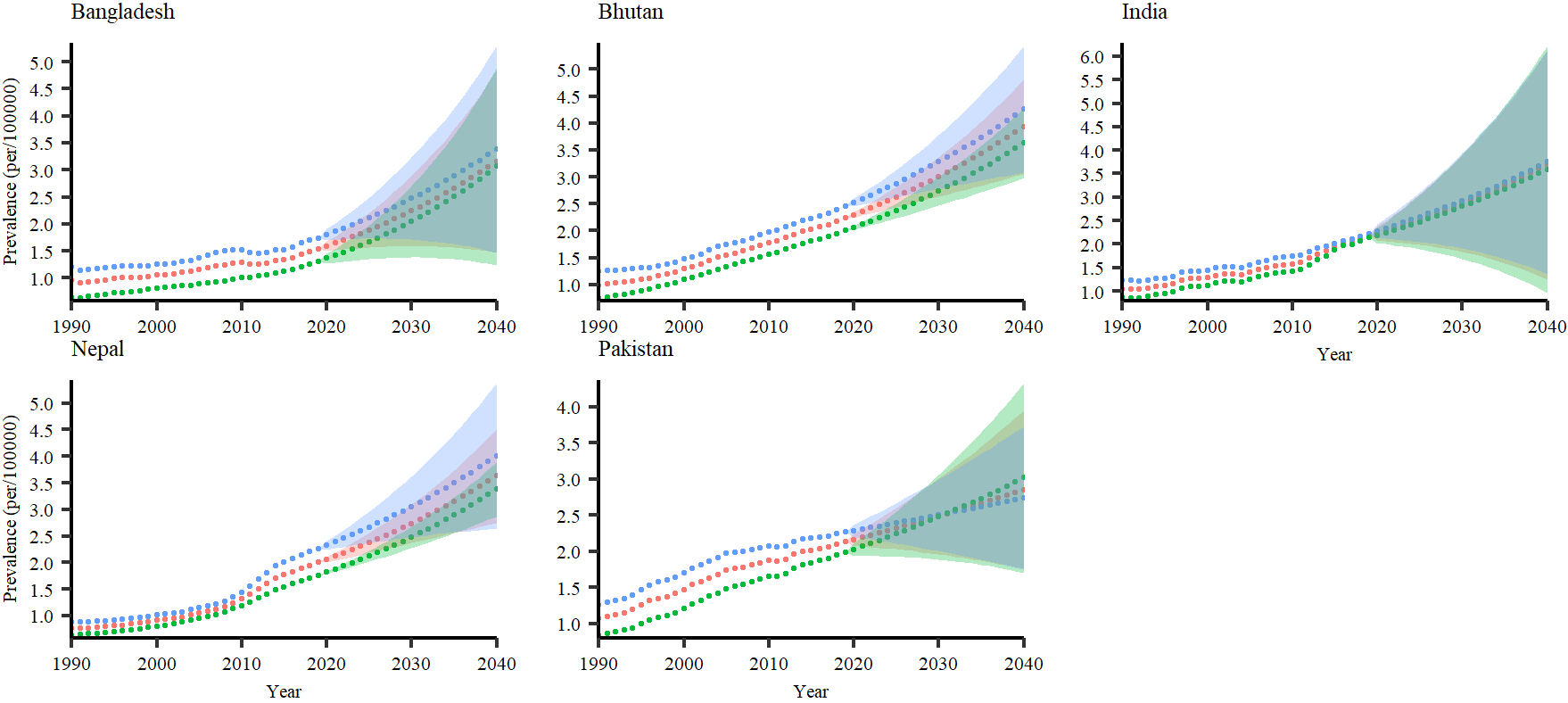


Supplemental Figure 30. Observed and projected age-standardized prevalence rate (ASPR) values from 1990 to 2040 for both sex (Red lines), females (Green lines), and men (Blue lines) in the South Asia. The halo effect observed in each scatter plot accurately represents projections that extend across the temporal span from 2019 to 2040 with 95% confidence intervals.


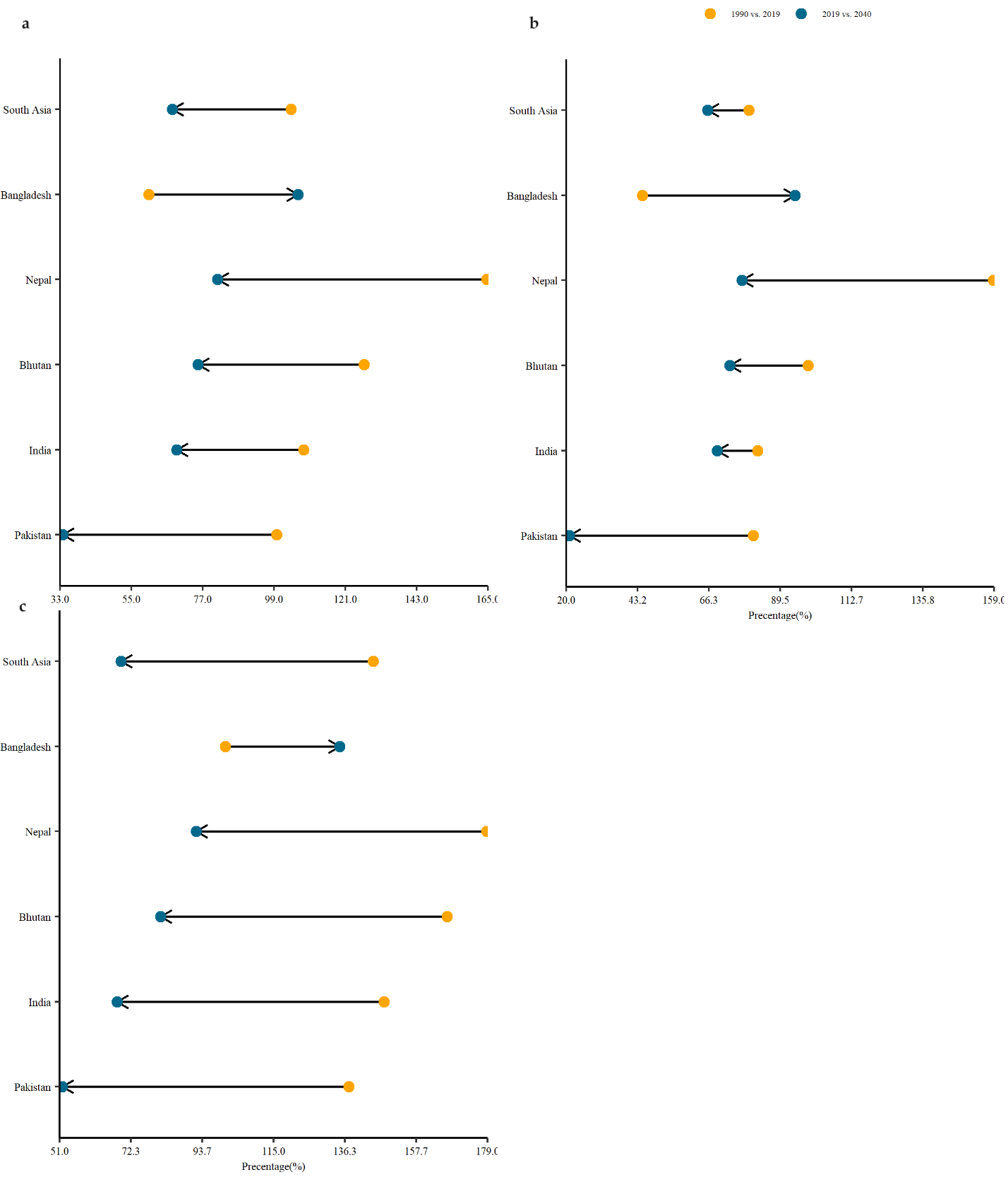


Supplemental Figure 31. The Lollipop plot between the two calculated percentage changes from 1990 to 2019 and 2019 to 2040 for both sexes (a), males (b), and females (c) in the South Asia. Each line represents two time periods and show the change of ASPR increase or decrease during time.

# Southeast Asia

| Supplemental Table 16: Age-standardized prevalence rates (ASPR) from 2020 to 2040, and percentage changes for the time periods 1990 to 2019 and 2019 to 2040, for Southeast Asia. | | | | | | | | |
| --- | --- | --- | --- | --- | --- | --- | --- | --- |
| Group | Country | 2020 | 2025 | 2030 | 2035 | 2040 | 1990 vs. 2019 | 2019 vs. 2040 |
| Both | Cambodia | 2.701(2.688-2.714) | 3.085(3.041-3.131) | 3.524(3.438-3.612) | 4.025(3.888-4.168) | 4.598(4.396-4.809) | 68.51553 | 74.85483 |
| Both | Indonesia | 3.834(3.816-3.853) | 4.387(4.323-4.452) | 5.02(4.897-5.146) | 5.744(5.546-5.948) | 6.572(6.281-6.876) | 125.2933 | 76.34076 |
| Both | Lao People's Democratic Republic | 1.441(1.235-1.681) | 0.835(0.518-1.345) | 0.484(0.216-1.082) | 0.28(0.09-0.872) | 0.162(0.038-0.703) | -0.73572 | -90.5727 |
| Both | Malaysia | 2.75(2.606-2.903) | 2.889(2.446-3.412) | 3.035(2.291-4.02) | 3.188(2.145-4.738) | 3.348(2.008-5.584) | 102.3115 | 22.3671 |
| Both | Maldives | 3.33(3.222-3.442) | 3.932(3.55-4.355) | 4.643(3.906-5.519) | 5.482(4.298-6.993) | 6.473(4.728-8.863) | 54.8711 | 101.0993 |
| Both | Mauritius | 4.122(3.888-4.371) | 4.347(3.628-5.209) | 4.584(3.377-6.222) | 4.833(3.142-7.435) | 5.097(2.924-8.885) | 30.3925 | 25.10388 |
| Both | Myanmar | 2.824(2.805-2.843) | 3.139(3.075-3.206) | 3.49(3.369-3.616) | 3.881(3.692-4.079) | 4.314(4.046-4.601) | 61.94167 | 56.18342 |
| Both | Philippines | 3.224(3.139-3.313) | 3.47(3.193-3.772) | 3.735(3.245-4.3) | 4.02(3.297-4.902) | 4.327(3.349-5.589) | 32.29901 | 36.20025 |
| Both | Sri Lanka | 2.223(2.172-2.276) | 2.328(2.165-2.502) | 2.437(2.157-2.753) | 2.551(2.148-3.029) | 2.671(2.139-3.334) | 73.78973 | 21.10316 |
| Both | Seychelles | 5.672(5.537-5.811) | 6.59(6.115-7.101) | 7.655(6.747-8.686) | 8.894(7.444-10.626) | 10.332(8.211-13) | 40.75501 | 87.86951 |
| Both | Thailand | 3.183(3.027-3.347) | 3.675(3.147-4.29) | 4.242(3.265-5.511) | 4.898(3.387-7.082) | 5.654(3.512-9.102) | 28.98672 | 82.81658 |
| Both | Timor-Leste | 2.148(2.085-2.213) | 2.267(2.068-2.486) | 2.393(2.049-2.795) | 2.526(2.029-3.144) | 2.666(2.01-3.537) | 82.23431 | 25.55124 |
| Both | Viet Nam | 3.321(3.297-3.345) | 3.66(3.58-3.742) | 4.034(3.886-4.188) | 4.446(4.217-4.687) | 4.9(4.578-5.245) | 158.7573 | 50.39023 |
| Male | Cambodia | 3.17(3.153-3.187) | 3.548(3.489-3.608) | 3.972(3.861-4.086) | 4.446(4.272-4.626) | 4.976(4.727-5.239) | 49.09998 | 60.59667 |
| Male | Indonesia | 3.748(3.731-3.765) | 4.3(4.241-4.36) | 4.934(4.82-5.051) | 5.661(5.477-5.851) | 6.495(6.224-6.778) | 113.8149 | 78.3212 |
| Male | Lao People's Democratic Republic | 1.898(1.738-2.073) | 1.348(1.027-1.769) | 0.957(0.604-1.515) | 0.68(0.356-1.299) | 0.483(0.209-1.113) | 0.110848 | -77.1246 |
| Male | Malaysia | 3.047(2.883-3.221) | 3.252(2.741-3.859) | 3.471(2.599-4.635) | 3.704(2.464-5.568) | 3.953(2.336-6.69) | 93.19394 | 30.74709 |
| Male | Maldives | 2.988(2.929-3.048) | 3.512(3.303-3.734) | 4.127(3.721-4.578) | 4.851(4.191-5.613) | 5.701(4.721-6.883) | 39.07209 | 97.19544 |
| Male | Mauritius | 4.906(4.496-5.352) | 4.833(3.693-6.323) | 4.761(3.023-7.499) | 4.69(2.472-8.898) | 4.621(2.022-10.559) | 31.49222 | -6.85609 |
| Male | Myanmar | 2.899(2.877-2.921) | 3.174(3.101-3.249) | 3.475(3.341-3.615) | 3.805(3.599-4.023) | 4.166(3.877-4.477) | 40.03571 | 46.50048 |
| Male | Philippines | 3.512(3.425-3.601) | 3.689(3.415-3.986) | 3.876(3.401-4.417) | 4.072(3.387-4.895) | 4.277(3.373-5.425) | 29.79524 | 22.94255 |
| Male | Sri Lanka | 2.229(2.161-2.3) | 2.275(2.066-2.506) | 2.323(1.974-2.733) | 2.371(1.885-2.982) | 2.42(1.8-3.253) | 51.77656 | 8.925768 |
| Male | Seychelles | 7.427(7.187-7.674) | 8.667(7.832-9.591) | 10.114(8.523-12.003) | 11.804(9.273-15.024) | 13.775(10.089-18.807) | 29.1211 | 91.51289 |
| Male | Thailand | 3.642(3.405-3.895) | 4.216(3.426-5.188) | 4.88(3.436-6.931) | 5.65(3.446-9.262) | 6.54(3.455-12.38) | 18.29397 | 84.83848 |
| Male | Timor-Leste | 2.092(2.041-2.145) | 2.233(2.069-2.41) | 2.384(2.096-2.711) | 2.544(2.122-3.051) | 2.716(2.149-3.433) | 62.55043 | 31.64364 |
| Male | Viet Nam | 3.581(3.553-3.609) | 3.859(3.767-3.954) | 4.159(3.993-4.333) | 4.483(4.232-4.749) | 4.832(4.486-5.204) | 144.6836 | 36.89611 |
| Female | Southeast Asia | 3.17(3.147-3.194) | 3.569(3.486-3.653) | 4.017(3.862-4.178) | 4.521(4.277-4.779) | 5.089(4.737-5.467) | 99.08112 | 64.48319 |
| Female | Cambodia | 2.355(2.344-2.366) | 2.737(2.698-2.777) | 3.181(3.105-3.26) | 3.698(3.573-3.827) | 4.298(4.112-4.492) | 95.14457 | 88.12282 |
| Female | Indonesia | 3.883(3.86-3.906) | 4.432(4.351-4.514) | 5.059(4.904-5.218) | 5.774(5.528-6.031) | 6.591(6.23-6.972) | 134.9702 | 74.6374 |
| Female | Lao People's Democratic Republic | 1.06(0.841-1.337) | 0.488(0.239-0.999) | 0.225(0.067-0.754) | 0.104(0.019-0.569) | 0.048(0.005-0.43) | -1.8066 | -96.493 |
| Female | Malaysia | 2.452(2.325-2.585) | 2.53(2.148-2.98) | 2.612(1.98-3.444) | 2.695(1.825-3.981) | 2.782(1.682-4.602) | 112.6806 | 13.74467 |
| Female | Maldives | 3.676(3.446-3.921) | 4.313(3.534-5.263) | 5.06(3.614-7.084) | 5.936(3.694-9.539) | 6.964(3.776-12.847) | 68.27504 | 95.7658 |
| Female | Mauritius | 3.416(3.098-3.767) | 3.94(2.913-5.329) | 4.545(2.728-7.57) | 5.242(2.554-10.76) | 6.047(2.39-15.296) | 29.18277 | 84.68227 |
| Female | Myanmar | 2.745(2.722-2.768) | 3.087(3.008-3.168) | 3.473(3.324-3.628) | 3.906(3.673-4.155) | 4.394(4.058-4.759) | 89.01115 | 64.01324 |
| Female | Philippines | 2.93(2.843-3.019) | 3.214(2.929-3.526) | 3.526(3.014-4.123) | 3.868(3.101-4.823) | 4.243(3.191-5.641) | 35.12271 | 47.59411 |
| Female | Sri Lanka | 2.202(2.137-2.269) | 2.344(2.138-2.571) | 2.496(2.136-2.916) | 2.658(2.134-3.309) | 2.83(2.132-3.755) | 103.04 | 29.92118 |
| Female | Seychelles | 3.93(3.849-4.013) | 4.503(4.222-4.803) | 5.16(4.628-5.754) | 5.913(5.071-6.894) | 6.775(5.557-8.26) | 52.01531 | 77.47418 |
| Female | Thailand | 2.764(2.67-2.862) | 3.179(2.857-3.537) | 3.656(3.053-4.379) | 4.205(3.261-5.423) | 4.836(3.483-6.715) | 45.06428 | 80.00885 |
| Female | Timor-Leste | 2.197(2.117-2.281) | 2.293(2.043-2.574) | 2.393(1.969-2.908) | 2.497(1.897-3.287) | 2.606(1.828-3.715) | 105.9888 | 19.6585 |
| Female | Viet Nam | 3.072(3.048-3.096) | 3.447(3.365-3.531) | 3.869(3.714-4.029) | 4.341(4.099-4.598) | 4.872(4.524-5.246) | 168.9589 | 62.2326 |


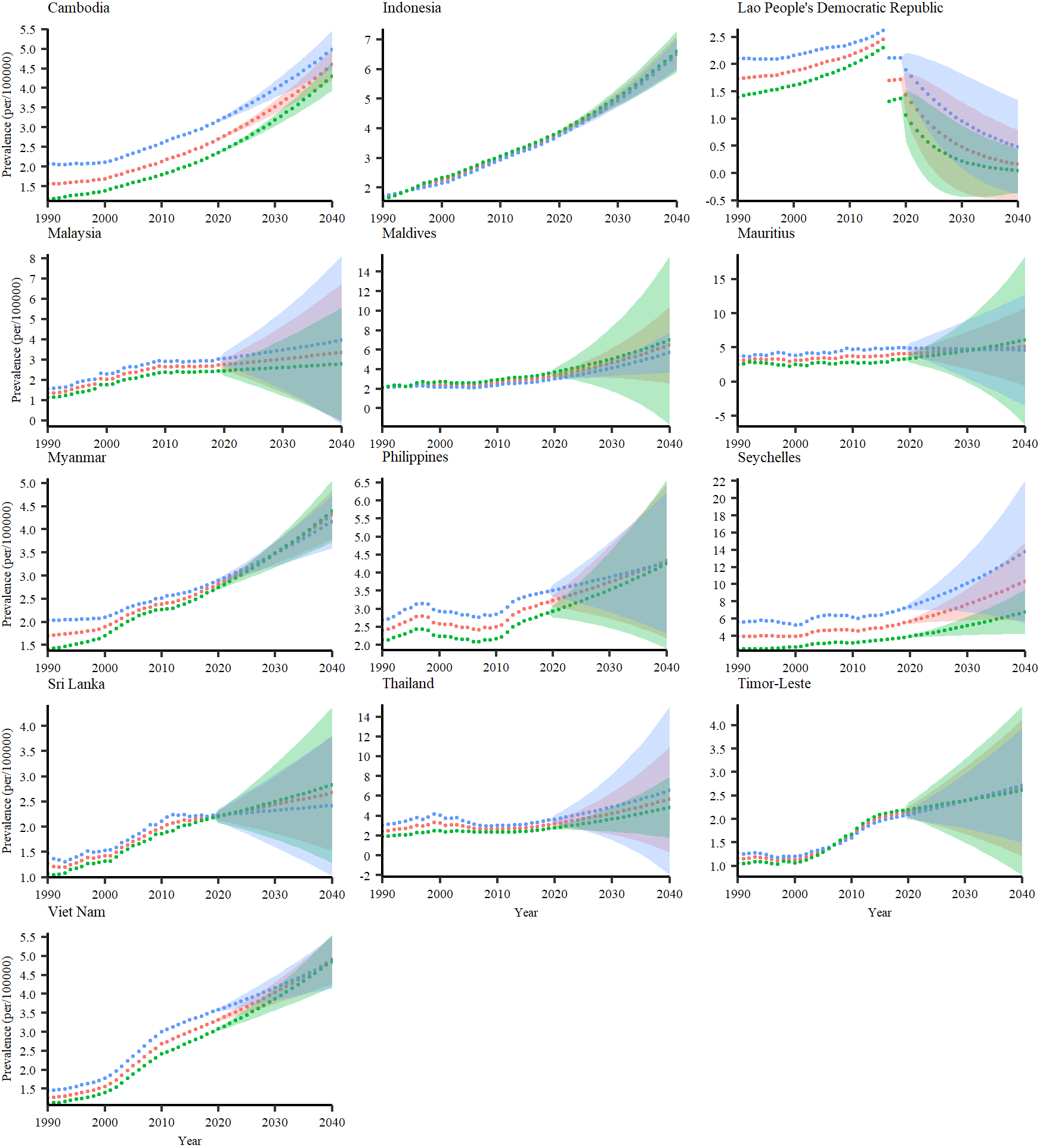


Supplemental Figure 32. Observed and projected age-standardized prevalence rate (ASPR) values from 1990 to 2040 for both sex (Red lines), females (Green lines), and men (Blue lines) in the Southeast Asia. The halo effect observed in each scatter plot accurately represents projections that extend across the temporal span from 2019 to 2040 with 95% confidence intervals.


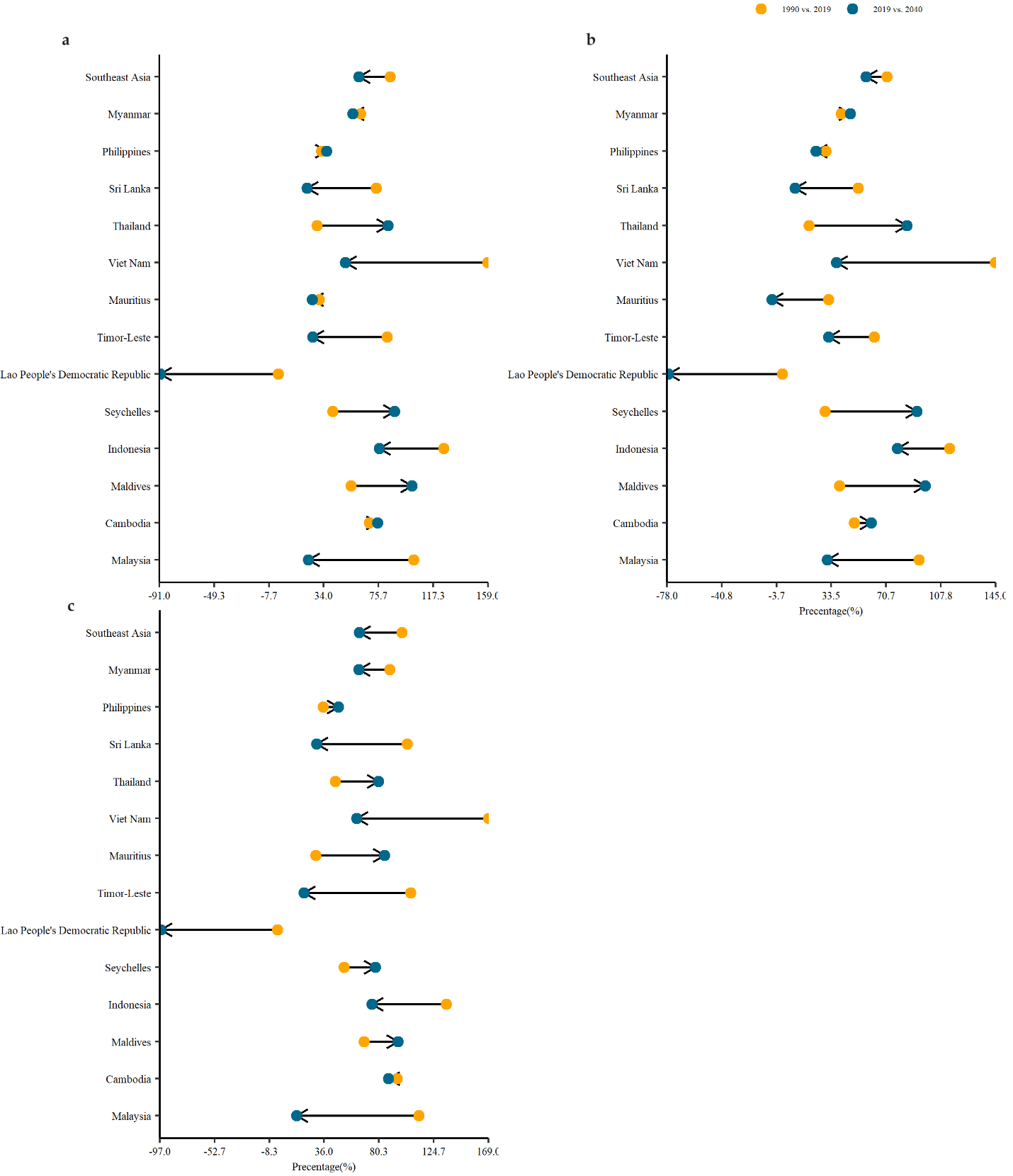


Supplemental Figure 33. The Lollipop plot between the two calculated percentage changes from 1990 to 2019 and 2019 to 2040 for both sexes (a), males (b), and females (c) in the Southeast Asia. Each line represents two time periods and show the change of ASPR increase or decrease during time.

# Southern Latin America

| Supplemental Table 17: Age-standardized prevalence rates (ASPR) from 2020 to 2040, and percentage changes for the time periods 1990 to 2019 and 2019 to 2040, for Southern Latin America. | | | | | | | | |
| --- | --- | --- | --- | --- | --- | --- | --- | --- |
| Group | Country | 2020 | 2025 | 2030 | 2035 | 2040 | 1990 vs. 2019 | 2019 vs. 2040 |
| Both | Argentina | 9.062(8.707-9.433) | 9.7(8.572-10.976) | 10.382(8.424-12.794) | 11.112(8.278-14.917) | 11.893(8.133-17.392) | 31.73303 | 33.7569 |
| Both | Chile | 6.012(5.769-6.265) | 6.245(5.499-7.092) | 6.488(5.233-8.043) | 6.74(4.979-9.124) | 7.002(4.736-10.35) | 19.43029 | 17.31553 |
| Both | Uruguay | 10.937(10.524-11.367) | 11.486(10.197-12.937) | 12.062(9.865-14.748) | 12.667(9.541-16.817) | 13.302(9.227-19.176) | 41.57694 | 22.87842 |
| Male | Argentina | 10.099(9.626-10.596) | 10.973(9.461-12.728) | 11.923(9.28-15.319) | 12.955(9.1-18.443) | 14.076(8.922-22.206) | 26.9958 | 42.69857 |
| Male | Chile | 5.94(5.638-6.258) | 5.952(5.066-6.992) | 5.963(4.543-7.828) | 5.975(4.072-8.768) | 5.987(3.649-9.821) | 18.82611 | 0.440601 |
| Male | Uruguay | 12.675(12.106-13.27) | 13.75(11.932-15.844) | 14.916(11.738-18.954) | 16.181(11.544-22.68) | 17.554(11.353-27.142) | 37.56186 | 41.24994 |
| Female | Argentina | 8.116(7.84-8.402) | 8.522(7.658-9.484) | 8.949(7.47-10.72) | 9.396(7.284-12.12) | 9.866(7.103-13.704) | 37.2162 | 23.22094 |
| Female | Chile | 6.021(5.794-6.257) | 6.451(5.73-7.263) | 6.912(5.658-8.445) | 7.406(5.585-9.821) | 7.935(5.513-11.422) | 19.77686 | 33.9603 |
| Female | Uruguay | 9.396(8.945-9.87) | 9.523(8.181-11.085) | 9.653(7.468-12.477) | 9.784(6.814-14.047) | 9.917(6.218-15.816) | 45.77261 | 5.551972 |


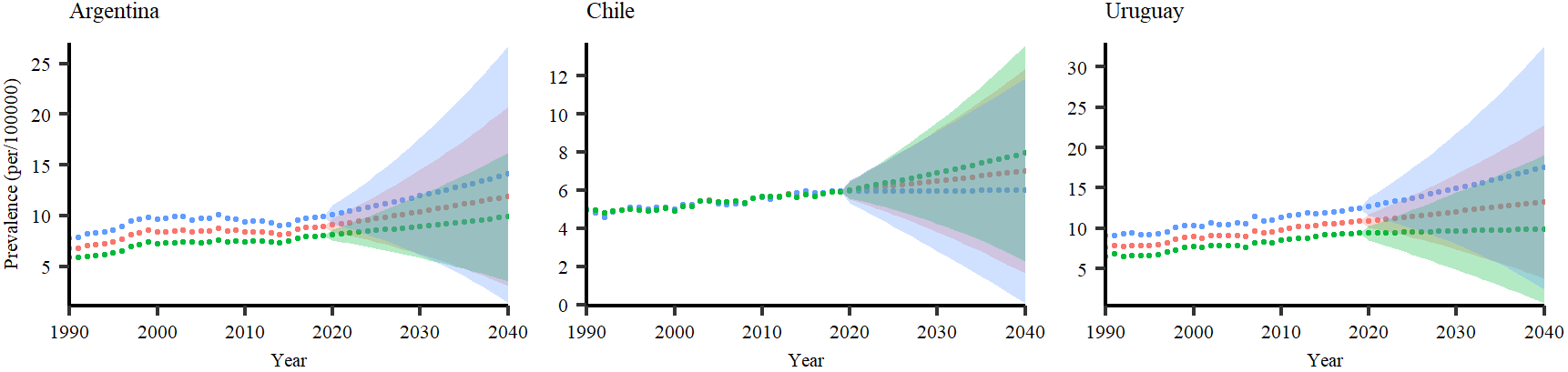


Supplemental Figure 34. Observed and projected age-standardized prevalence rate (ASPR) values from 1990 to 2040 for both sex (Red lines), females (Green lines), and men (Blue lines) in the Southern Latin America. The halo effect observed in each scatter plot accurately represents projections that extend across the temporal span from 2019 to 2040 with 95% confidence intervals.


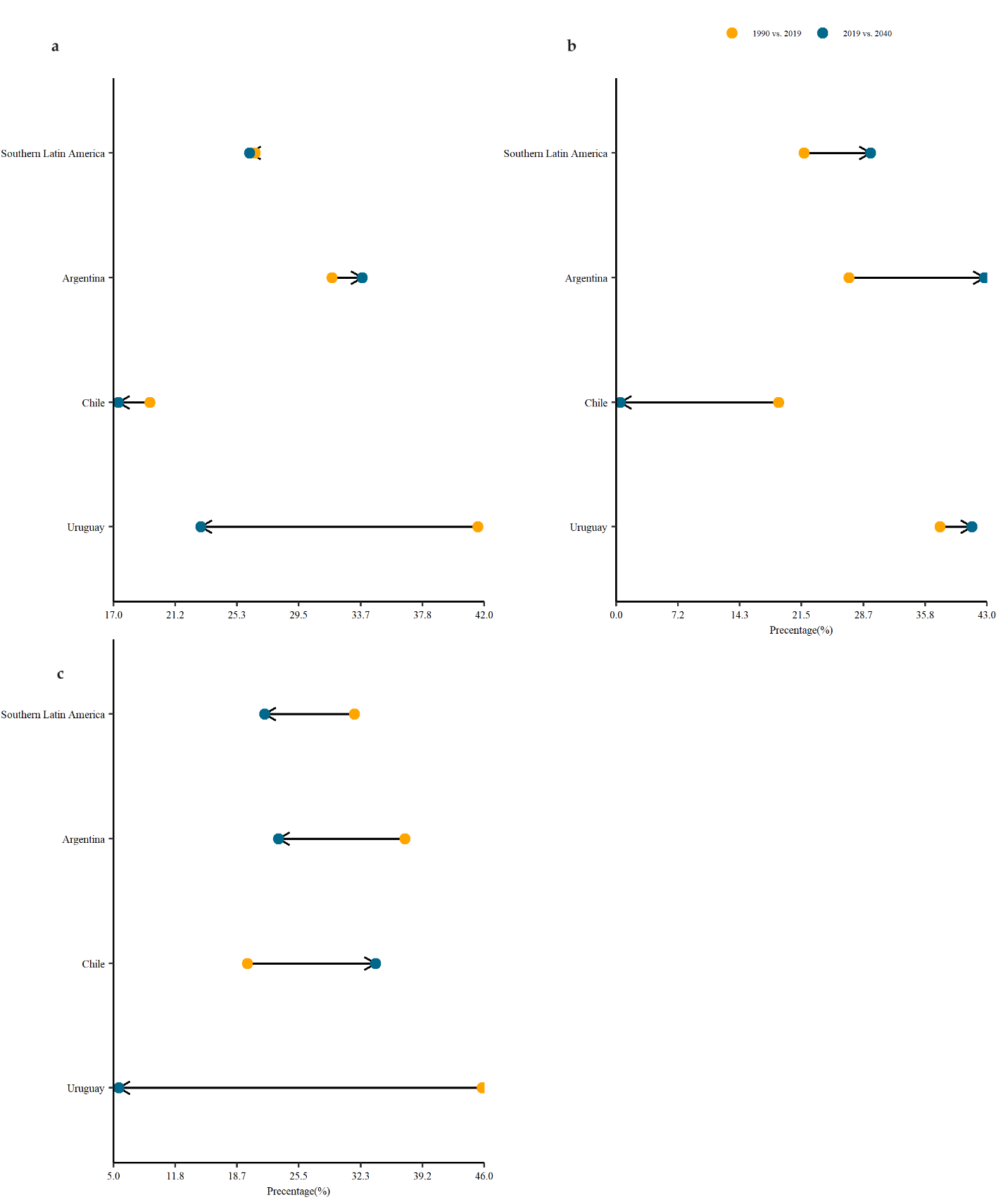


Supplemental Figure 35. The Lollipop plot between the two calculated percentage changes from 1990 to 2019 and 2019 to 2040 for both sexes (a), males (b), and females (c) in the Southern Latin America. Each line represents two time periods and show the change of ASPR increase or decrease during time.

# Southern Sub-Saharan Africa

| Supplemental Table 18: Age-standardized prevalence rates (ASPR) from 2020 to 2040, and percentage changes for the time periods 1990 to 2019 and 2019 to 2040, for Southern Sub-Saharan Africa. | | | | | | | | |
| --- | --- | --- | --- | --- | --- | --- | --- | --- |
| Group | Country | 2020 | 2025 | 2030 | 2035 | 2040 | 1990 vs. 2019 | 2019 vs. 2040 |
| Both | Botswana | 5.714(5.622-5.809) | 6.404(6.089-6.735) | 7.176(6.591-7.814) | 8.042(7.133-9.067) | 9.012(7.719-10.521) | 112.9112 | 61.39076 |
| Both | Lesotho | 4.161(4.106-4.217) | 4.429(4.25-4.615) | 4.713(4.396-5.053) | 5.016(4.547-5.533) | 5.339(4.704-6.059) | 119.8067 | 29.70185 |
| Both | Namibia | 2.829(2.766-2.893) | 3.035(2.831-3.253) | 3.256(2.896-3.661) | 3.493(2.961-4.121) | 3.748(3.028-4.639) | 122.1364 | 34.18431 |
| Both | South Africa | 4.198(3.989-4.419) | 3.604(3.076-4.222) | 3.094(2.368-4.042) | 2.656(1.822-3.871) | 2.28(1.401-3.708) | 29.63974 | -47.6143 |
| Both | Eswatini | 5.66(5.53-5.793) | 5.907(5.498-6.345) | 6.164(5.461-6.957) | 6.433(5.424-7.63) | 6.713(5.386-8.367) | 70.22516 | 19.34264 |
| Both | Zimbabwe | 5.72(5.554-5.892) | 6.08(5.55-6.662) | 6.463(5.539-7.541) | 6.87(5.527-8.538) | 7.302(5.515-9.668) | 62.47136 | 28.91061 |
| Male | Botswana | 6.42(6.317-6.524) | 7.211(6.861-7.578) | 8.1(7.447-8.809) | 9.098(8.083-10.24) | 10.219(8.772-11.905) | 88.77634 | 63.06169 |
| Male | Lesotho | 5.202(5.159-5.246) | 5.55(5.408-5.696) | 5.921(5.667-6.187) | 6.317(5.939-6.72) | 6.74(6.223-7.3) | 93.11313 | 31.14761 |
| Male | Namibia | 3.051(2.981-3.123) | 3.23(3.006-3.471) | 3.42(3.029-3.861) | 3.621(3.051-4.296) | 3.833(3.074-4.78) | 107.4618 | 26.82864 |
| Male | South Africa | 5.119(4.871-5.379) | 4.454(3.821-5.192) | 3.875(2.991-5.022) | 3.372(2.341-4.858) | 2.934(1.831-4.701) | 27.52223 | -44.488 |
| Male | Eswatini | 8.236(7.961-8.52) | 8.548(7.698-9.493) | 8.873(7.433-10.592) | 9.21(7.175-11.82) | 9.559(6.927-13.192) | 79.71914 | 16.60158 |
| Male | Zimbabwe | 4.671(4.535-4.81) | 5.056(4.618-5.535) | 5.473(4.696-6.379) | 5.925(4.775-7.352) | 6.414(4.855-8.474) | 36.53913 | 39.52367 |
| Female | Botswana | 5.083(4.968-5.202) | 5.667(5.279-6.085) | 6.319(5.604-7.125) | 7.045(5.948-8.344) | 7.854(6.313-9.772) | 143.8257 | 57.86175 |
| Female | Lesotho | 3.35(3.236-3.469) | 3.551(3.189-3.953) | 3.763(3.139-4.512) | 3.989(3.089-5.151) | 4.227(3.039-5.88) | 166.9779 | 27.30419 |
| Female | Namibia | 2.651(2.591-2.713) | 2.877(2.68-3.089) | 3.121(2.769-3.519) | 3.387(2.86-4.011) | 3.675(2.954-4.571) | 138.518 | 40.73516 |
| Female | South Africa | 3.463(3.259-3.68) | 2.911(2.414-3.511) | 2.447(1.783-3.359) | 2.057(1.317-3.214) | 1.73(0.972-3.076) | 32.97377 | -52.0739 |
| Female | Eswatini | 3.844(3.776-3.914) | 4.059(3.839-4.292) | 4.286(3.9-4.709) | 4.525(3.962-5.168) | 4.777(4.025-5.671) | 66.68462 | 25.45088 |
| Female | Zimbabwe | 6.387(6.144-6.639) | 6.679(5.926-7.527) | 6.984(5.706-8.548) | 7.303(5.493-9.71) | 7.637(5.287-11.03) | 79.77092 | 20.20305 |


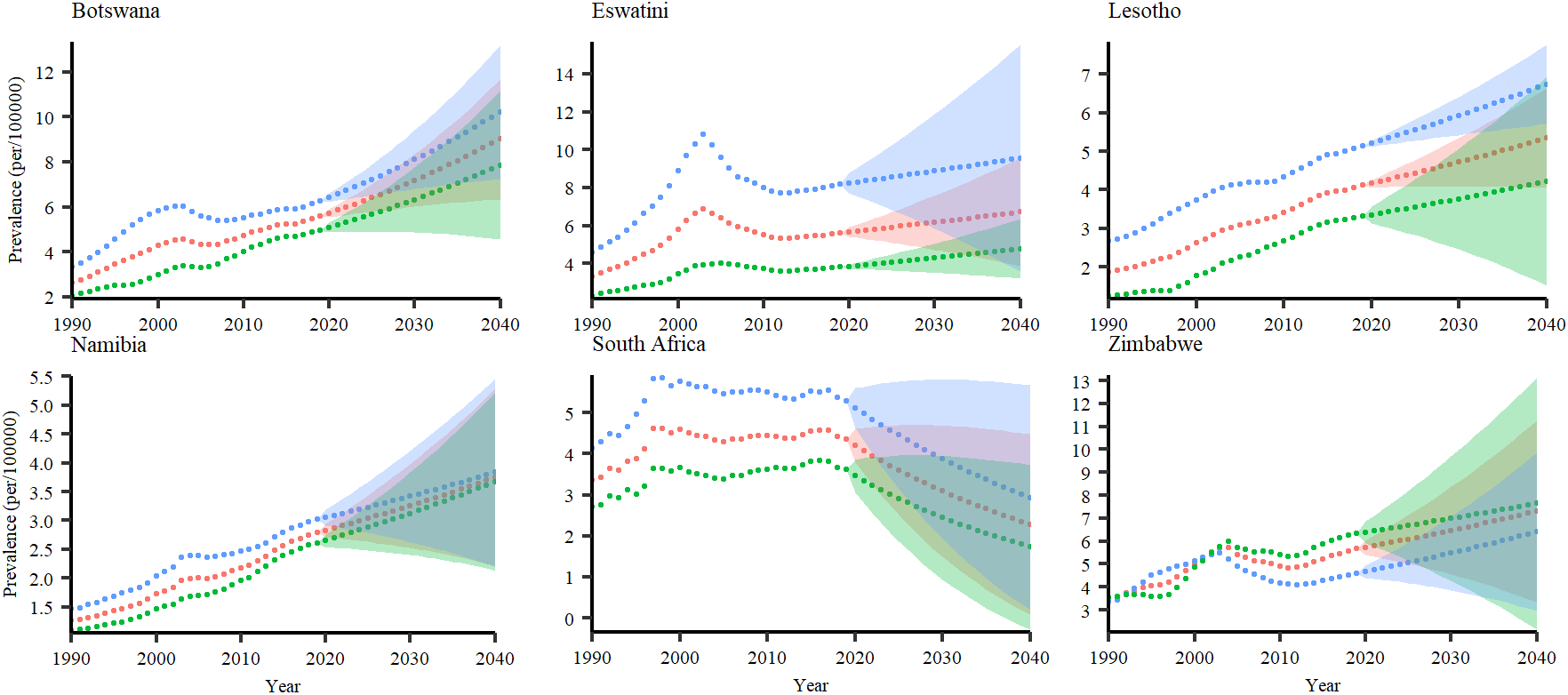


Supplemental Figure 36. Observed and projected age-standardized prevalence rate (ASPR) values from 1990 to 2040 for both sex (Red lines), females (Green lines), and men (Blue lines) in the Southern Sub-Saharan Africa. The halo effect observed in each scatter plot accurately represents projections that extend across the temporal span from 2019 to 2040 with 95% confidence intervals.


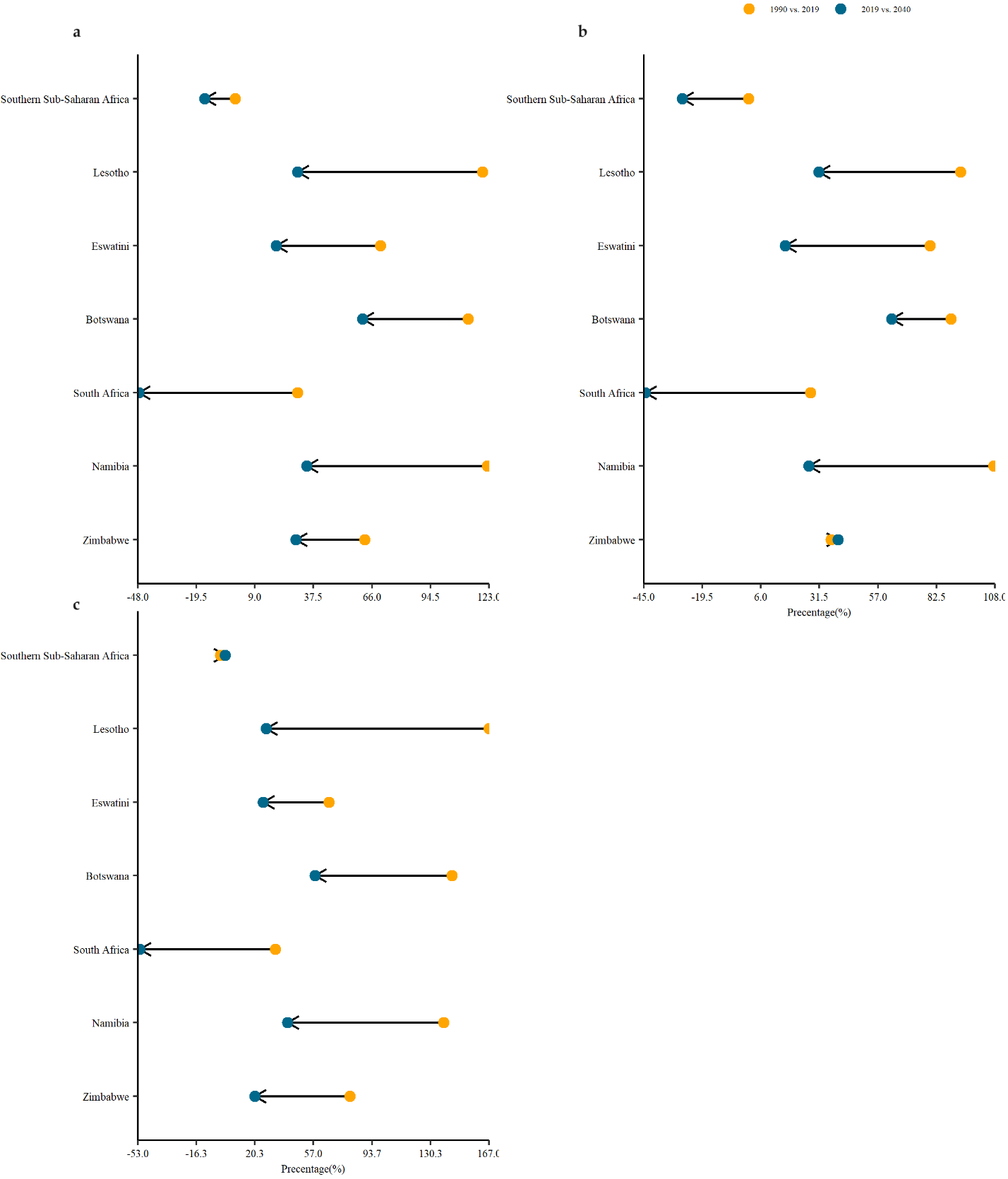


Supplemental Figure 37. The Lollipop plot between the two calculated percentage changes from 1990 to 2019 and 2019 to 2040 for both sexes (a), males (b), and females (c) in the Southern Sub-Saharan Africa. Each line represents two time periods and show the change of ASPR increase or decrease during time.
