# Supplementary material for: Global Trend in Pancreatic Cancer Prevalence Rates Through 2040: An Illness‐Death Modeling Study
Source: Cancer Med. 2024 Oct 23;13(20):e70318. doi: 10.1002/cam4.70318 (PMC11497012; doi:10.1002/cam4.70318)
Supplement: Supplementary file 5 — Data S5. [file CAM4-13-e70318-s005.docx]

#

# Tropical Latin America

| Supplemental Table 19: Age-standardized prevalence rates (ASPR) from 2020 to 2040, and percentage changes for the time periods 1990 to 2019 and 2019 to 2040, for Tropical Latin America. | | | | | | | | |
| --- | --- | --- | --- | --- | --- | --- | --- | --- |
| Group | Country | 2020 | 2025 | 2030 | 2035 | 2040 | 1990 vs. 2019 | 2019 vs. 2040 |
| Both | Brazil | 4.693(4.595-4.793) | 4.706(4.408-5.024) | 4.719(4.225-5.27) | 4.732(4.049-5.529) | 4.745(3.881-5.801) | 22.42667 | 0.830283 |
| Both | Paraguay | 4.536(4.362-4.717) | 4.747(4.206-5.357) | 4.967(4.049-6.093) | 5.197(3.897-6.932) | 5.439(3.75-7.887) | 181.0147 | 20.84267 |
| Male | Brazil | 5.147(5.025-5.273) | 5.115(4.749-5.509) | 5.083(4.484-5.762) | 5.051(4.233-6.027) | 5.019(3.996-6.304) | 21.95779 | -3.0883 |
| Male | Paraguay | 4.928(4.737-5.126) | 5.051(4.471-5.706) | 5.177(4.213-6.362) | 5.306(3.969-7.094) | 5.439(3.739-7.912) | 193.2949 | 10.3992 |
| Female | Brazil | 4.274(4.191-4.359) | 4.318(4.063-4.588) | 4.362(3.937-4.833) | 4.407(3.813-5.092) | 4.452(3.694-5.365) | 23.553 | 4.176084 |
| Female | Paraguay | 4.143(3.949-4.346) | 4.443(3.834-5.15) | 4.765(3.714-6.115) | 5.111(3.597-7.262) | 5.481(3.483-8.626) | 167.6171 | 34.51847 |


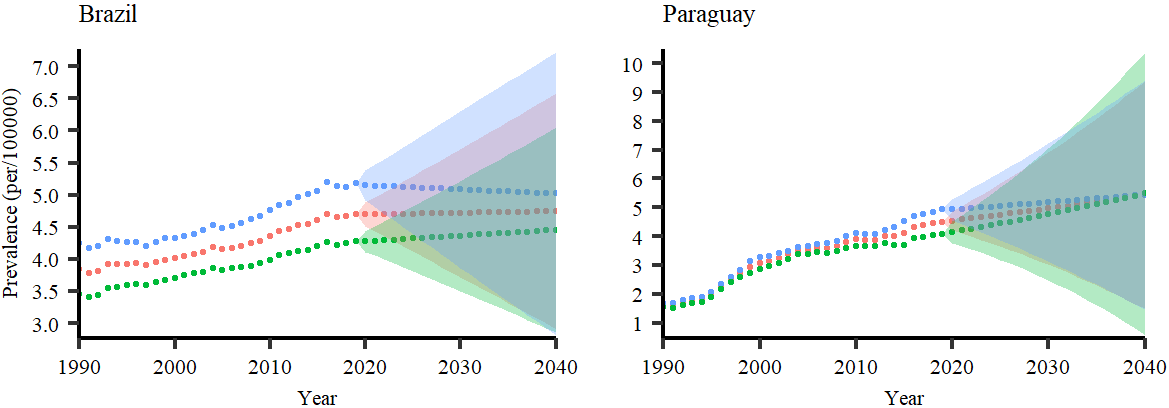


Supplemental Figure 38. Observed and projected age-standardized prevalence rate (ASPR) values from 1990 to 2040 for both sex (Red lines), females (Green lines), and men (Blue lines) in the Tropical Latin America. The halo effect observed in each scatter plot accurately represents projections that extend across the temporal span from 2019 to 2040 with 95% confidence intervals.


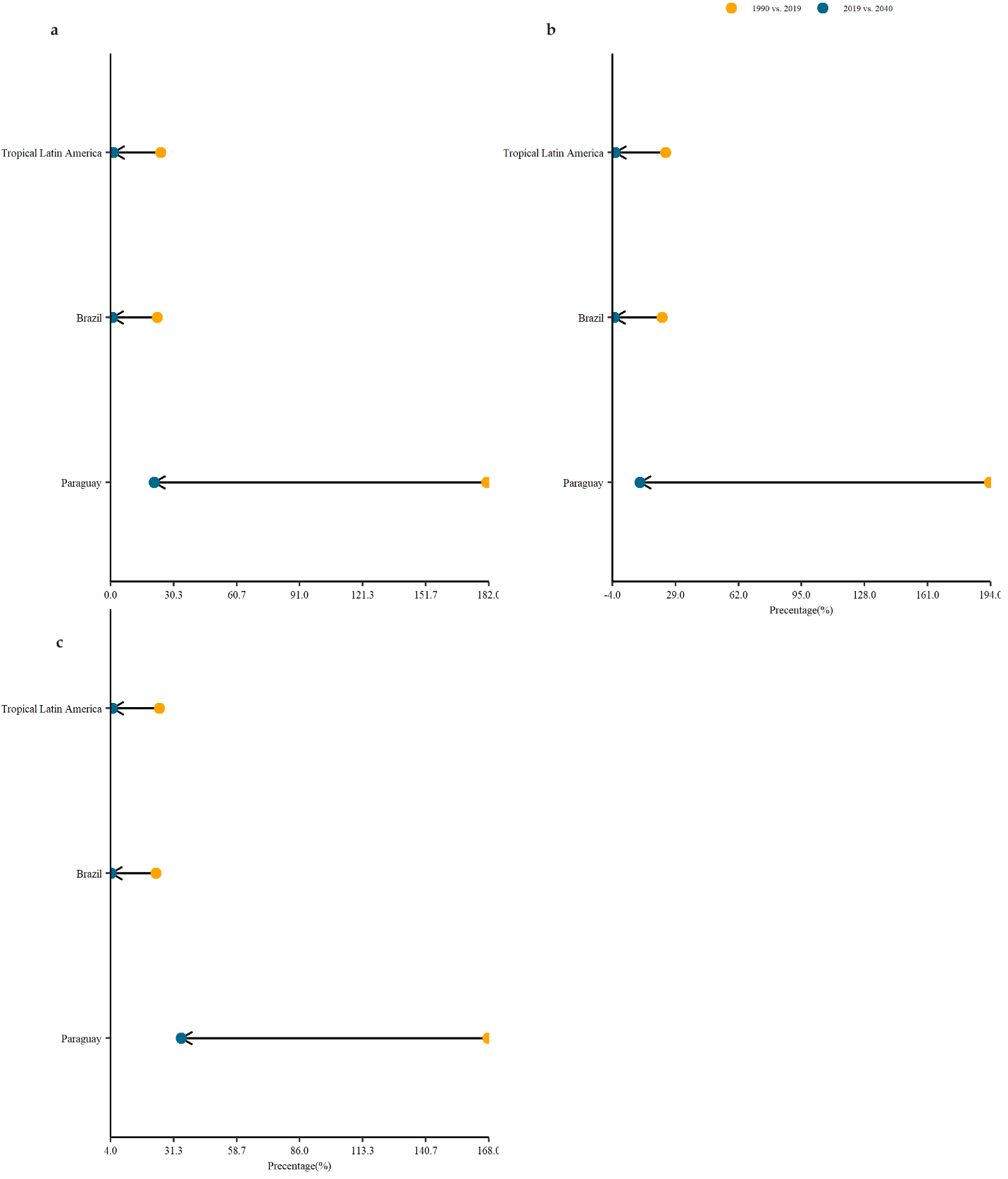


Supplemental Figure 39. The Lollipop plot between the two calculated percentage changes from 1990 to 2019 and 2019 to 2040 for both sexes (a), males (b), and females (c) in the Tropical Latin America. Each line represents two time periods and show the change of ASPR increase or decrease during time.

# Western Europe

| Supplemental Table 20: Age-standardized prevalence rates (ASPR) from 2020 to 2040, and percentage changes for the time periods 1990 to 2019 and 2019 to 2040, for Western Europe. | | | | | | | | |
| --- | --- | --- | --- | --- | --- | --- | --- | --- |
| Group | Country | 2020 | 2025 | 2030 | 2035 | 2040 | 1990 vs. 2019 | 2019 vs. 2040 |
| Both | Andorra | 8.932(8.794-9.073) | 9.042(8.616-9.489) | 9.153(8.436-9.931) | 9.265(8.259-10.394) | 9.379(8.086-10.879) | 20.74971 | 5.148397 |
| Both | Austria | 9.636(9.383-9.897) | 8.845(8.146-9.604) | 8.119(7.065-9.33) | 7.452(6.126-9.066) | 6.84(5.312-8.809) | 11.75616 | -30.7314 |
| Both | Belgium | 7.779(7.579-7.985) | 7.531(6.947-8.164) | 7.291(6.362-8.357) | 7.059(5.825-8.555) | 6.834(5.332-8.758) | 13.14894 | -12.9313 |
| Both | Cyprus | 6.498(6.212-6.797) | 5.982(5.207-6.874) | 5.508(4.356-6.964) | 5.071(3.643-7.058) | 4.668(3.046-7.153) | 120.1587 | -29.9192 |
| Both | Denmark | 8.601(8.145-9.084) | 8.186(6.917-9.687) | 7.79(5.861-10.355) | 7.414(4.964-11.073) | 7.056(4.205-11.841) | 49.5276 | -18.6272 |
| Both | Finland | 11.585(11.23-11.951) | 11.008(10-12.117) | 10.46(8.893-12.303) | 9.939(7.907-12.493) | 9.444(7.03-12.687) | 17.51313 | -19.3243 |
| Both | France | 8.256(7.994-8.527) | 8.009(7.249-8.848) | 7.768(6.564-9.193) | 7.535(5.943-9.554) | 7.309(5.38-9.929) | 37.0497 | -12.5038 |
| Both | Germany | 12.727(12.418-13.043) | 12.313(11.415-13.281) | 11.912(10.481-13.538) | 11.524(9.622-13.802) | 11.149(8.834-14.071) | 63.16459 | -13.2787 |
| Both | Greece | 8.556(8.247-8.876) | 9.029(8.061-10.112) | 9.528(7.867-11.539) | 10.055(7.676-13.17) | 10.611(7.489-15.033) | 17.13397 | 25.34229 |
| Both | Iceland | 7.298(7.11-7.492) | 7.033(6.487-7.626) | 6.778(5.912-7.771) | 6.531(5.387-7.919) | 6.294(4.908-8.071) | 4.046054 | -15.1194 |
| Both | Ireland | 7.885(7.581-8.2) | 8.145(7.216-9.195) | 8.415(6.856-10.327) | 8.693(6.514-11.601) | 8.98(6.188-13.033) | 6.569974 | 14.69661 |
| Both | Israel | 8.651(8.366-8.946) | 8.916(8.039-9.889) | 9.19(7.715-10.946) | 9.471(7.402-12.119) | 9.762(7.101-13.419) | 24.76373 | 13.61158 |
| Both | Italy | 9.23(8.925-9.545) | 8.97(8.086-9.95) | 8.717(7.316-10.387) | 8.472(6.618-10.845) | 8.234(5.986-11.325) | 26.71204 | -11.6428 |
| Both | Luxembourg | 7.49(7.326-7.657) | 7.639(7.135-8.177) | 7.79(6.943-8.741) | 7.945(6.755-9.345) | 8.103(6.571-9.991) | -2.03106 | 8.689843 |
| Both | Malta | 7.457(7.308-7.609) | 7.438(6.988-7.916) | 7.418(6.677-8.242) | 7.399(6.379-8.583) | 7.38(6.094-8.938) | 19.06098 | -1.11409 |
| Both | Netherlands | 9.612(9.219-10.022) | 9.369(8.235-10.659) | 9.132(7.344-11.356) | 8.901(6.547-12.102) | 8.676(5.836-12.897) | 38.47743 | -10.168 |
| Both | Norway | 8.689(8.45-8.934) | 8.076(7.411-8.801) | 7.507(6.492-8.681) | 6.978(5.687-8.563) | 6.486(4.981-8.448) | 15.01899 | -26.6469 |
| Both | Portugal | 5.334(5.143-5.531) | 5.302(4.739-5.932) | 5.27(4.359-6.37) | 5.238(4.01-6.843) | 5.207(3.688-7.352) | 4.190331 | -2.33293 |
| Both | Spain | 7.426(7.241-7.615) | 7.1(6.57-7.673) | 6.789(5.954-7.74) | 6.491(5.396-7.808) | 6.206(4.889-7.878) | 27.27495 | -17.5091 |
| Both | Sweden | 6.242(6.054-6.436) | 6.308(5.74-6.933) | 6.376(5.436-7.478) | 6.443(5.146-8.068) | 6.512(4.872-8.704) | -10.5516 | 5.083542 |
| Both | Switzerland | 9.04(8.646-9.452) | 8.667(7.552-9.946) | 8.309(6.584-10.486) | 7.967(5.739-11.058) | 7.638(5.002-11.662) | 59.71857 | -16.1201 |
| Both | United Kingdom | 7.607(7.43-7.789) | 7.601(7.067-8.174) | 7.594(6.715-8.588) | 7.588(6.38-9.024) | 7.581(6.061-9.482) | 12.13795 | -0.38189 |
| Male | Andorra | 11.228(11.06-11.398) | 11.468(10.949-12.013) | 11.714(10.832-12.669) | 11.965(10.714-13.362) | 12.222(10.598-14.094) | 17.33868 | 9.356487 |
| Male | Austria | 10.405(10.05-10.772) | 9.419(8.462-10.484) | 8.526(7.114-10.218) | 7.718(5.98-9.961) | 6.987(5.026-9.711) | 0.331248 | -34.6706 |
| Male | Belgium | 8.604(8.394-8.819) | 8.469(7.848-9.139) | 8.336(7.33-9.48) | 8.205(6.845-9.836) | 8.076(6.391-10.205) | 3.725673 | -6.49706 |
| Male | Cyprus | 7.615(7.315-7.928) | 7.091(6.262-8.03) | 6.603(5.352-8.146) | 6.149(4.574-8.267) | 5.726(3.908-8.389) | 120.6289 | -26.5787 |
| Male | Denmark | 9.454(8.859-10.089) | 9.099(7.444-11.122) | 8.757(6.237-12.294) | 8.428(5.225-13.594) | 8.111(4.376-15.034) | 56.90072 | -14.6322 |
| Male | Finland | 12.379(12.031-12.736) | 11.598(10.621-12.664) | 10.866(9.365-12.607) | 10.18(8.256-12.553) | 9.538(7.279-12.499) | 12.28468 | -23.9264 |
| Male | France | 9.685(9.354-10.028) | 9.241(8.3-10.288) | 8.817(7.353-10.571) | 8.412(6.514-10.864) | 8.026(5.77-11.166) | 21.02407 | -18.373 |
| Male | Germany | 12.586(12.261-12.92) | 12.07(11.133-13.086) | 11.575(10.097-13.269) | 11.1(9.156-13.457) | 10.645(8.303-13.648) | 42.16787 | -16.4879 |
| Male | Greece | 9.789(9.34-10.259) | 10.11(8.746-11.687) | 10.443(8.174-13.341) | 10.786(7.638-15.232) | 11.141(7.136-17.393) | 11.41444 | 14.30471 |
| Male | Iceland | 9.079(8.718-9.455) | 9.055(7.989-10.265) | 9.032(7.308-11.163) | 9.009(6.684-12.142) | 8.985(6.113-13.208) | 16.59843 | -1.42036 |
| Male | Ireland | 8.418(7.993-8.866) | 8.559(7.292-10.045) | 8.702(6.639-11.406) | 8.847(6.042-12.955) | 8.995(5.499-14.715) | -2.52015 | 7.225386 |
| Male | Israel | 10.028(9.543-10.537) | 10.211(8.763-11.899) | 10.399(8.031-13.465) | 10.589(7.357-15.241) | 10.783(6.739-17.254) | 30.51952 | 8.03873 |
| Male | Italy | 9.209(8.895-9.535) | 8.821(7.924-9.82) | 8.45(7.049-10.129) | 8.094(6.27-10.449) | 7.753(5.576-10.781) | 12.24136 | -16.8265 |
| Male | Luxembourg | 8.414(8.166-8.668) | 8.726(7.959-9.568) | 9.051(7.747-10.575) | 9.387(7.539-11.689) | 9.736(7.336-12.921) | -10.4769 | 16.83346 |
| Male | Malta | 9.031(8.712-9.361) | 9.08(8.126-10.146) | 9.129(7.568-11.012) | 9.179(7.047-11.956) | 9.229(6.561-12.981) | 13.51354 | 2.350919 |
| Male | Netherlands | 9.965(9.513-10.439) | 9.685(8.391-11.178) | 9.412(7.387-11.993) | 9.148(6.502-12.871) | 8.89(5.722-13.814) | 21.63146 | -11.323 |
| Male | Norway | 9.223(8.834-9.63) | 8.515(7.453-9.728) | 7.861(6.277-9.845) | 7.258(5.285-9.967) | 6.7(4.449-10.09) | 5.439178 | -29.109 |
| Male | Portugal | 6.833(6.51-7.173) | 6.742(5.803-7.832) | 6.651(5.163-8.568) | 6.562(4.592-9.376) | 6.474(4.084-10.261) | 6.808311 | -5.15555 |
| Male | Spain | 8.5(8.272-8.735) | 7.885(7.249-8.578) | 7.315(6.345-8.434) | 6.786(5.553-8.293) | 6.295(4.859-8.155) | 21.05554 | -27.3169 |
| Male | Sweden | 6.599(6.371-6.834) | 6.566(5.892-7.316) | 6.533(5.441-7.844) | 6.5(5.023-8.412) | 6.468(4.637-9.022) | -10.4133 | -1.96607 |
| Male | Switzerland | 9.371(8.945-9.818) | 8.857(7.671-10.226) | 8.371(6.565-10.673) | 7.911(5.617-11.142) | 7.477(4.806-11.632) | 60.81837 | -21.1933 |
| Male | United Kingdom | 8.329(8.091-8.574) | 8.325(7.612-9.106) | 8.322(7.153-9.682) | 8.319(6.72-10.297) | 8.315(6.313-10.952) | 6.599822 | -0.21569 |
| Female | Andorra | 6.557(6.444-6.672) | 6.522(6.181-6.882) | 6.488(5.924-7.104) | 6.453(5.678-7.334) | 6.418(5.441-7.572) | 28.62058 | -2.49281 |
| Female | Austria | 8.863(8.507-9.234) | 8.23(7.252-9.339) | 7.642(6.172-9.462) | 7.096(5.251-9.59) | 6.589(4.468-9.719) | 23.54948 | -27.2675 |
| Female | Belgium | 6.979(6.694-7.275) | 6.642(5.842-7.552) | 6.322(5.089-7.853) | 6.017(4.432-8.169) | 5.727(3.859-8.498) | 23.01309 | -19.0737 |
| Female | Cyprus | 5.407(5.104-5.729) | 4.937(4.131-5.9) | 4.507(3.336-6.091) | 4.115(2.692-6.29) | 3.757(2.173-6.497) | 115.3901 | -32.296 |
| Female | Denmark | 7.766(7.377-8.176) | 7.305(6.234-8.561) | 6.872(5.257-8.983) | 6.464(4.431-9.43) | 6.081(3.735-9.899) | 41.49889 | -22.5819 |
| Female | Finland | 10.78(10.343-11.237) | 10.383(9.135-11.8) | 10(8.055-12.414) | 9.631(7.101-13.063) | 9.276(6.259-13.747) | 21.82345 | -14.6446 |
| Female | France | 6.942(6.725-7.167) | 6.847(6.207-7.554) | 6.753(5.721-7.972) | 6.661(5.272-8.415) | 6.57(4.858-8.884) | 59.28468 | -6.14581 |
| Female | Germany | 12.805(12.494-13.124) | 12.481(11.569-13.465) | 12.165(10.701-13.829) | 11.857(9.897-14.206) | 11.557(9.153-14.593) | 85.01012 | -10.4581 |
| Female | Greece | 7.404(7.135-7.684) | 7.995(7.13-8.966) | 8.634(7.114-10.478) | 9.323(7.096-12.248) | 10.067(7.078-14.318) | 24.45811 | 38.36306 |
| Female | Iceland | 5.584(5.419-5.755) | 5.141(4.685-5.641) | 4.733(4.046-5.537) | 4.357(3.493-5.435) | 4.011(3.015-5.336) | -11.0757 | -30.4205 |
| Female | Ireland | 7.356(6.981-7.751) | 7.713(6.563-9.065) | 8.089(6.157-10.627) | 8.482(5.774-12.461) | 8.895(5.415-14.613) | 17.01625 | 22.17514 |
| Female | Israel | 7.388(7.114-7.671) | 7.7(6.854-8.65) | 8.026(6.593-9.77) | 8.365(6.341-11.037) | 8.719(6.097-12.469) | 17.52702 | 19.03414 |
| Female | Italy | 9.203(8.878-9.539) | 9.053(8.103-10.114) | 8.905(7.384-10.74) | 8.76(6.727-11.406) | 8.617(6.128-12.115) | 42.34337 | -7.07019 |
| Female | Luxembourg | 6.555(6.371-6.744) | 6.553(6.003-7.154) | 6.552(5.65-7.598) | 6.55(5.316-8.071) | 6.549(5.002-8.574) | 5.953016 | -0.17864 |
| Female | Malta | 6.002(5.849-6.159) | 5.929(5.475-6.421) | 5.857(5.119-6.702) | 5.786(4.786-6.997) | 5.716(4.474-7.304) | 22.26002 | -5.08363 |
| Female | Netherlands | 9.221(8.82-9.64) | 9.006(7.85-10.331) | 8.795(6.974-11.092) | 8.59(6.195-11.912) | 8.39(5.501-12.794) | 55.73615 | -9.35336 |
| Female | Norway | 8.12(7.859-8.39) | 7.596(6.867-8.403) | 7.106(5.993-8.427) | 6.648(5.228-8.454) | 6.219(4.561-8.48) | 24.87845 | -24.2363 |
| Female | Portugal | 4.044(3.89-4.205) | 4.058(3.599-4.576) | 4.072(3.324-4.988) | 4.085(3.069-5.438) | 4.099(2.834-5.929) | 0.180371 | 1.268052 |
| Female | Spain | 6.382(6.2-6.57) | 6.3(5.761-6.89) | 6.219(5.346-7.235) | 6.139(4.96-7.597) | 6.06(4.602-7.979) | 34.6868 | -5.74139 |
| Female | Sweden | 5.874(5.644-6.113) | 6.025(5.326-6.816) | 6.179(5.017-7.611) | 6.338(4.725-8.502) | 6.501(4.45-9.498) | -11.0706 | 12.24653 |
| Female | Switzerland | 8.664(8.147-9.214) | 8.435(6.975-10.199) | 8.211(5.956-11.319) | 7.993(5.084-12.567) | 7.781(4.339-13.953) | 59.94599 | -10.3691 |
| Female | United Kingdom | 6.913(6.756-7.073) | 6.907(6.435-7.413) | 6.901(6.124-7.777) | 6.895(5.827-8.16) | 6.889(5.543-8.562) | 16.84848 | -0.35289 |


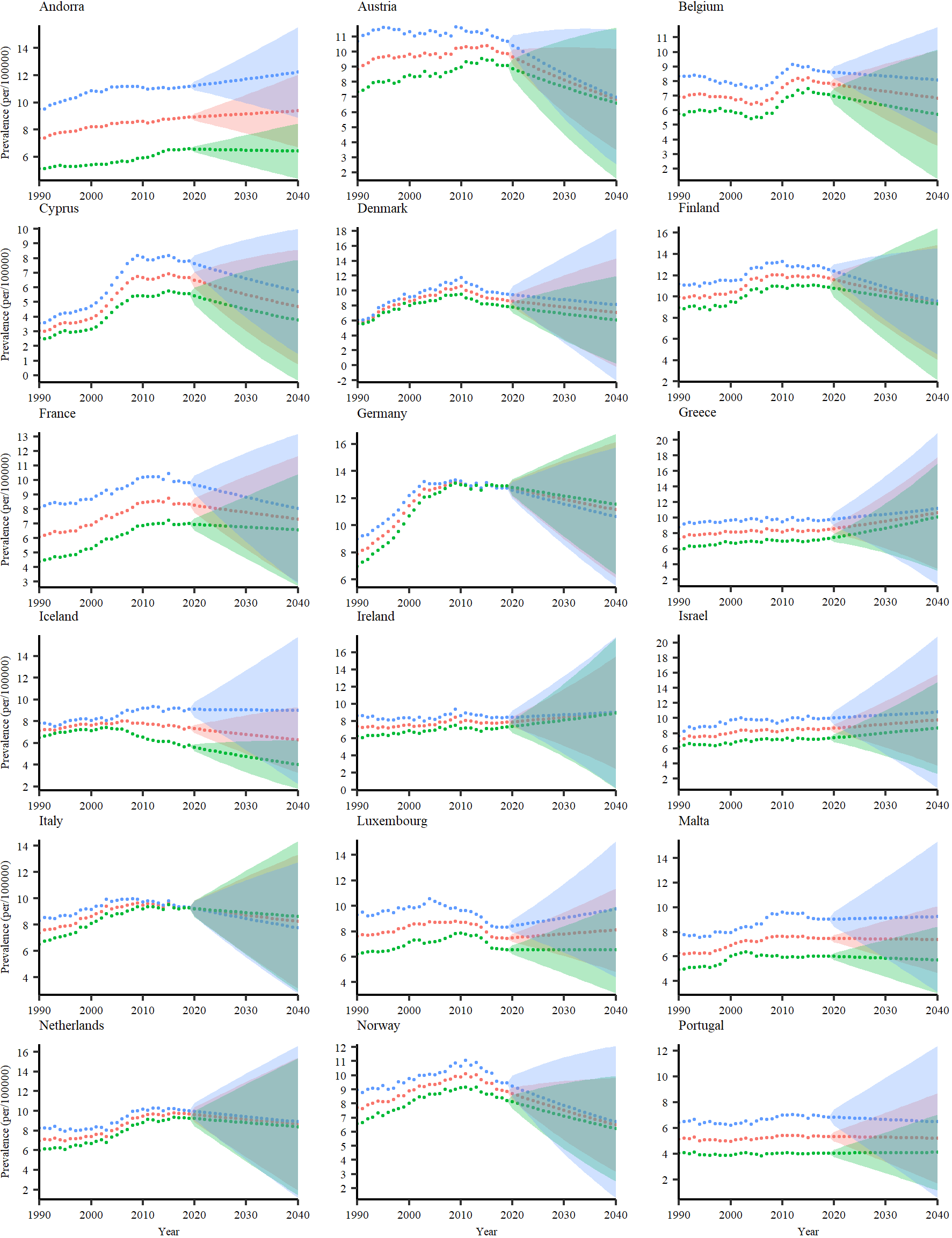


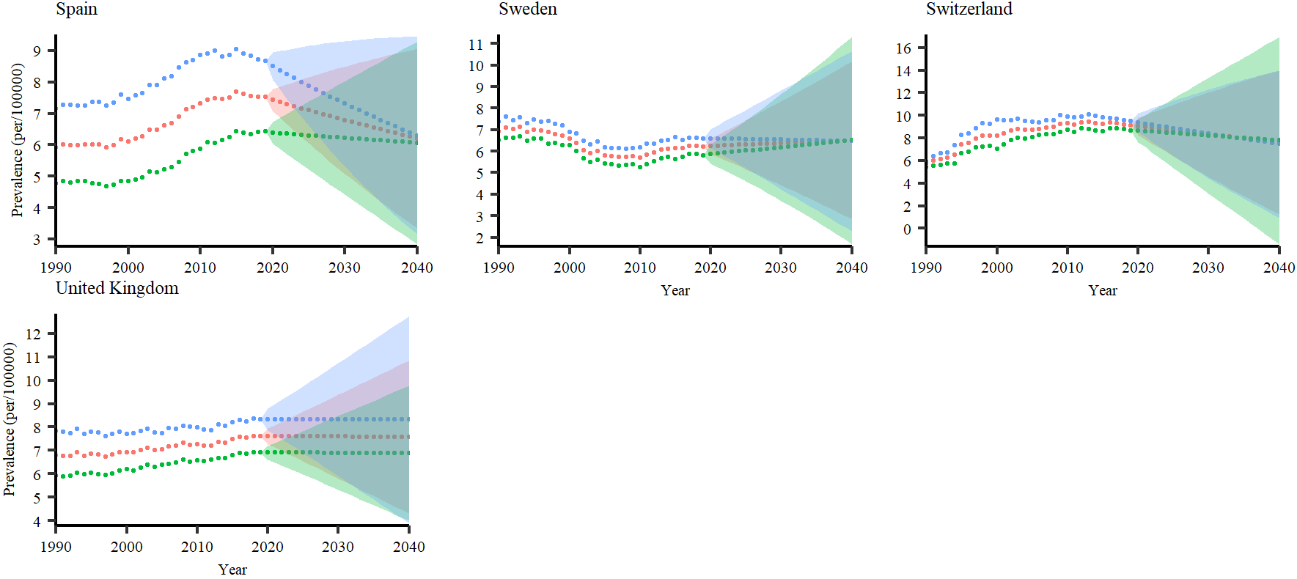


Supplemental Figure 40. Observed and projected age-standardized prevalence rate (ASPR) values from 1990 to 2040 for both sex (Red lines), females (Green lines), and men (Blue lines) in the Western Europe. The halo effect observed in each scatter plot accurately represents projections that extend across the temporal span from 2019 to 2040 with 95% confidence intervals.


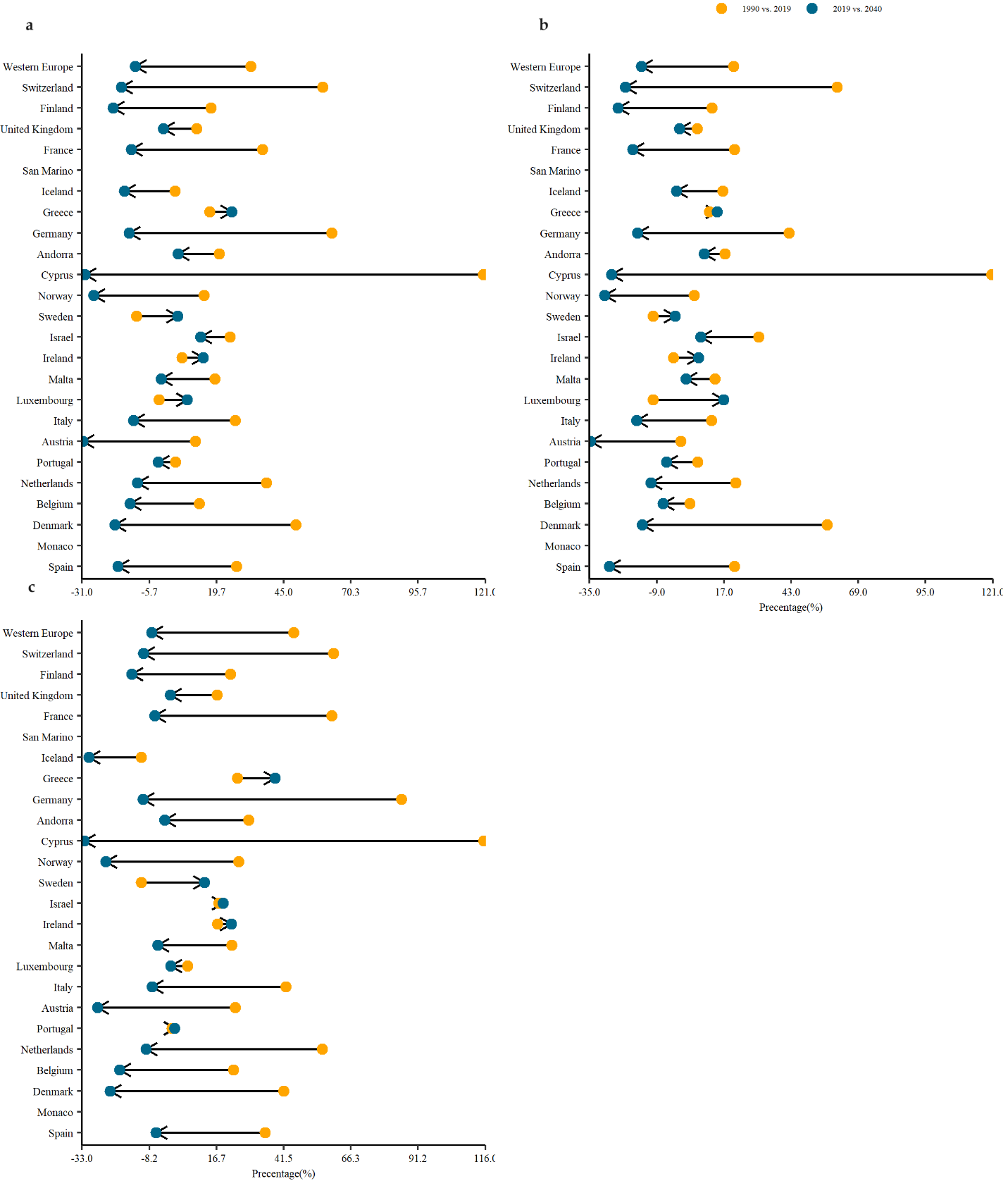


Supplemental Figure 41. The Lollipop plot between the two calculated percentage changes from 1990 to 2019 and 2019 to 2040 for both sexes (a), males (b), and females (c) in the Western Europe. Each line represents two time periods and show the change of ASPR increase or decrease during time.

# Western Sub-Saharan Africa

| Supplemental Table 21: Age-standardized prevalence rates (ASPR) from 2020 to 2040, and percentage changes for the time periods 1990 to 2019 and 2019 to 2040, for Western Sub-Saharan Africa. | | | | | | | | |
| --- | --- | --- | --- | --- | --- | --- | --- | --- |
| Group | Country | 2020 | 2025 | 2030 | 2035 | 2040 | 1990 vs. 2019 | 2019 vs. 2040 |
| Both | Benin | 3.423(3.375-3.472) | 3.729(3.569-3.896) | 4.062(3.772-4.374) | 4.425(3.987-4.911) | 4.82(4.213-5.514) | 113.335 | 43.44514 |
| Both | Burkina Faso | 2.545(2.501-2.59) | 2.816(2.668-2.972) | 3.115(2.844-3.413) | 3.447(3.031-3.92) | 3.814(3.231-4.502) | 99.39409 | 53.79492 |
| Both | Cameroon | 5.611(5.552-5.67) | 6.21(6.012-6.414) | 6.873(6.507-7.259) | 7.607(7.042-8.216) | 8.419(7.621-9.299) | 106.3008 | 53.66023 |
| Both | Cabo Verde | 8.132(7.375-8.966) | 11.171(8.817-14.152) | 15.346(10.5-22.428) | 21.082(12.495-35.57) | 28.961(14.864-56.428) | 741.1309 | 284.1775 |
| Both | Chad | 1.969(1.943-1.996) | 2.114(2.028-2.205) | 2.27(2.115-2.437) | 2.438(2.207-2.693) | 2.618(2.302-2.977) | 101.9557 | 35.24471 |
| Both | Gambia | 2.459(2.29-2.64) | 2.75(2.209-3.423) | 3.075(2.124-4.452) | 3.439(2.042-5.792) | 3.845(1.962-7.537) | 119.6498 | 60.37694 |
| Both | Ghana | 6.983(6.739-7.236) | 8.192(7.34-9.143) | 9.611(7.983-11.571) | 11.275(8.68-14.645) | 13.227(9.438-18.538) | 110.0109 | 96.83909 |
| Both | Guinea | 1.34(1.327-1.354) | 1.446(1.401-1.493) | 1.561(1.478-1.647) | 1.684(1.56-1.817) | 1.817(1.646-2.005) | 61.6369 | 38.07773 |
| Both | Guinea-Bissau | 3.368(3.353-3.382) | 3.57(3.522-3.618) | 3.784(3.699-3.871) | 4.012(3.885-4.142) | 4.252(4.08-4.432) | 74.46946 | 27.69217 |
| Both | Liberia | 3.51(3.449-3.573) | 3.904(3.697-4.121) | 4.341(3.961-4.758) | 4.827(4.242-5.493) | 5.368(4.544-6.343) | 89.8208 | 56.50295 |
| Both | Mali | 2.446(2.421-2.472) | 2.594(2.512-2.68) | 2.752(2.605-2.906) | 2.918(2.702-3.152) | 3.095(2.803-3.418) | 55.98743 | 28.47906 |
| Both | Mauritania | 4.509(4.379-4.643) | 5.062(4.624-5.542) | 5.683(4.878-6.622) | 6.38(5.144-7.914) | 7.163(5.424-9.459) | 76.45155 | 63.73858 |
| Both | Niger | 1.615(1.589-1.642) | 1.703(1.619-1.792) | 1.796(1.648-1.957) | 1.893(1.677-2.137) | 1.996(1.707-2.334) | 57.40406 | 25.4993 |
| Both | Nigeria | 2.873(2.827-2.919) | 3.167(3.014-3.328) | 3.492(3.212-3.797) | 3.851(3.422-4.333) | 4.246(3.646-4.944) | 93.40228 | 51.19973 |
| Both | Sao Tome and Principe | 2.258(2.207-2.31) | 2.524(2.353-2.708) | 2.822(2.505-3.178) | 3.154(2.667-3.729) | 3.525(2.84-4.377) | 124.2167 | 59.57046 |
| Both | Sierra Leone | 2.609(2.577-2.642) | 2.945(2.834-3.061) | 3.325(3.115-3.548) | 3.753(3.424-4.114) | 4.237(3.763-4.77) | 87.91293 | 67.09375 |
| Both | Togo | 3.455(3.405-3.506) | 3.82(3.652-3.997) | 4.224(3.914-4.56) | 4.671(4.195-5.202) | 5.165(4.495-5.935) | 99.94387 | 52.87868 |
| Male | Benin | 3.764(3.697-3.831) | 4.006(3.793-4.232) | 4.265(3.888-4.679) | 4.54(3.985-5.173) | 4.833(4.085-5.719) | 86.36311 | 30.33103 |
| Male | Burkina Faso | 2.791(2.746-2.837) | 3.006(2.858-3.161) | 3.237(2.973-3.524) | 3.486(3.092-3.93) | 3.754(3.216-4.383) | 72.06943 | 37.25502 |
| Male | Cameroon | 5.942(5.876-6.009) | 6.457(6.237-6.684) | 7.015(6.617-7.437) | 7.622(7.019-8.276) | 8.281(7.446-9.21) | 85.04569 | 42.25737 |
| Male | Cabo Verde | 7.479(6.727-8.315) | 12.475(9.652-16.123) | 20.808(13.788-31.401) | 34.708(19.682-61.205) | 57.893(28.086-119.333) | 550.1548 | 758.4148 |
| Male | Chad | 2.028(1.999-2.058) | 2.121(2.027-2.219) | 2.218(2.054-2.394) | 2.319(2.082-2.583) | 2.425(2.11-2.787) | 69.22024 | 21.00237 |
| Male | Gambia | 2.613(2.454-2.783) | 2.898(2.388-3.518) | 3.215(2.317-4.459) | 3.565(2.248-5.655) | 3.954(2.18-7.171) | 101.5724 | 54.91783 |
| Male | Ghana | 5.636(5.446-5.833) | 6.537(5.88-7.267) | 7.581(6.338-9.068) | 8.792(6.832-11.316) | 10.197(7.363-14.122) | 93.2007 | 87.66856 |
| Male | Guinea | 0.953(0.942-0.965) | 0.983(0.948-1.02) | 1.014(0.954-1.079) | 1.046(0.96-1.141) | 1.08(0.965-1.207) | 54.87403 | 14.33987 |
| Male | Guinea-Bissau | 3.701(3.679-3.724) | 3.813(3.743-3.885) | 3.928(3.807-4.054) | 4.047(3.872-4.23) | 4.169(3.938-4.414) | 41.00688 | 13.31268 |
| Male | Liberia | 3.792(3.701-3.885) | 4.172(3.872-4.495) | 4.59(4.046-5.206) | 5.049(4.227-6.031) | 5.555(4.416-6.987) | 74.45383 | 49.7321 |
| Male | Mali | 2.954(2.917-2.991) | 3.08(2.964-3.2) | 3.211(3.009-3.426) | 3.348(3.055-3.668) | 3.49(3.102-3.927) | 39.10002 | 19.61092 |
| Male | Mauritania | 3.822(3.706-3.942) | 4.237(3.851-4.662) | 4.697(3.996-5.52) | 5.207(4.146-6.538) | 5.772(4.302-7.744) | 39.41851 | 55.30101 |
| Male | Niger | 1.7(1.67-1.732) | 1.747(1.651-1.849) | 1.795(1.632-1.975) | 1.845(1.613-2.11) | 1.896(1.594-2.254) | 33.9872 | 12.74669 |
| Male | Nigeria | 2.62(2.573-2.668) | 2.789(2.637-2.949) | 2.969(2.701-3.264) | 3.161(2.766-3.611) | 3.365(2.833-3.996) | 62.35107 | 30.49943 |
| Male | Sao Tome and Principe | 2.216(2.173-2.261) | 2.478(2.331-2.634) | 2.77(2.499-3.072) | 3.097(2.678-3.582) | 3.463(2.87-4.178) | 108.1126 | 59.85134 |
| Male | Sierra Leone | 2.583(2.547-2.619) | 2.864(2.742-2.99) | 3.175(2.951-3.416) | 3.52(3.175-3.903) | 3.903(3.417-4.459) | 48.67131 | 54.93645 |
| Male | Togo | 3.501(3.444-3.559) | 3.696(3.512-3.889) | 3.901(3.579-4.251) | 4.118(3.648-4.649) | 4.347(3.717-5.083) | 75.28785 | 25.71742 |
| Female | Benin | 3.131(3.093-3.169) | 3.493(3.365-3.627) | 3.897(3.658-4.152) | 4.348(3.976-4.754) | 4.851(4.322-5.444) | 155.9358 | 58.4844 |
| Female | Burkina Faso | 2.34(2.298-2.384) | 2.657(2.511-2.813) | 3.017(2.741-3.322) | 3.426(2.992-3.923) | 3.89(3.267-4.633) | 143.8076 | 71.43178 |
| Female | Cameroon | 5.301(5.244-5.359) | 5.989(5.791-6.194) | 6.766(6.393-7.162) | 7.644(7.056-8.281) | 8.636(7.788-9.576) | 135.609 | 67.40622 |
| Female | Cabo Verde | 8.188(7.819-8.574) | 9.3(8.066-10.723) | 10.564(8.305-13.436) | 11.998(8.549-16.84) | 13.628(8.799-21.108) | 899.0139 | 70.87723 |
| Female | Chad | 1.925(1.9-1.95) | 2.138(2.055-2.225) | 2.374(2.22-2.539) | 2.637(2.399-2.899) | 2.929(2.592-3.309) | 149.7566 | 55.83018 |
| Female | Gambia | 2.307(2.125-2.505) | 2.605(2.022-3.357) | 2.942(1.917-4.515) | 3.322(1.816-6.076) | 3.751(1.721-8.177) | 152.3924 | 67.04688 |
| Female | Ghana | 8.127(7.829-8.435) | 9.548(8.509-10.713) | 11.218(9.234-13.627) | 13.18(10.019-17.338) | 15.485(10.87-22.061) | 117.5881 | 98.05563 |
| Female | Guinea | 1.753(1.736-1.771) | 1.952(1.892-2.014) | 2.173(2.061-2.291) | 2.419(2.246-2.606) | 2.693(2.447-2.964) | 67.25347 | 57.50118 |
| Female | Guinea-Bissau | 3.086(3.073-3.1) | 3.369(3.323-3.415) | 3.678(3.594-3.764) | 4.015(3.886-4.148) | 4.383(4.202-4.571) | 141.2928 | 44.42062 |
| Female | Liberia | 3.21(3.165-3.255) | 3.62(3.466-3.781) | 4.083(3.794-4.395) | 4.605(4.152-5.108) | 5.194(4.544-5.938) | 117.0089 | 65.92404 |
| Female | Mali | 1.906(1.887-1.925) | 2.079(2.016-2.145) | 2.269(2.153-2.391) | 2.475(2.299-2.665) | 2.701(2.455-2.971) | 86.92213 | 44.64459 |
| Female | Mauritania | 5.223(5.077-5.374) | 5.912(5.416-6.453) | 6.691(5.77-7.759) | 7.573(6.147-9.33) | 8.571(6.547-11.22) | 119.2139 | 69.40093 |
| Female | Niger | 1.54(1.518-1.563) | 1.677(1.604-1.754) | 1.826(1.693-1.97) | 1.989(1.788-2.213) | 2.166(1.887-2.485) | 101.9027 | 43.63514 |
| Female | Nigeria | 3.109(3.063-3.156) | 3.508(3.35-3.674) | 3.958(3.661-4.28) | 4.466(4-4.986) | 5.039(4.371-5.809) | 133.8517 | 66.56434 |
| Female | Sao Tome and Principe | 2.291(2.226-2.358) | 2.559(2.342-2.798) | 2.859(2.46-3.323) | 3.195(2.585-3.949) | 3.569(2.715-4.692) | 138.7817 | 59.12337 |
| Female | Sierra Leone | 2.636(2.604-2.668) | 3.035(2.924-3.151) | 3.494(3.28-3.722) | 4.023(3.68-4.397) | 4.631(4.128-5.195) | 158.3222 | 81.42927 |
| Female | Togo | 3.37(3.325-3.417) | 3.852(3.692-4.018) | 4.402(4.099-4.727) | 5.03(4.549-5.562) | 5.749(5.049-6.545) | 127.6873 | 75.57463 |


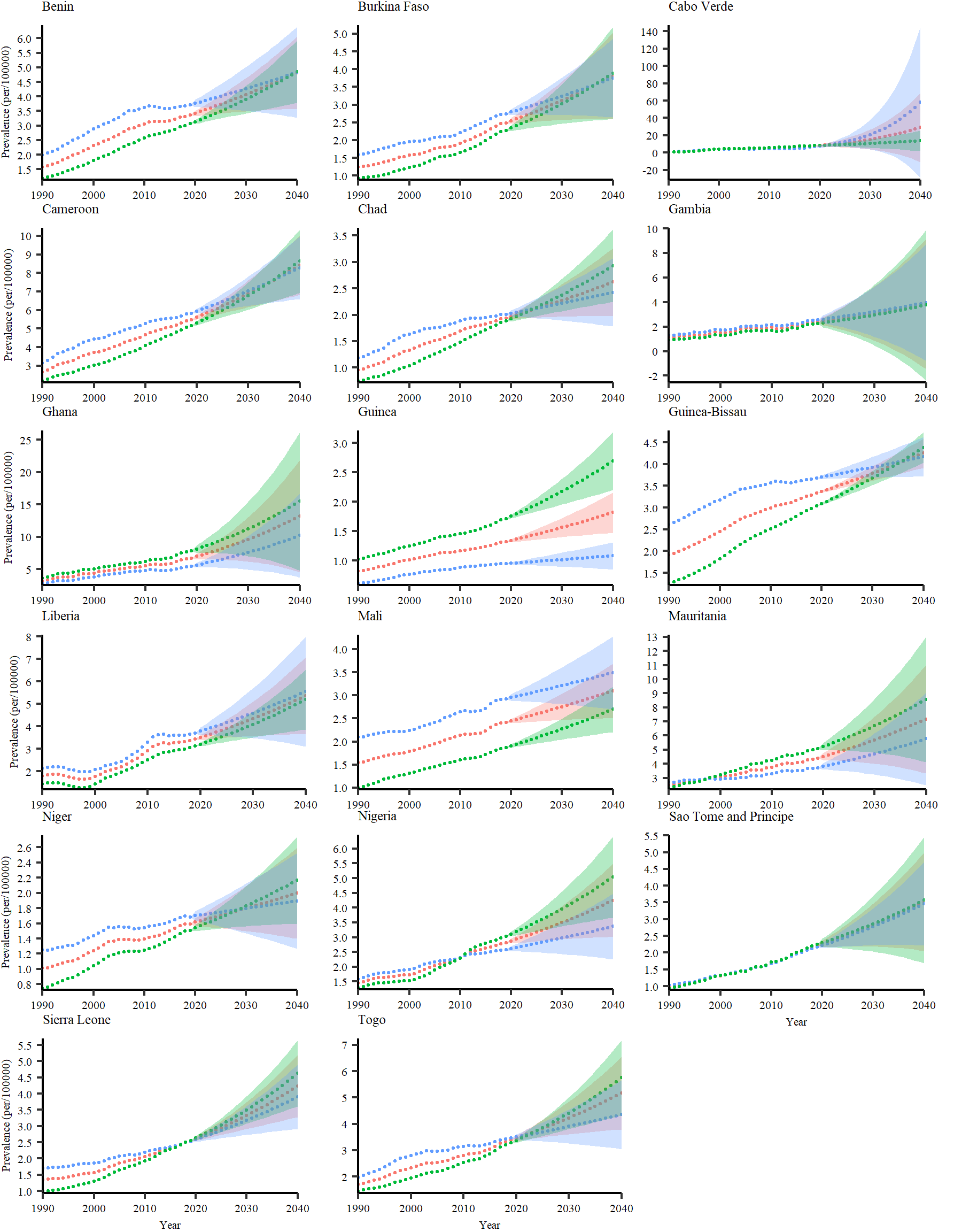


Supplemental Figure 42. Observed and projected age-standardized prevalence rate (ASPR) values from 1990 to 2040 for both sex (Red lines), females (Green lines), and men (Blue lines) in the Western Sub-Saharan Africa. The halo effect observed in each scatter plot accurately represents projections that extend across the temporal span from 2019 to 2040 with 95% confidence intervals.


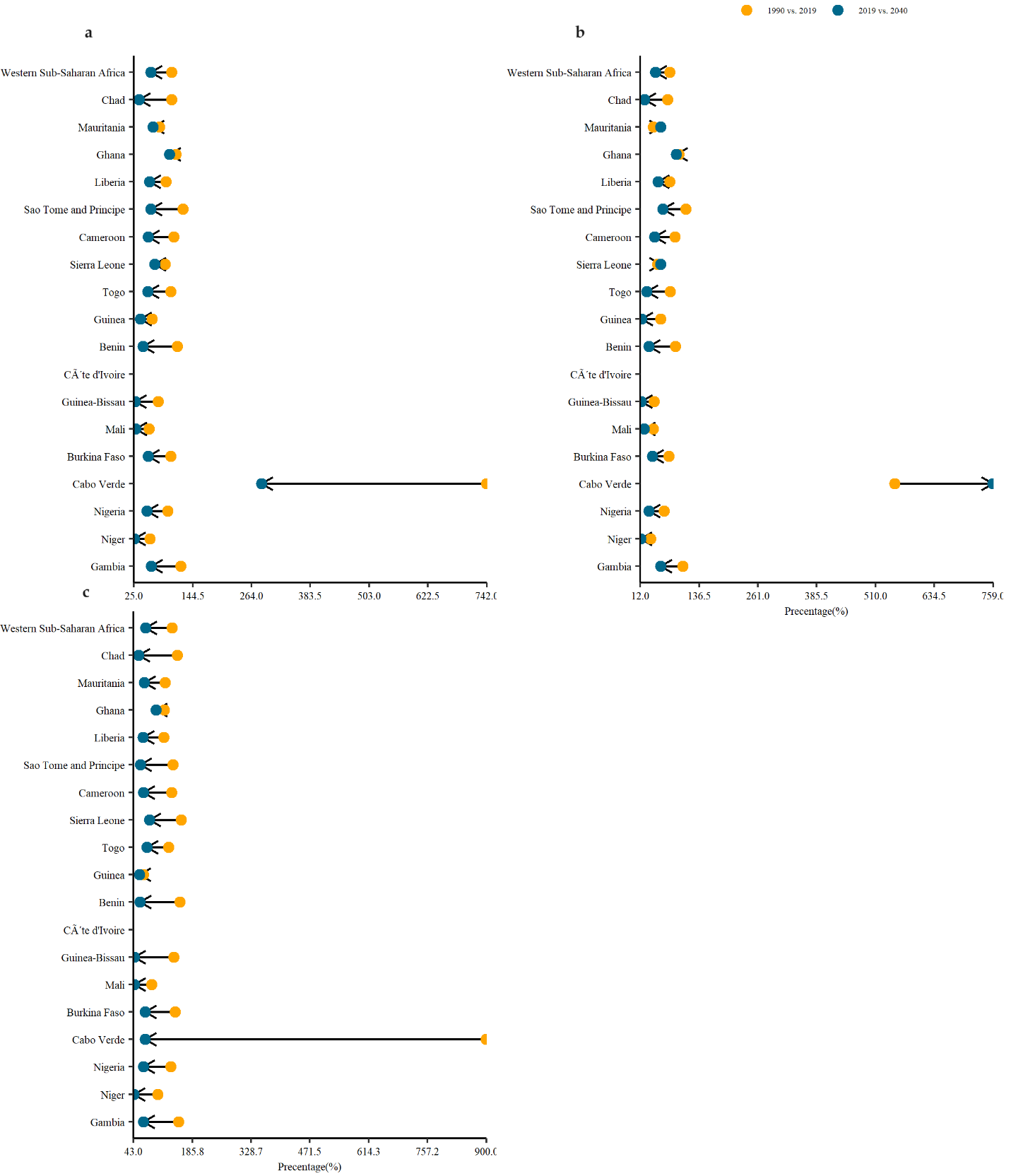


Supplemental Figure 43. The Lollipop plot between the two calculated percentage changes from 1990 to 2019 and 2019 to 2040 for both sexes (a), males (b), and females (c) in the Western Sub-Saharan Africa. Each line represents two time periods and show the change of ASPR increase or decrease during time.
